# Metal-free reductive desulfurization of C-sp^3^-substituted thiols using phosphite catalysis†

**DOI:** 10.1039/d3sc00045a

**Published:** 2023-07-13

**Authors:** Rana M. I. Morsy, Ganesh Samala, Ankur Jalan, Michael E. Kopach, Naresh M. Venneti, Jennifer L. Stockdill

**Affiliations:** a Department of Chemistry, Wayne State University Detroit MI 48202 USA gu0132@wayne.edu stockdill@wayne.edu; b Eli Lilly and Company Indianapolis IN 46285 USA

## Abstract

Phosphines and phosphites are critical tools for non-metal desulfurative methodologies due to the strength of the P=S bond. An overarching premise in these methods has been that stoichiometric (or excess) P(iii) reagent is required for reactivity. Despite decades of research, a desulfurative process that is catalytic in phosphine/phosphite has not been reported. Here, we report the successful merging of two thermal radical processes: the desulfurization of unactivated and activated alkyl thiols and the reduction of P(v) = S to P(iii) by reaction with a silyl radical species. We employ catalytic trimethyl phosphite, catalytic azo-bis(cyclohexyl)nitrile, and two equivalents of tris(trimethylsilyl)silane as the stoichiometric reductant and sulfur atom scavenger. This method is tolerant of common organic functional groups and affords products in good to excellent yields.

## Abstract 2

Catalytic phosphite radical desulfurization: trimethyl phosphite serves as a sulfur trapping agent in thermal radical desulfurization, using TTMSS as the terminal reductant. The conversion of P(v) = S to P(iii) proceeds through a tetravalent phosphoryl radical intermediate.

## Graphical abstract

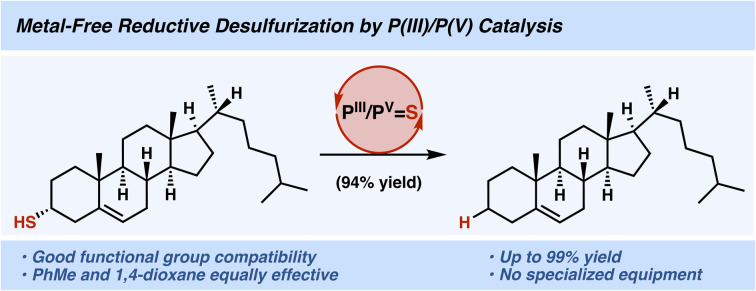

## Introduction

Desulfurization is an important process^1^ for diverse applications from organic synthesis^2^ and chemical protein synthesis^2^ to crude oil processing and refinement.^3^ Numerous reports describe transition metal-based reduction reactions, including Ni,^4^ Co,^5^ W,^6^ Mo,^7^ and others.^8^ Unfortunately, critical limitations remain, which include pyrophoricity,^4,5^ toxic H_2_S gas evolution,^4^ reactivity with other S-functional groups,^4,6^ and the need for specialized reaction setups.^5^ Additionally, when the desulfurized products are deployed in living systems, trace metal contamination can be problematic.^9^ Photoredox strategies^10*a*,*b*^ avoid functional group incompatibility^10*a*^ and may enable further functionalization of the substrate; however, they do require the use of expensive rare earth transition metal complexes, which may limit industrial scale applications. With only two exceptions,^10*d*,*e*^ modern and non-metal strategies for thiol desulfurization^10,11^ employ excess phosphine/phosphite reagents, whose aquatic toxicity^9^ has limited their utility in >kg-scale processes. The last several years have seen a surge of interest in the potential of P(iii)/P(v) catalysis,^12^ largely focused on strategies to facilitate polar P(iii)/P(v) = O cycles.^13^ We were intrigued by the potential of employing P(iii)/P(v) = S catalysis for desulfurative radical transformations. Here, we present the first step along this journey, a metal-free desulfurization of unactivated thiols by P(iii)/P(v) = S catalysis. We achieve this reactivity by leveraging the affinity of silyl radicals toward sulfur^14^ to achieve *in situ* regeneration of phosphites by reduction of the P=S bond while sequestering the S atom to avoid H_2_S formation.

## Results and discussion

Reports as recent as 2021 have asserted that stoichiometric phosphine is a requirement for radical desulfurization reactions.^12*b*^ Both phosphines and phosphites are well established reagents for desulfurization reactions,^11,15^ with the electronics of the R group on PR_3_ being key to reactivity.^16^ Phosphine sulfides react with super silyl hydride (TTMSS)^17^ under free radical conditions to produce phosphines in good yields.^14^ Unlike P(iii)/P(v) = O redox cycles, which occur *via* polar pathways, the P(iii)/P(v) = S reduction occurs *via* a radical mechanism.^14^ Thus, we envisioned that this cycle would merge seamlessly with a radical desulfurization mechanism. We employed Ac-*N*-Cys-OMe^18^ (1a) as a model substrate to investigate the feasibility of the catalytic transformation in the presence of various phosphines/phosphites with TTMSS as a phosphine sulfide reductant^14^ and/or terminal hydrogen atom donor^19^ (Table 1). We selected azo-bis(cyclohexyl)nitrile (ACHN) as the initiator because of its 10 h half-life^20^ and to avoid the formation of oxygenated by-products commonly observed in the presence of organic peroxide initiators.^21^ Because of the electrophilic nature of thiyl radicals, ^22^ we began our studies with 10 mol% of the electron-rich tris(dimethylanion)phosphine ((Me_2_N)_3_P).^23^ Only a single turnover was observed, with the desired product (1b) formed in 21% conversion (entry 1). We limited reaction time to 24 h for the purposes of this screen. The less basic tri-*tert*-butylphosphine was markedly better with 65% conversion (entry 2), while the less hindered and less electron-rich tri(*n*-butyl)phosphine led to 54% conversion (entry 3). Interestingly, tri-cyclohexylphosphine, which bridges these steric and electronic properties, was even less effective (38% conversion, entry 4). Trimethyl phosphite balanced these effects, improving the conversion to 80% (entry 5). Increasing the phosphite loading to 20 mol% led to complete conversion by 16 h (entry 6).^24^ Control experiments were consistent with the radical pathway we envisioned for this chemistry. Omission of the radical initiator (entry 7) or the phosphite (entry 8) abolished reactivity. In the absence of TTMSS, slow conversion was observed and did not reach 20% because the substrate thiol must serve as the H-atom donor for C–H bond formation (entry 9). Finally, heating is required for the reduction of the P=S bond by TTMSS, as evidenced by entry 10.

**Table 1:** Establishing PR_3_ reactivity under catalytic conditions^a^

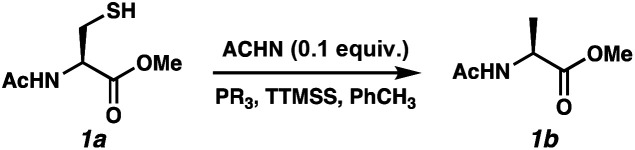						
Entry	PR_3_ (equiv.)	TTMSS (equiv.)	ACHN (equiv.)	Temp. (°C)	Time (h)	Conversion^b^ (%)
1	(Me_2_N)_3_P (0.1)	1	0.1	80	24	21
2	^*t*^Bu_3_P (0.1)	1	0.1	80	24	65
3	^*n*^Bu_3_P (0.1)	1	0.1	80	24	54
4	P(Cy)_3_ (0.1)	1	0.1	80	24	38
5	P(OMe)_3_ (0.1)	1	0.1	80	24	80
**6**	**P(OMe)**_**3**_ **(0.2)**	**2**	**0.1**	**88**	**16**	**>99**
7	P(OMe)_3_ (0.2)	2	—	88	16	0
8	—	2	0.1	88	16	0
9	P(OMe)_3_ (0.2)	—	0.1	88	16	7
10	P(OMe)_3_ (0.2)	2	0.1	25	16	0

a Reactions were performed with 1 equiv. 1a (0.05 M) in degassed PhMe.

b Conversion = consumption of 1a; based on ^1^H-NMR integration.

We proceeded with trimethyl phosphite at 88 °C for 16 h while screening different reaction parameters such as initiator, reductant, and solvent (Table 2). Using different initiators like VA-044 or Luperox A98 led to large decreases in conversion (entries 1–2). Dicumyl peroxide led to a 3-fold increase in conversion percentage relative to VA-044 at the same temperature (entry 3), but it was still inferior to ACHN.

**Table 2:** Probing the impact of radical initiator, reductant, and solvent^a^

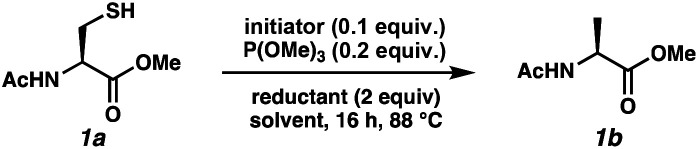				
Entry	Initiator	Reductant	Solvent	Conversion^b^ (%)
1	VA-044	TTMSS	H_2_O/MeCN	44
2	Luperox A98	TTMSS	PhMe	22
3	Dicumyl peroxide	TTMSS	PhMe	65
4	ACHN	TIPS	PhMe	7
5	ACHN	Et_3_GeH	PhMe	43
6	ACHN	^*n*^Bu_3_SnH	PhMe	63
7	ACHN	TTMSS	MeCN	62
8	ACHN	TTMSS	THF	82
**9**	**ACHN**	**TTMSS**	**1,4-Dioxane**	**>99**

a Reactions were performed with 1 equiv. 1a (0.05 M) in degassed PhMe.

b Conversion = consumption of 1a; based on ^1^H-NMR integration.

Alternative hydrogen atom/silyl radical donors had a deleterious effect on conversion. When tri-isopropylsilane (TIPS) was used, the reaction takes place, but the phosphite is not reduced (Table 2, entry 4). This apparent limited efficiency of TIPS compared to TTMSS is logical given the difference in their Si–H bond-dissociation energies (BDEs) with trialkylsilanes ranging from (90–96 kcal mol^−1^)^25^ compared to the Si–H in TTMSS at 79 kcal mol^−1^.^26^ The instability of the TIPS˙ interferes with the turnover of the phosphine sulfide, presumably because H-atom abstraction from the substrate or solvent occurs more rapidly than Si–S bond formation. Alternatively, the i-Pr group is slow to migrate after α-scission. Consistent with this reasoning, and with the similar properties of Ge and Si,^27^ Et_3_GeH (86 kcal mol^−1^) was able to effect ∼1 phosphine sulfide turnover in 16 h, proceeding with 43% conversion (entry 5). The best alternate P=S reductant was *n*-Bu_3_SnH (74 kcal mol^−1^), which still reduced the phosphine sulfide more slowly than TTMSS (entry 6).^28^ The respective BDEs of TTMSS and *n*-Bu_3_SnH indicate that the Si˙ and Sn˙ species are similarly stable and the rate difference likely arises from differences in affinity to sulfur or the ability of TTMSS to irreversibly sequester the S-atom *via* an additional α-fragmentation and 1,2-group shift (see Scheme 2).

In terms of solvent, THF and MeCN, each of which were performed at 88 °C using a sealed microwave tube, were less competent than toluene (entries 7–8). Interestingly, the reaction proceeded to complete conversion with 1,4-dioxane as solvent (entry 9). Indeed, the success of 1,4-dioxane increases the scope of this method to include polar substrates.

With two sets of established conditions in hand, we evaluated the scope of our efficient reductive desulfurization reaction on different relevant substrates, including short peptides and small molecule substrates that would form intermediate C-radicals with varying stability (Table 3). Both protected dipeptide 2a and tripeptide 3a – each containing a 1° alkyl thiol – were desulfurized in 86% yield. Carboxylic acid-containing captopril (4a) was desulfurized in 79% yield. Tripeptide 5a performed more sluggishly, producing 5b in 63% yield with 32% recovered 5a. We next investigated 2° and 3° alkyl thiol substrates. Cholesterol derivative 6a was converted to cholest-5-ene 6b in 94% yield. The B-ring olefin was unaffected. Penicillamine derivatives 7a and 8a proceed through tertiary C-radicals to afford valine-containing peptides 7b (79% yield) and 8b (76% yield). Notably, the thioether of methionine was stable to these conditions. Finally, we tested substrates that proceed through α-stabilized radicals. Trityl thiol 9a was desulfurized to form triphenylmethane (9b) in 97% yield. The thiols in diacid 10a and glucose derivative 11a were readily converted to the corresponding C–H bonds to provide 10b in 89% yield and a chiral pyran derivative (11b) in 99% yield.

**Table 3:** Strategic substrate scope for catalytic desulfurization

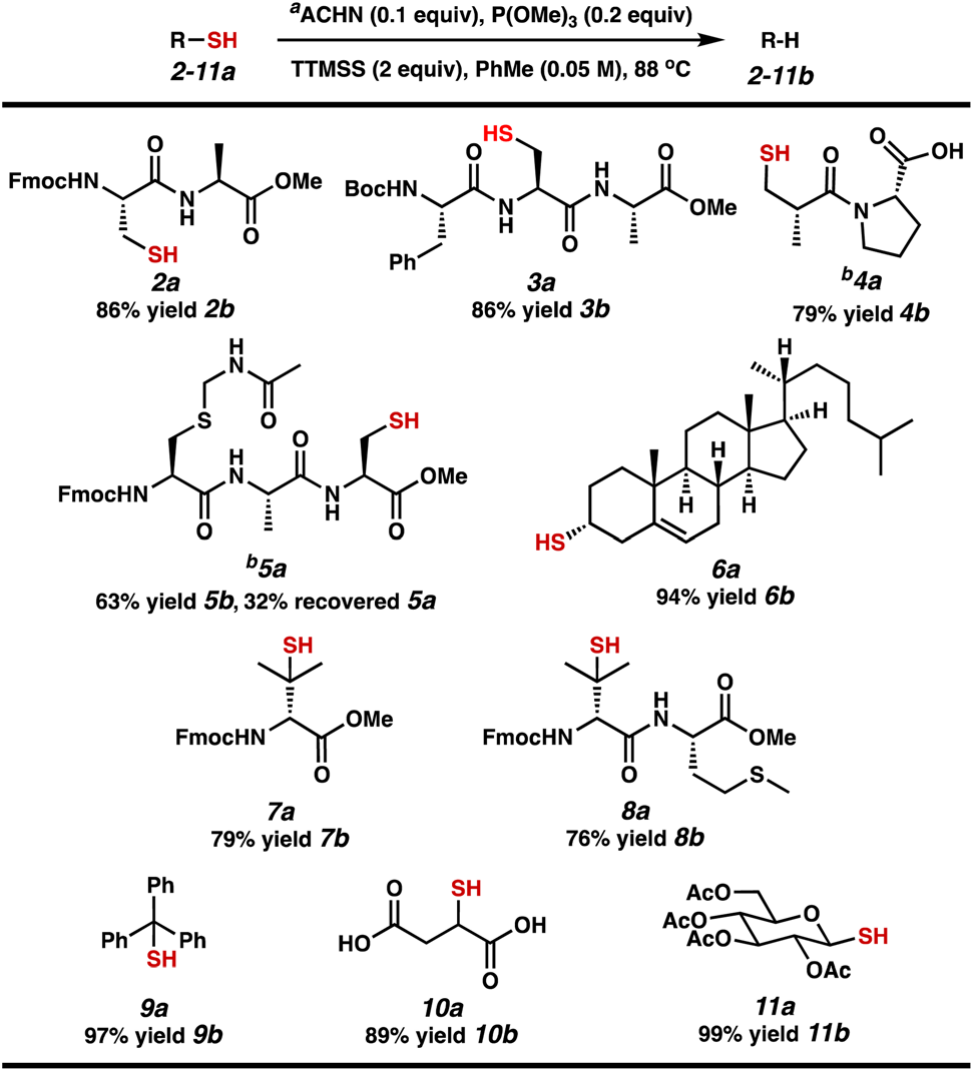

a Reactions were performed with 1 equiv. 2a–11a in degassed toluene. Yields are of isolated material following purification *via* silica gel flash chromatography or HPLC.

b Reaction was performed in 1,4-dioxane.

Efforts to extend these results to longer and unprotected peptides were not fruitful due to lack of solubility of the starting materials. A brief co-solvent screen of a dipeptide acid (Fmoc-Cys-Ala-OH, SI-13†) was conducted to probe the conversion efficiency in the presence of various amounts of water, which would be needed to increase the efficiency of the desulfurization process for peptide substrates. Water is tolerated in the reaction, with the caveat that the reactivity is significantly slowed and by-product formation occurs (disulfide, thioether, and C–C bond formation were observed).^23^ The maximum conversion obtained for dipeptide SI-13† was 62%, requiring 2 additions of ACHN and P(OMe)_3_. We suspect that the hydrophobicity of TTMSS is limiting in these reactions. Presently, a water-soluble analog of TTMSS is not available, and this chemistry is therefore best suited to organic-soluble substrates. Importantly, our interest was not in achieving an ideal peptide desulfurization, ^10*e*^ but rather in demonstrating P(iii)/P(v) = S catalysis and learning about its behavior.

To probe the amenability of the desulfurization to large scale conditions, we performed the reaction on 4-methoxybenzyl mercaptan (12a). On 6.84 mmol scale in 1,4-dioxane, 100% conversion was observed in 3 h, with clean reactivity and no unexpected by-product formation (Scheme SI-01†). To probe the compatibility of various functional groups with the reaction conditions, we again employed 4-methoxybenzyl mercaptan (12a) in toluene along with different additives. The additives shown in Table 4 were compatible with the reaction conditions, showing no impact on the additive. In most cases, good to excellent reactivity of 12a was maintained. In entries 6 and 8, the desulfurization reaction did not occur. Alcohol additives had no negative effect on the desulfurization but were silylated. Aldehyde and ketone additives led to an unknown by-product and interfered somewhat with the desulfurization (see Table SI-02†).

**Table 4:** Functional group compatibility screen

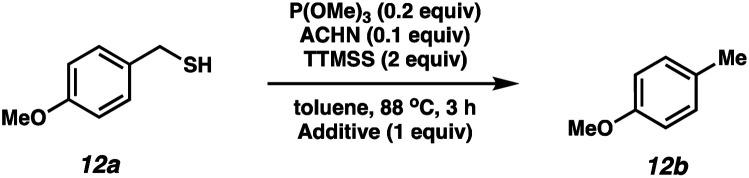							
Entry^a^	Additive	Unreacted 12a^b^ (%)	GC yield 12b^b^ (%)	Entry^a^	Additive	Unreacted 12a^b^ (%)	GC yield 12a^b^ (%)
1	None	0	87	6	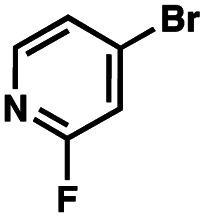	100	0
2	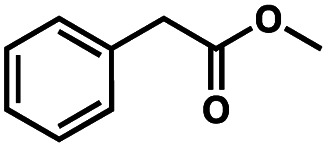	0	87	7	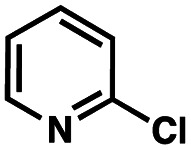	0	57
3	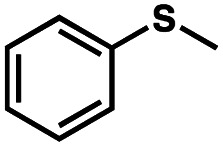	7	91	8	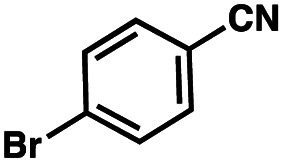	95	4
4	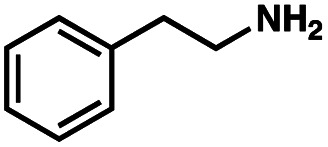	0	93	9	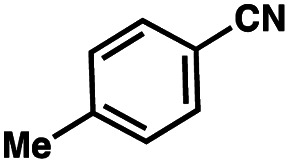	0	81
5	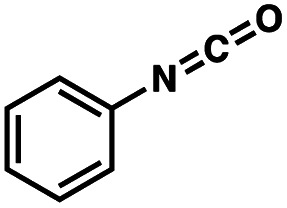	0	59	10	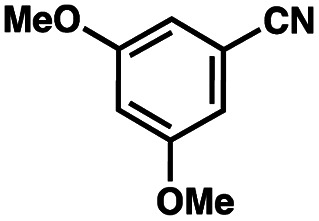	0	55

a Reaction conditions: (4-methoxyphenyl)methanethiol (0.2 mmol, 1 equiv.), P(OMe)_3_ (0.04 mmol, 0.2 equiv.), ACHN (0.02 mmol, 0.1 equiv.), TTMSS (0.4 mmol, 2 equiv.), toluene (0.05 M), 88 °C, 3 h.

b Calculated *via* GCMS using 1,3,5-trimethoxybenzene as internal standard.

**Scheme 1:**
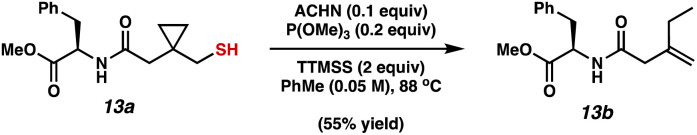
Cyclopropylcarbinyl radical rearrangement product supports a radical pathway.

**Scheme 2:**
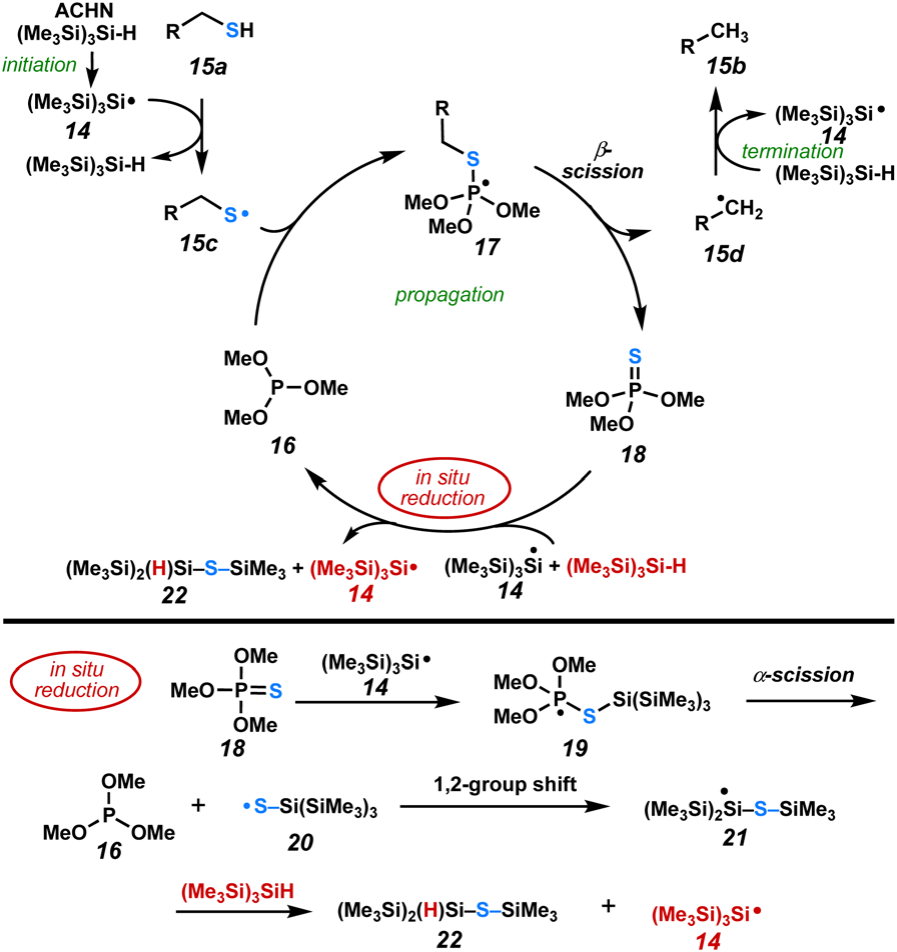
Mechanism of catalytic desulfurization.

A radical clock experiment (Scheme 1) was performed with cyclopropane-containing thiol, 13a, which underwent ring opening to afford olefin 13b in 55% yield. This indicates that a carbon-centered radical is an intermediate in this reaction. Based on our observations, we hypothesize that reduction of the phosphine sulfide is rate-limiting and proceeds *via* the radical pathway described by Chatgilialoglu.^14^ The postulated overall radical chain mechanism for the catalytic reductive desulfurization is illustrated in Scheme 2.

Upon heating, ACHN releases N_2_ to initiate the reaction by forming the C-centered cyclohexyl nitrile radical. H-atom abstraction from TTMSS leads to super silyl radical 14. H-atom abstraction from the sulfhydryl group in 15a forms thiyl radical 15c and regenerates TTMSS.^22^ Thiyl radical 15c reacts with phosphite^29^16 to generate phosphoranyl radical intermediate 17.^30^ Tetravalent P-centered radical 17 undergoes β-scission to produce trimethyl thiophosphate 18 and the carbon centered radical 15d. H-atom transfer from TTMSS to 15d produces the desulfurized product 15b and regenerates TTMSS radical 14. Trimethyl thiophosphate 18 reacts with silyl radical 14 to form phosphoranyl radical intermediate 19. Subsequent α-scission regenerates the catalytically active phosphite (16) to be reused in the propagation step. Notably, this α-scission is facilitated by the thiophilic nature of the silane (Si–S 148.4 *versus* P–S 105.6 kcal mol^−1^).^25^ The resulting S-centered radical 20 induces a 1,2-group shift^31^ of a TMS group from silicon to sulfur, furnishing silyl radical 21 that reacts with a second equivalent of TTMSS to afford TTMSS radical 14 and silyl sulfide 22.^32^ This completes the *in situ* reduction process and permits propagation of the radical cascade. GCMS analysis of the crude reaction mixture indicates the presence of a small amount of phosphine sulfide 18 and multiple TTMSS-related peaks during the reaction.

## Conclusions

In summary, we have established reaction conditions for P(iii)/P(v) = S catalysis that proceeds through a tetravalent P-centered radical rather than through a pentavalent P(v) intermediate as is observed for P=O reduction. The reductive desulfurization reaction sequesters the S atom, avoids the use of rare earth and transition metals, and affords desulfurized products in good yields. The method has been tested across a range of substrate types including thiols that lead to benzylic, primary, secondary, and tertiary alkyl C-centered radicals. Importantly, good functional group compatibility is observed, including free carboxylic acid, ester, carbamate, amide, nitrile, aryl halide, amine, isocyanate, thioether functionalities. Steroid, carbohydrate, amino acid, pyridine, and benzene scaffolds are compatible with the reaction. No special setup is required for this catalytic method, so it can be readily employed and adapted. This study expands the scope of metal-free and thiol-additive-free desulfurization methods while establishing the viability of P(iii)/P(v) = S catalysis under radical conditions. Efforts to harness this catalysis strategy to effect other transformations and develop new phosphorus-based catalysts are ongoing.

## Data availability

The datasets supporting this article have been uploaded as part of the ESI.†

## Author contributions

All authors have contributed to this research.

## Conflicts of interest

There are no conflicts to declare.

## Supplementary Material

SC-014-D3SC00045A-s001
