# Supplementary material for: Metal-free reductive desulfurization of C-sp3-substituted thiols using phosphite catalysis
Source: Chem Sci. 2023 Jul 13;14(34):9016–23. doi: 10.1039/d3sc00045a (PMC10466286; doi:10.1039/d3sc00045a)
Supplement: SC-014-D3SC00045A-s001 [file SC-014-D3SC00045A-s001.pdf]

Electronic Supplementary Material (ESI) for Chemical Science.
This journal is © The Royal Society of Chemistry 2023

Supporting Information

Metal-free reductive desulfurization of C-sp^3-substituted thiols using phosphite catalysis

Rana M. I. Morsy,[a] Ganesh Samala,[a] Ankur Jalan,[b] Michael E. Kopach,[b] Naresh M. Venneti,[a] Jennifer L. Stockdill*[a]*

Table of Contents

General Information S2
General procedure for catalytic desulfurization S3
Experimental procedure and spectroscopic data for Table 1 .... S3
Experimental procedure and spectroscopic data for Table 2 .... S8
Experimental procedure and spectroscopic data for Table 3 .... S13
Experimental procedure and spectroscopic data for Scheme 1 .... S21
Peptide desulfurization solvent screening data for Table *SI-01* S24
Additives screening data for Table 4 .... S28
Additives screening data for Table *SI-02* S35
Gram Scale Catalytic Desulfurization Scheme-*SI-01* S39
Spectra S40

General Information. Unless otherwise specified, all commercially available reagents were purchased from Sigma-Aldrich and used without further purification. Anhydrous PhMe was purchased from Fisher and THF was purchased from Sigma-Aldrich. These were passed through a commercial solvent purification system (2 columns of alumina) and used without further drying. MeCN and dioxane were purchased from Sigma-Aldrich and Fisher respectively. Hexanes, ethyl acetate, water, CH_2Cl_2 and methanol were purchased from Fisher. $P(OMe)_3$, TTMSS and ACHN were purchased from Sigma-Aldrich. Unless otherwise noted, all reactions were performed in dried glassware under 1 atm of pre-purified anhydrous N_2 or Ar gas. All RP-HPLC analyses and purifications were performed on a Reverse Phase Shimadzu Liquid Chromatograph Mass Spectrometer (LCMS-2020) equipped with a photodiode array (PDA) detector (D2). The extracted wavelength is indicated in the data. RP-HPLC-MS mobile phases (MeCN and H_2O) contained 0.1% formic acid. Deuterated solvents were purchased from Cambridge Isotope Laboratories. NMR tubes were purchased from Sigma-Aldrich. Mestrelab MestReNova NMR processing software was used to process and read ^1H NMR spectra and ^{13}C NMR spectra, which were recorded on a Varian MR-400, Agilent MR-400, Varian V-500, or an Agilent DD2-600 MHz instrument with a multi-nuclear broadband probe at ambient temperature (unless otherwise stated). Chemical shifts are reported in parts per million relative to residual solvent peaks (*Organometallics* **2010**, *29*, 2176). All ^{13}C spectra are proton decoupled. High-resolution mass spectrometry was performed by the Lumigen Instrument Center, Wayne State University. Thin layer chromatography (TLC) was performed using glass backed SiliaPlate™ TLC plates cut to the desired size then visualized with short-wave UV lamps and $KMnO_4$, CAM, PMA, or Anisaldehyde stains prepared according to standard recipes. All yields refer to chromatographically and spectroscopically pure products.

General procedure for catalytic desulfurization:

In a 20 mL vial, start material was dissolved in PhMe. TTMSS was added. Vial was closed with rubber septum and N_2 or Ar was bubbled for few min. Phosphine and ACHN were added. Vial was sealed and placed in a pre-heated silicon oil bath. After the reaction time, reaction mixture was evaporated using rotary evaporator under the fume hood, and crude product was analyzed using ^{1}H-NMR.

Experimental and spectroscopic data of Table 1.

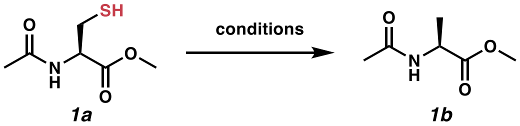

Entry 1, Table 1 (RMI-XI-059): ***1a*** (0.05 g, 0.282 mmol, 1 equiv), PhMe (5.6 mL, 0.05 M), TTMSS (0.087 mL, 0.282 mmol, 1 equiv), ACHN (0.0069 g, 0.028 mmol, 0.1 equiv), $(Me_2N)_3P$ (0.005 mL, 0.028 mmol, 0.1 equiv). Temperature 80 °C. Time 24h. Crude ^{1}H-NMR showed 21% conversion.

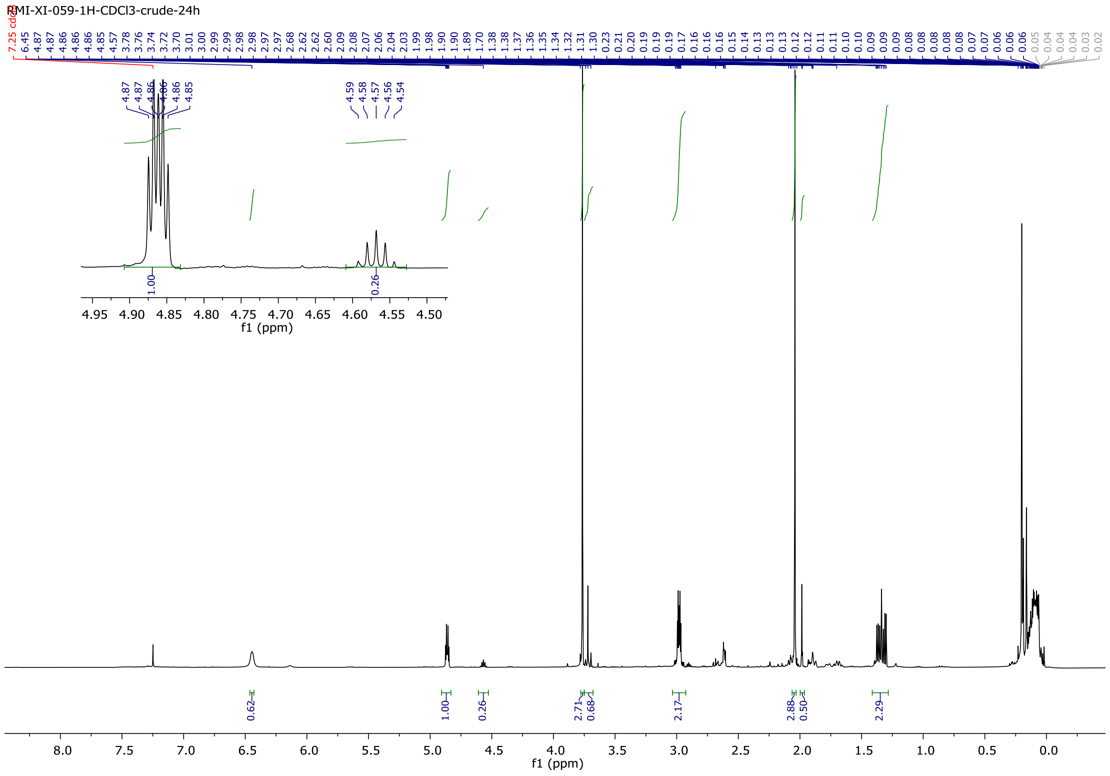

Figure *SI.1.* Crude ^{1}H-NMR of entry 1, Table 1.

Entry 2, Table 1 (RMI-XI-062): ***1a*** (0.05 g, 0.282 mmol, 1 equiv), PhMe (5.6 mL, 0.05 M), TTMSS (0.087 mL, 0.282 mmol, 1 equiv), ACHN (0.0069 g, 0.028 mmol, 0.1 equiv), $^tBu_3P$ (0.0068 mL, 0.028 mmol, 0.1 equiv). Temperature 80 °C. Time 24 h. Crude ^{1}H-NMR showed 65% conversion.

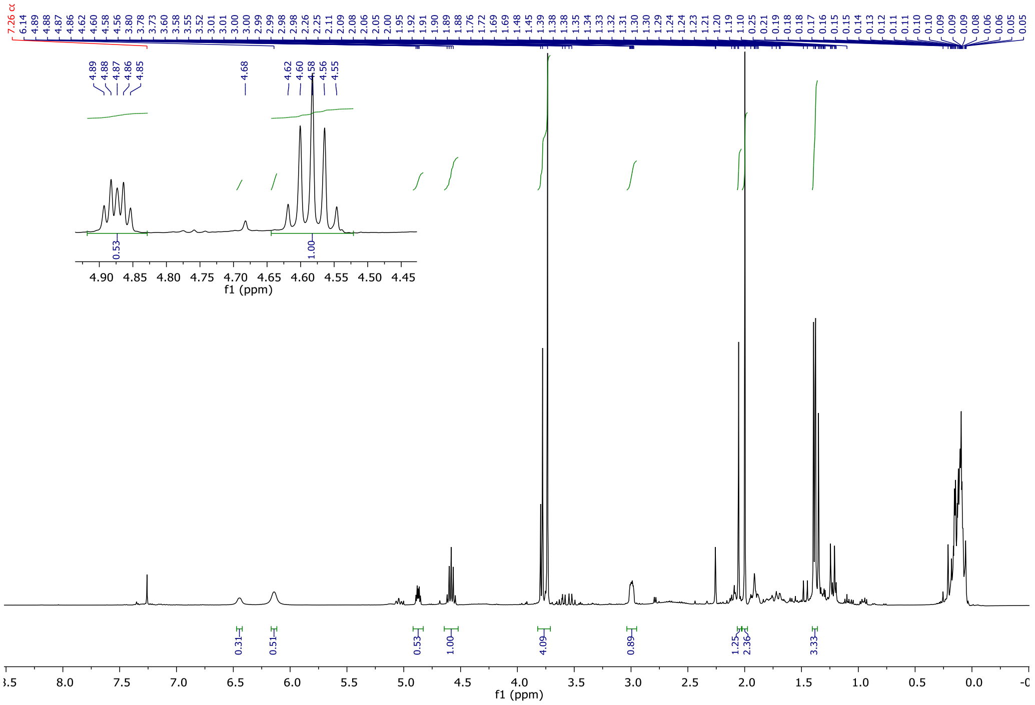

Figure *SI.2*. Crude ^{1}H-NMR of entry 2, Table 1.

Entry 3, Table 1 (RMI-XI-060): ***1a*** (0.05 g, 0.282 mmol, 1 equiv), PhMe (5.6 mL, 0.05 M), TTMSS (0.087 mL, 0.282 mmol, 1 equiv), ACHN (0.0069 g, 0.028 mmol, 0.1 equiv), $^nBu_3P$ (0.007 mL, 0.028 mmol, 0.1 equiv). Temperature 80 °C. Time 24h. Crude ^{1}H-NMR showed 54% conversion.

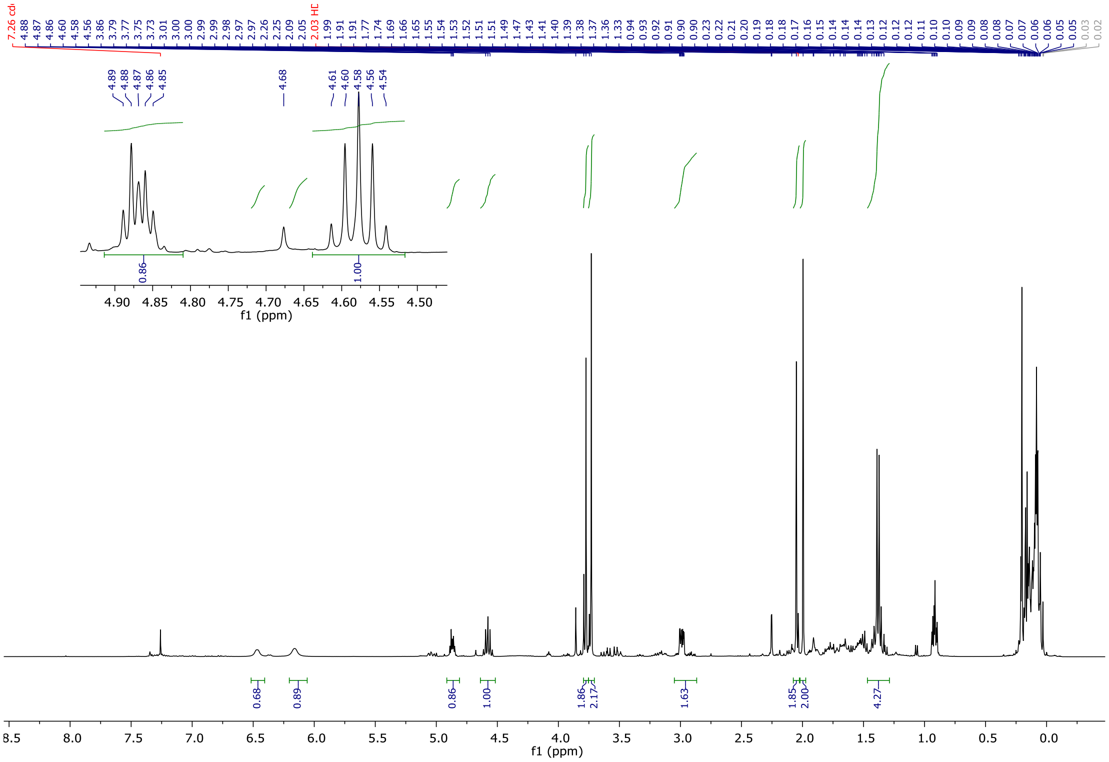

Figure *SI.3*. Crude ^{1}H-NMR of entry 3, Table 1.

Entry 4, Table 1 (RMI-XI-061): ***1a*** (0.05 g, 0.282 mmol, 1 equiv), PhMe (5.6 mL, 0.05 M), TTMSS (0.087 mL, 0.282 mmol, 1 equiv), ACHN (0.0069 g, 0.028 mmol, 0.1 equiv), $P(Cy)_3$ (0.0079 g, 0.028 mmol, 0.1 equiv). Temperature 80 °C. Time 24h. Crude ^{1}H-NMR showed 38% conversion

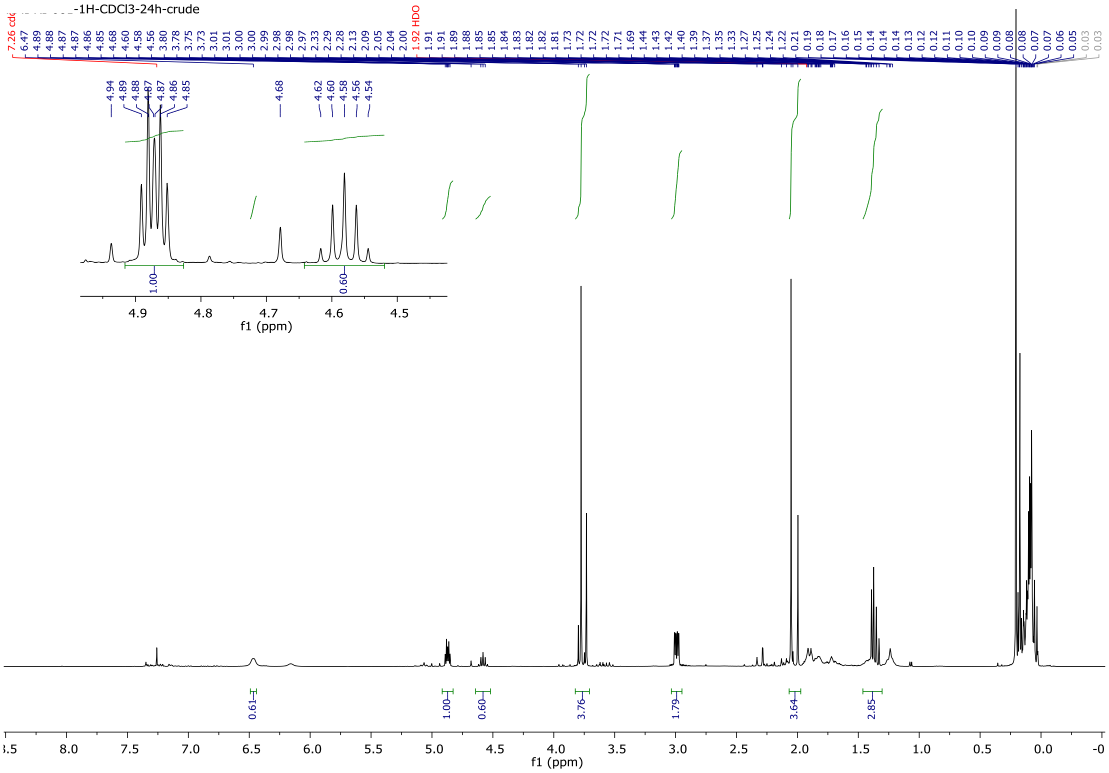

Figure *SI.4*. Crude ^{1}H-NMR of entry 4, Table 1.

Entry 5, Table 1 (RMI-XI-058): ***1a*** (0.05 g, 0.282 mmol, 1 equiv), PhMe (5.6 mL, 0.05 M), TTMSS (0.087 mL, 0.282 mmol, 1 equiv), ACHN (0.0069 g, 0.028 mmol, 0.1 equiv), $P(OMe)_3$ (0.0033 mL, 0.028 mmol, 0.1 equiv). Temperature 80 °C. Time 24h. Crude ^{1}H-NMR showed 80% conversion

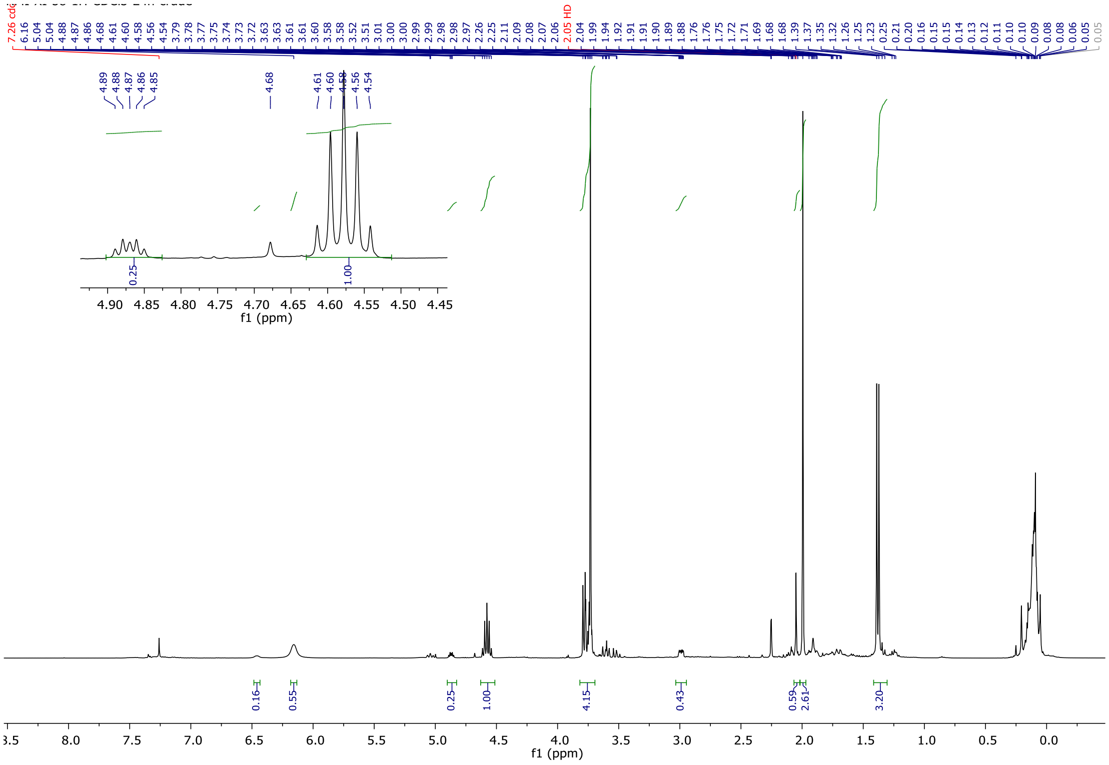

Figure *SI.5*. Crude ^{1}H-NMR of entry 5, Table 1.

Entry 6, Table 1 (RMI-XI-063): ***1a*** (0.05 g, 0.282 mmol, 1 equiv), PhMe (5.6 mL, 0.05 M), TTMSS (0.174 mL, 0.564 mmol, 2 equiv), ACHN (0.0069 g, 0.028 mmol, 0.1 equiv), $P(OMe)_3$ (0.0066 mL, 0.056 mmol, 0.2 equiv). Temperature 88 °C. Time 16 h. Crude ^{1}H-NMR showed > 99% conversion.

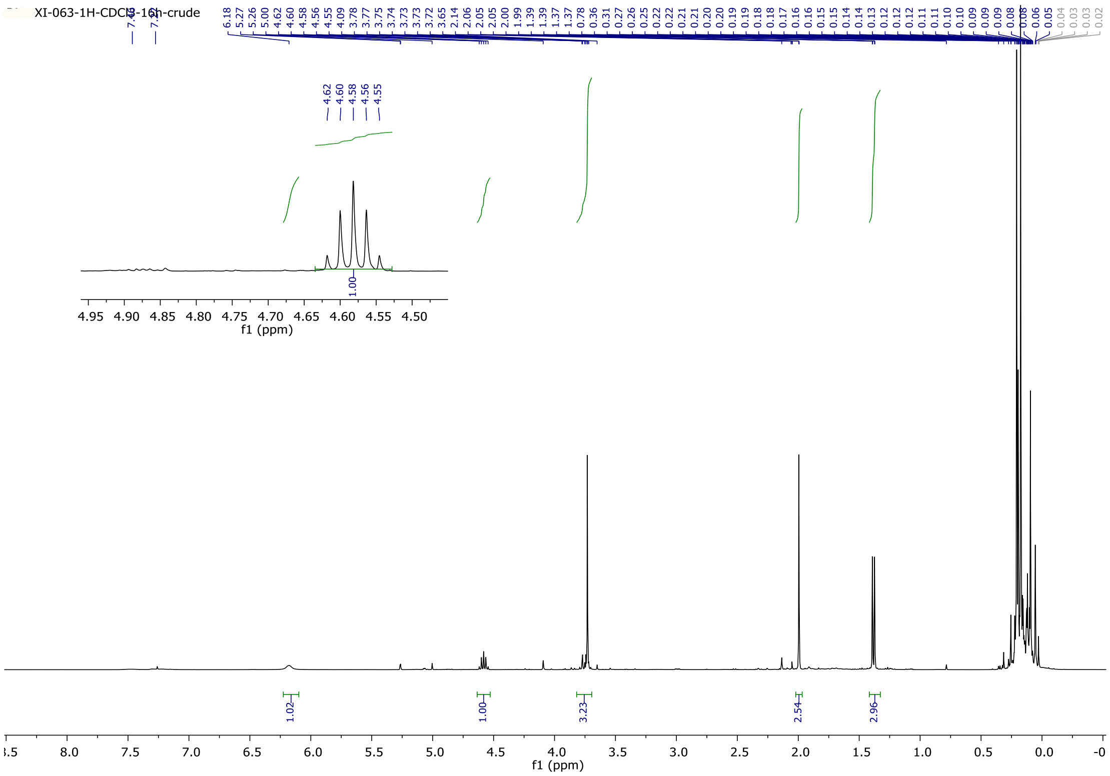

Figure *SI.6*. Crude ^{1}H-NMR of Entry 6, Table 1.

Entry 7 (No ACHN), Table 1 (RMI-VIII-025): ***1a*** (0.09 g, 0.508 mmol, 1 equiv), PhMe (10 mL, 0.05 M), TTMSS (0.31 mL, 1 mmol, 2 equiv), $P(OMe)_3$ (0.012 mL, 0.102 mmol, 0.2 equiv). Temperature 88 °C. Time 16h. Crude ^{1}H-NMR showed 0% conversion.

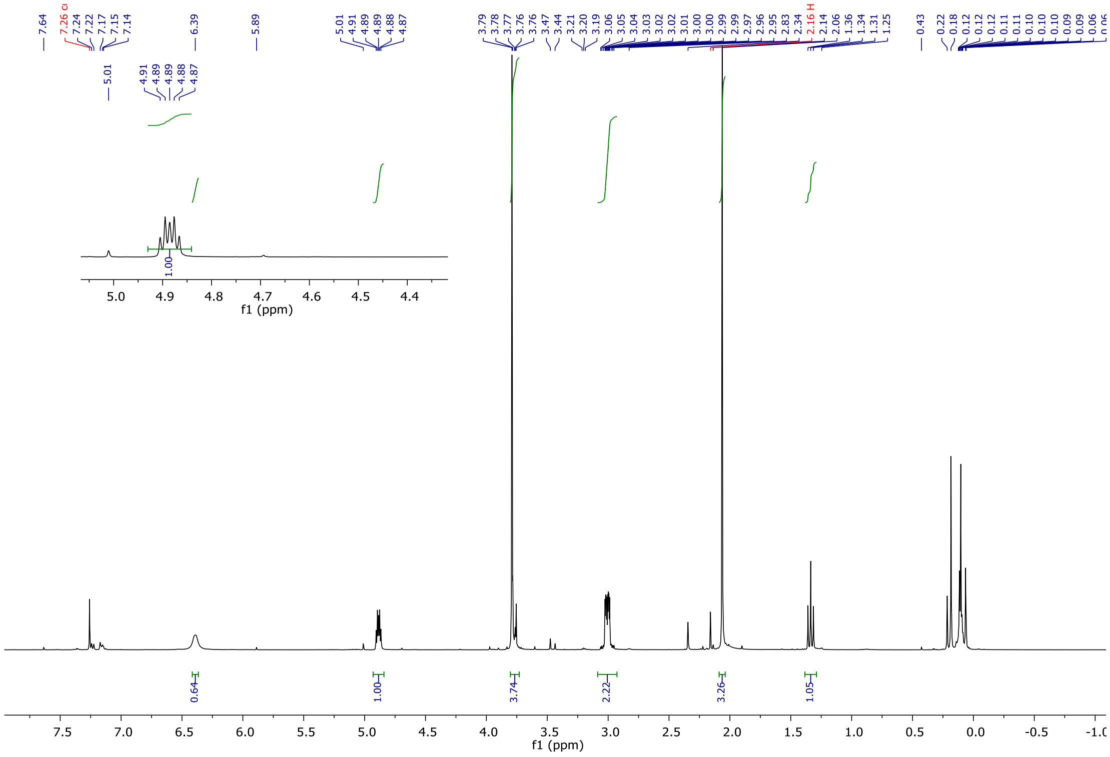

Figure *SI.7*. Crude ^{1}H-NMR of Entry 7, Table 1.

Entry 8 (No phosphite), Table 1 (RMI-VIII-024): ***1a*** (0.09 g, 0.508 mmol, 1 equiv), PhMe (10 mL, 0.05 M), TTMSS (0.31 mL, 1 mmol, 2 equiv), ACHN (0.012 g, 0.051 mmol, 0.1 equiv). Temperature 88 ^{o}C. Time 16h. Crude ^{1}H-NMR showed 0% conversion.

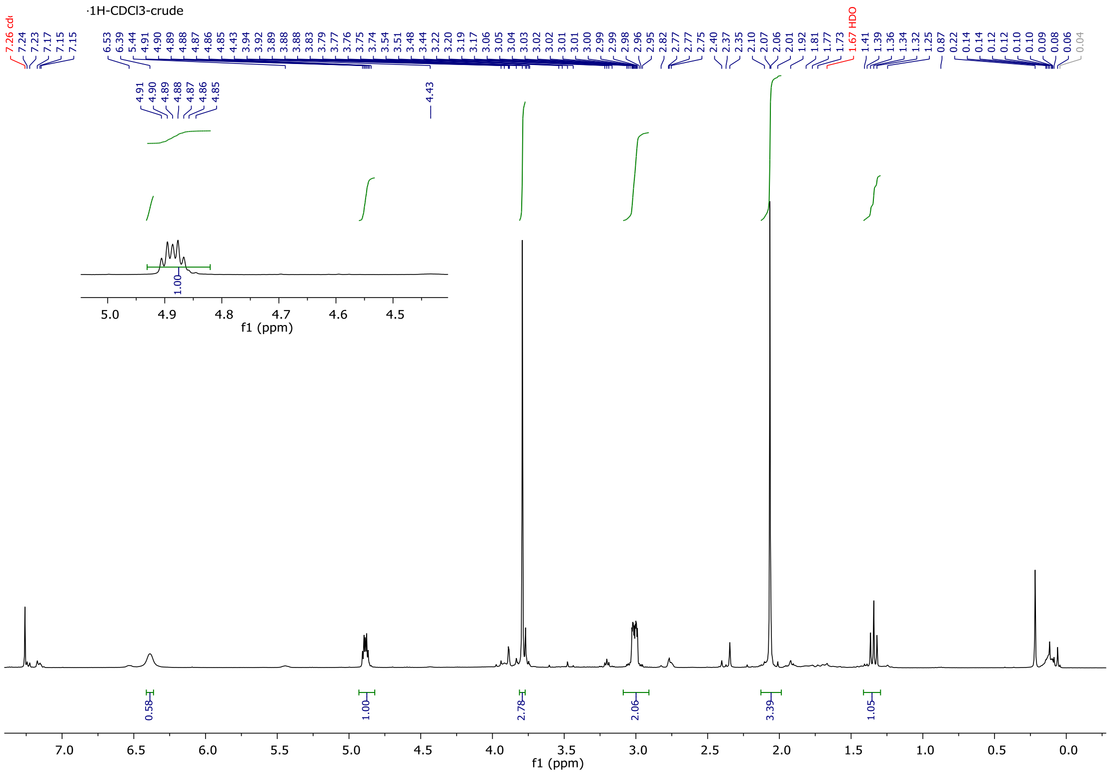

Figure *SI.8*. Crude ^{1}H-NMR of Entry 8, Table 1.

Entry 9 (No TTMSS), Table 1 (RMI-VIII-026): ***1a*** (0.09 g, 0.508 mmol, 1 equiv), PhMe (10 mL, 0.05 M), ACHN (0.012 g, 0.051 mmol, 0.1 equiv), $P(OMe)_3$ (0.012 mL, 0.102 mmol, 0.2 equiv). Temperature 88 ^{o}C. Time 16h. Crude ^{1}H-NMR showed 7% conversion.

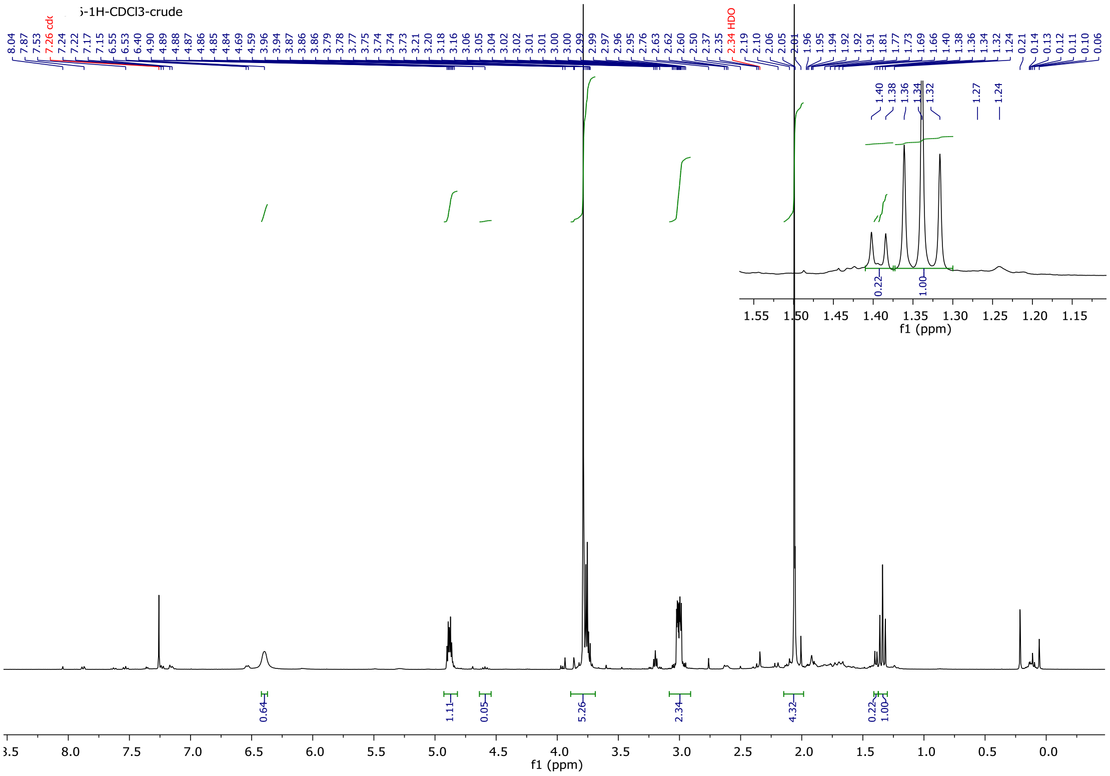

Figure *SI.9*. Crude ^{1}H-NMR of Entry 9, Table 1.

Entry 10 (No heat), Table 1 (RMI-VIII-027): ***1a*** (0.09 g, 0.508 mmol, 1 equiv), PhMe (10 mL, 0.05 M), TTMSS (0.31 mL, 1 mmol, 2 equiv), ACHN (0.012 g, 0.051 mmol, 0.1 equiv), $P(OMe)_3$ (0.012 mL, 0.102 mmol, 0.2 equiv). Temperature 88 °C. Time 16h. Crude ^1H-NMR showed 0% conversion.

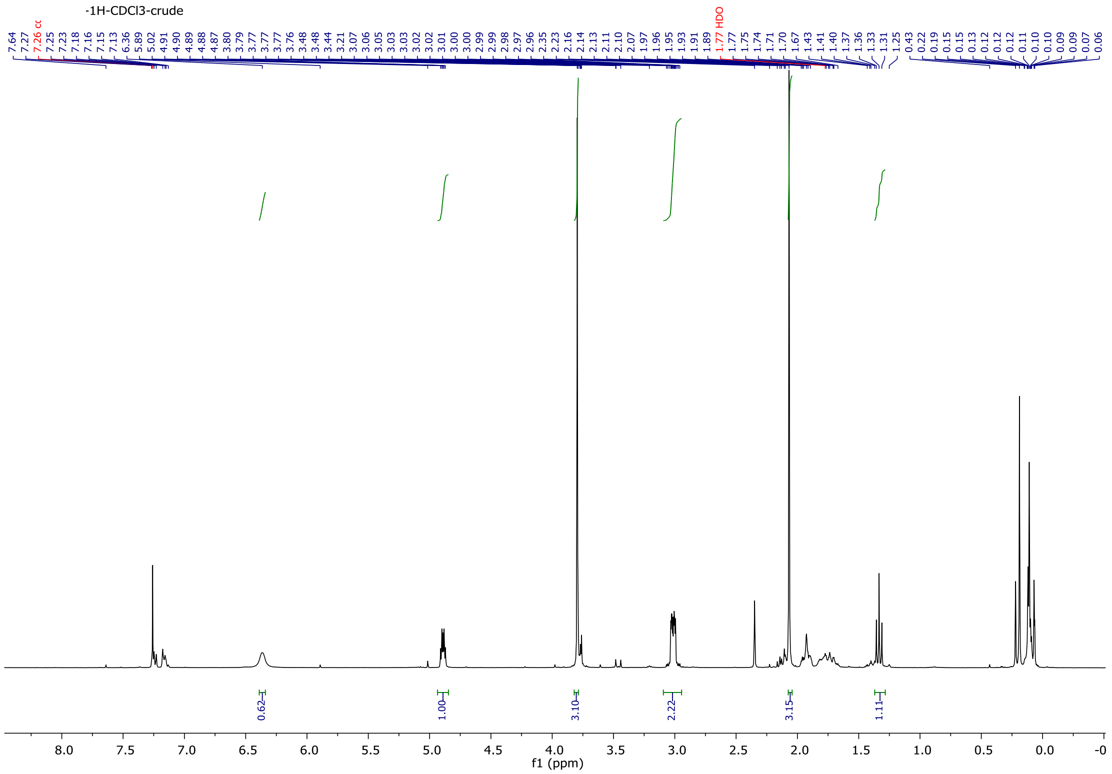

Figure *SI.10*. Crude ^1H-NMR of Entry 10, Table 1.

Table 1 (NMV-XI-097A): ***1a*** (0.05 g, 0.282 mmol, 1 equiv), PhMe (5.6 mL, 0.05 M), TTMSS (0.174 mL, 0.564 mmol, 2 equiv), ACHN (0.0069 g, 0.028 mmol, 0.1 equiv), PPh_3 (0.0147 g, 0.056 mmol, 0.2 equiv). Temperature 88 °C. Time 16 h. Crude ^1H-NMR showed > 98% conversion.

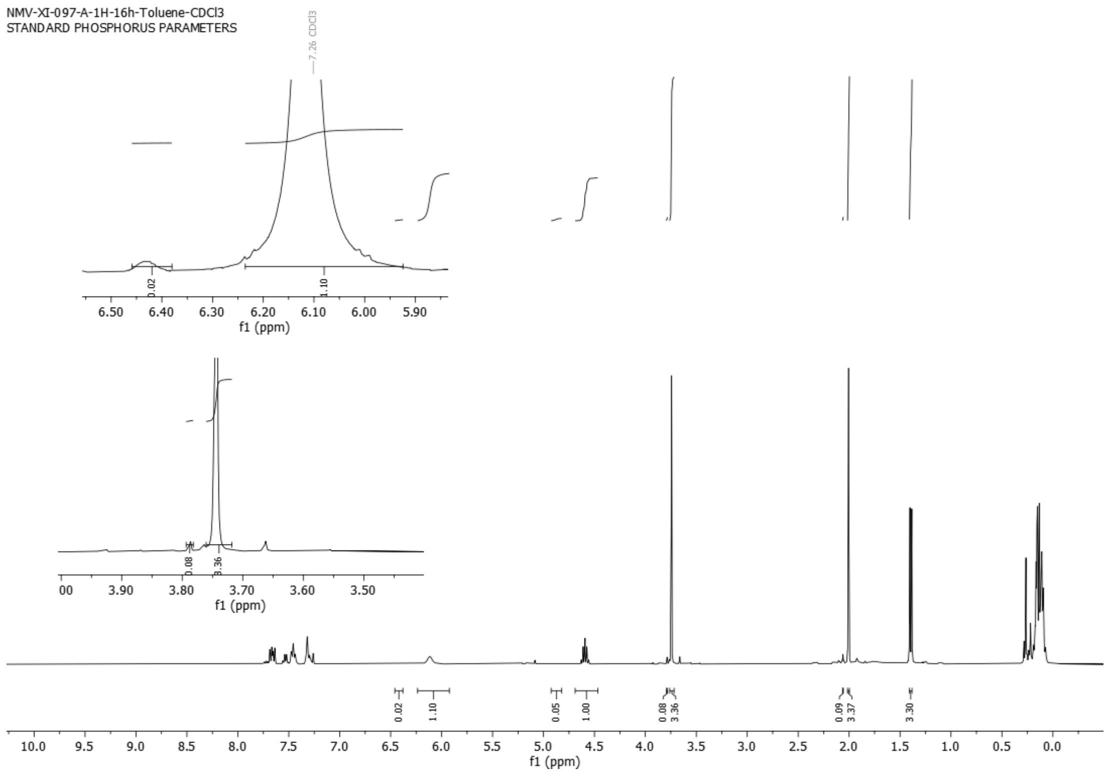

Figure *SI.11*. Crude ¹H-NMR of footnote (Toluene), Table 1. Conversion 98%

Table 1 (NMV-XI-097B): ***1a*** (0.05 g, 0.282 mmol, 1 equiv), PhMe (5.6 mL, 0.05 M), TTMSS (0.174 mL, 0.564 mmol, 2 equiv), ACHN (0.0069 g, 0.028 mmol, 0.1 equiv), PPh_3 (0.0147 g, 0.056 mmol, 0.2 equiv). Temperature 88 °C. Time 16 h. Crude ¹H-NMR showed > 92% conversion.

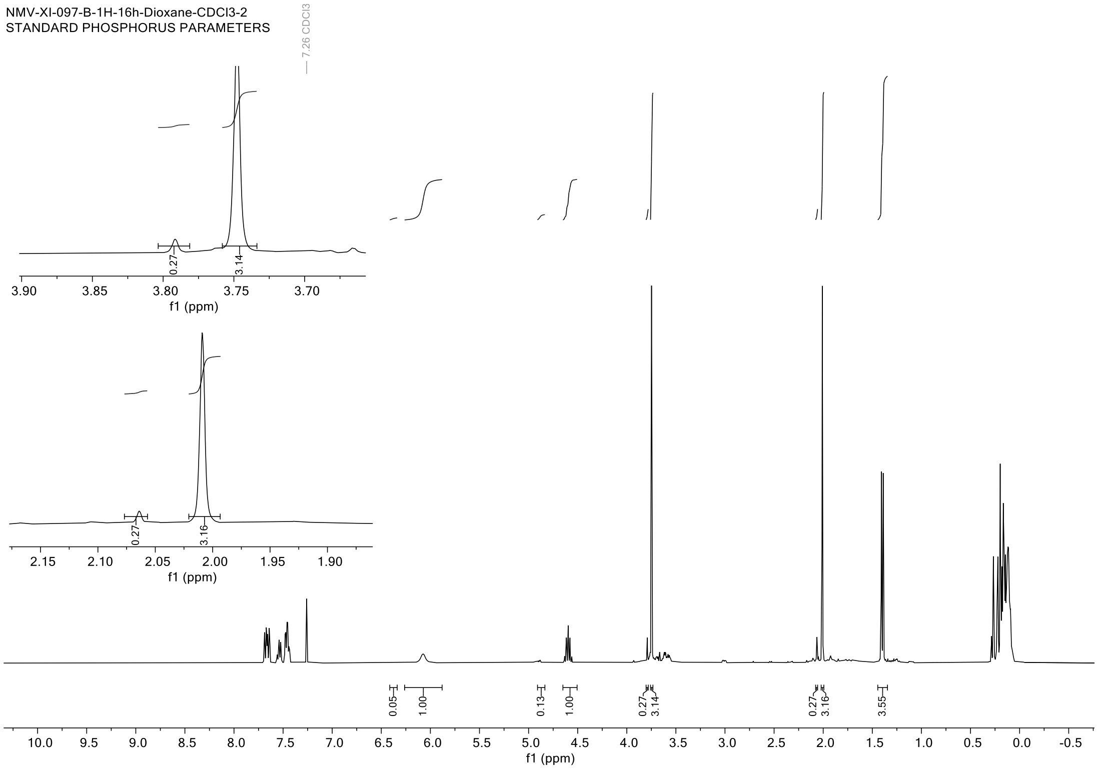

Figure *SI.12*. Crude ¹H-NMR of footnote (1,4-Dioxane), Table 1. Conversion 92%

Experimental and spectroscopic data of Table 2.

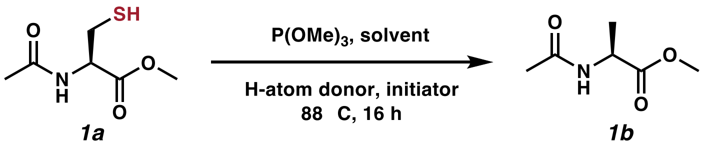

Entry 1, Table 2 (RMI-VIII-055): ***1a*** (0.09 g, 0.508 mmol, 1 equiv), MeCN/H_2O 1:1 (10 mL, 0.05 M), TTMSS (0.31 mL, 1 mmol, 2 equiv), VA-044 (0.016 g, 0.051 mmol, 0.1 equiv), $P(OMe)_3$ (0.012 mL, 0.102 mmol, 0.2 equiv). Crude ^{1}H-NMR showed 44% conversion.

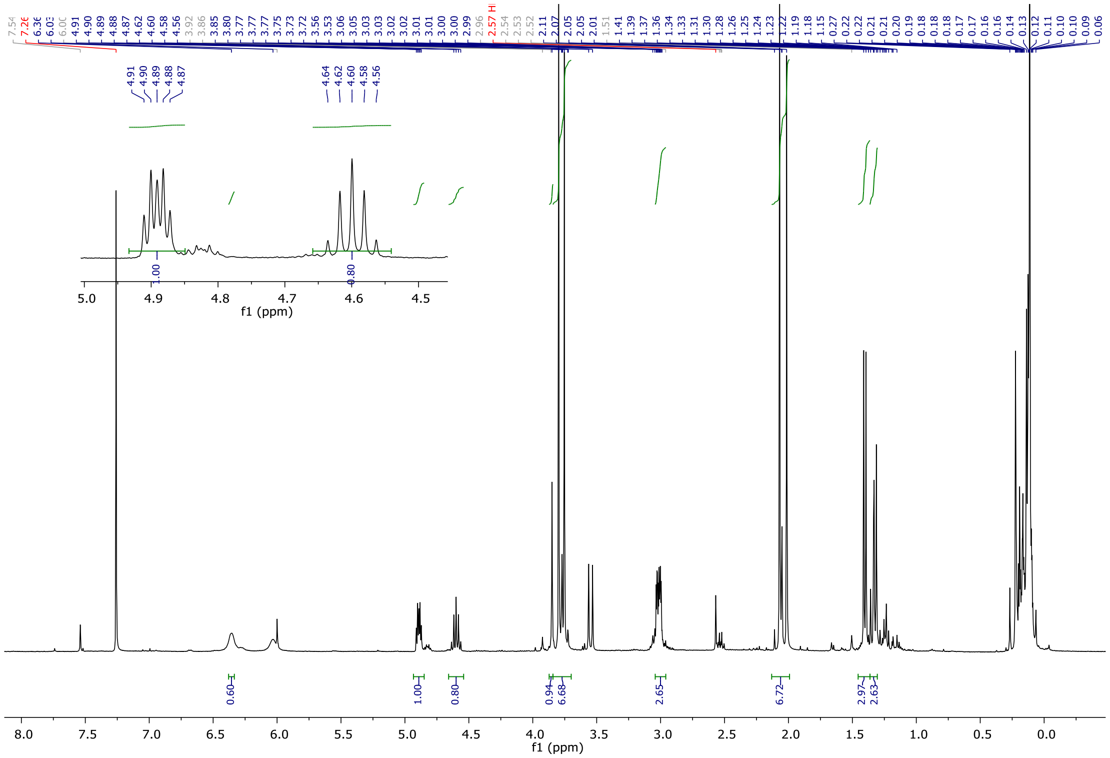

Figure *SI.13*. Crude ^{1}H-NMR of Entry 1, Table 2.

Entry 2, Table 2 (RMI-X-044): ***1a*** (0.09 g, 0.508 mmol, 1 equiv), PhMe (10 mL, 0.05 M), TTMSS (0.31 mL, 1 mmol, 2 equiv), Luperox A-98 (0.012 g, 0.051 mmol, 0.1 equiv), $P(OMe)_3$ (0.012 mL, 0.102 mmol, 0.2 equiv). Crude ^{1}H-NMR showed 22% conversion.

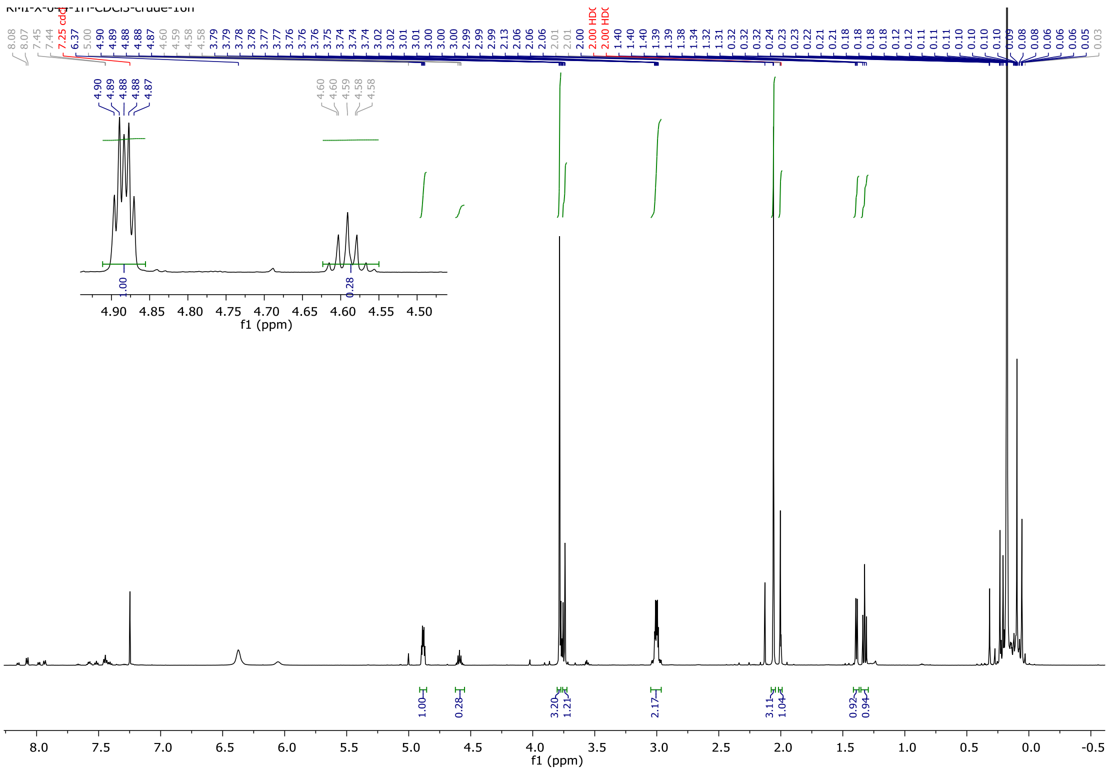

Figure *SI.14.* Crude ^{1}H-NMR of Entry 2, Table 2.

Entry 3, Table 2 (RMI-VIII-057): ***1a*** (0.09 g, 0.508 mmol, 1 equiv), PhMe (10 mL, 0.05 M), TTMSS (0.31 mL, 1 mmol, 2 equiv), dicumyl peroxide (0.014 g, 0.051 mmol, 0.1 equiv), $P(OMe)_3$ (0.012 mL, 0.102 mmol, 0.2 equiv). Crude ^{1}H-NMR showed 65% conversion.

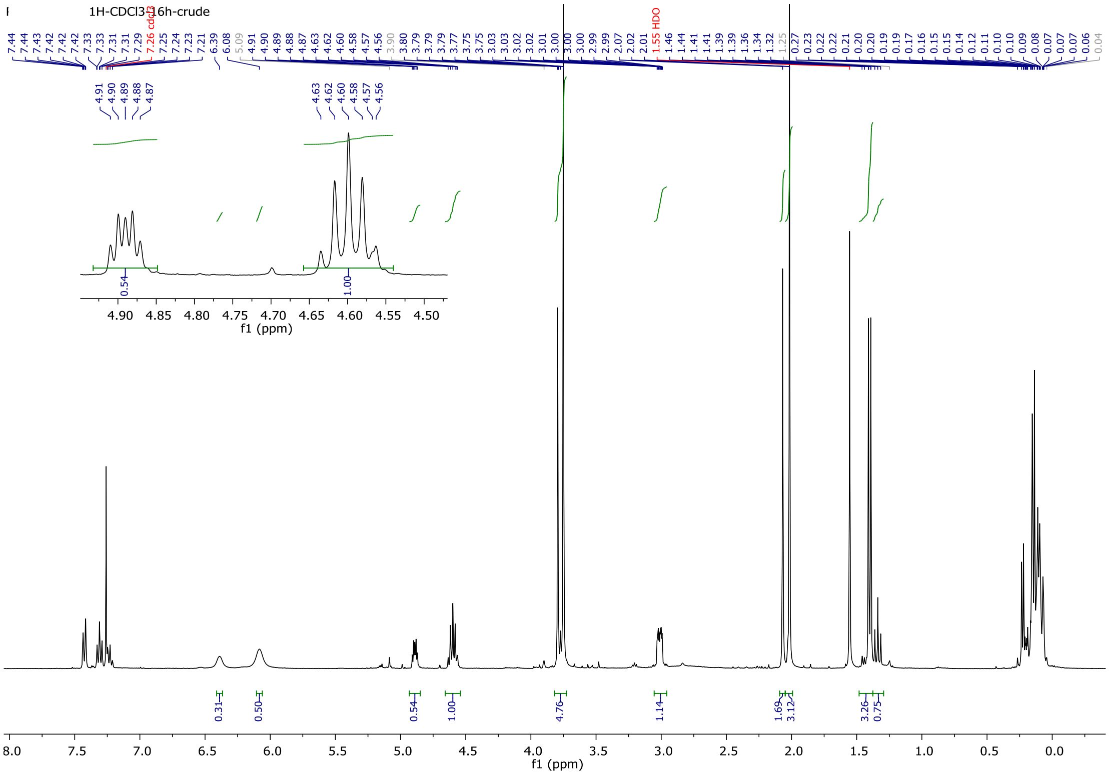

Figure *SI.15*. Crude ^{1}H-NMR of Entry 3, Table 2.

Entry 4, Table 2 (RMI-VIII-058): ***1a*** (0.09 g, 0.508 mmol, 1 equiv), PhMe (10 mL, 0.05 M), TIPS (0.21 mL, 1 mmol, 2 equiv), ACHN (0.012 g, 0.051 mmol, 0.1 equiv), $P(OMe)_3$ (0.012 mL, 0.102 mmol, 0.2 equiv). Crude ^{1}H-NMR showed 7% conversion.

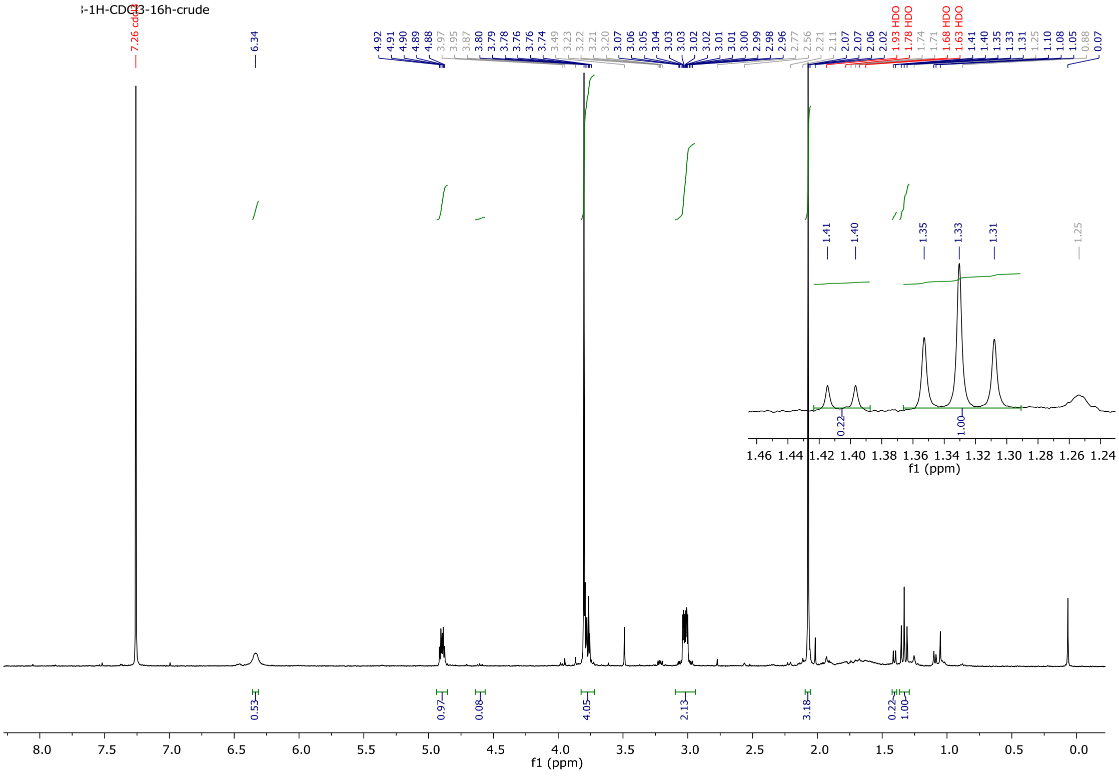

Figure *SI.16*. Crude ^{1}H-NMR of Entry 4, Table 2.

Entry 5, Table 2 (RMI-XI-057): ***1a*** (0.05 g, 0.282 mmol, 1 equiv), PhMe (5.6 mL, 0.05 M), Et_3GeH (0.09 mL, 0.564 mmol, 2 equiv), ACHN (0.0069 g, 0.028 mmol, 0.1 equiv), $P(OMe)_3$ (0.0066 mL, 0.056 mmol, 0.2 equiv). Crude ^{1}H-NMR showed 43% conversion.

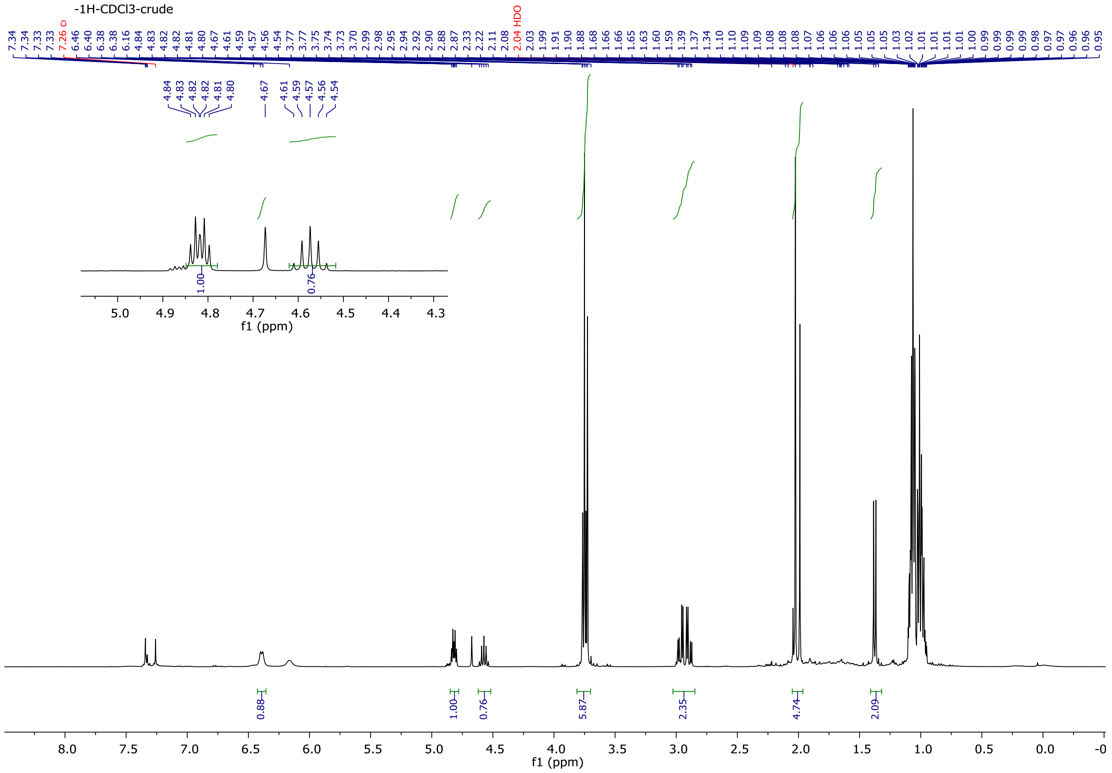

Figure *SI.17*. Crude ^{1}H-NMR of Entry 5, Table 2.

Entry 6, Table 2 (RMI-VIII-059): ***1a*** (0.09 g, 0.508 mmol, 1 equiv), PhMe (10 mL, 0.05 M), $^nBu_3SnH$ (0.27 mL, 1 mmol, 2 equiv), ACHN (0.012 g, 0.051 mmol, 0.1 equiv), $P(OMe)_3$ (0.012 mL, 0.102 mmol, 0.2 equiv). Crude ^{1}H-NMR showed 63% conversion.

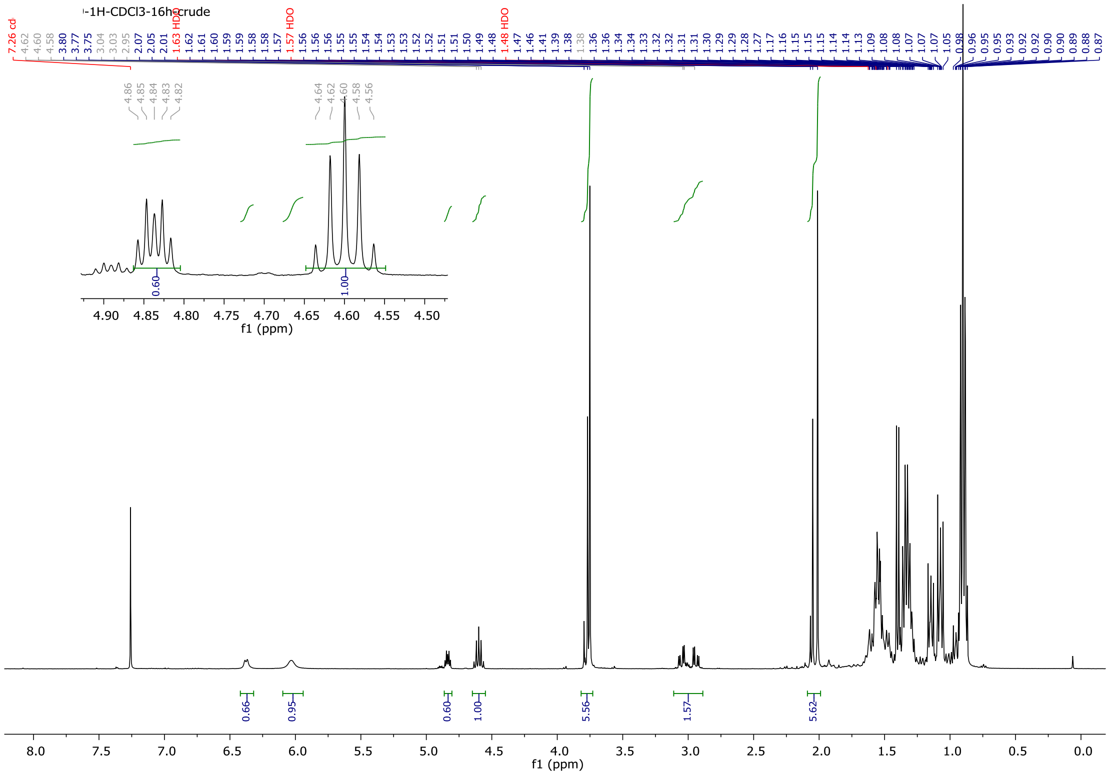

Figure *SI.18*. Crude ^{1}H-NMR of Entry 6, Table 2.

Entry 7, Table 2 (RMI-VIII-054): ***1a*** (0.09 g, 0.508 mmol, 1 equiv), MeCN (10 mL, 0.05 M), TTMSS (0.31 mL, 1 mmol, 2 equiv), ACHN (0.012 g, 0.051 mmol, 0.1 equiv), $P(OMe)_3$ (0.012 mL, 0.102 mmol, 0.2 equiv). Crude ^{1}H-NMR showed 62% conversion.

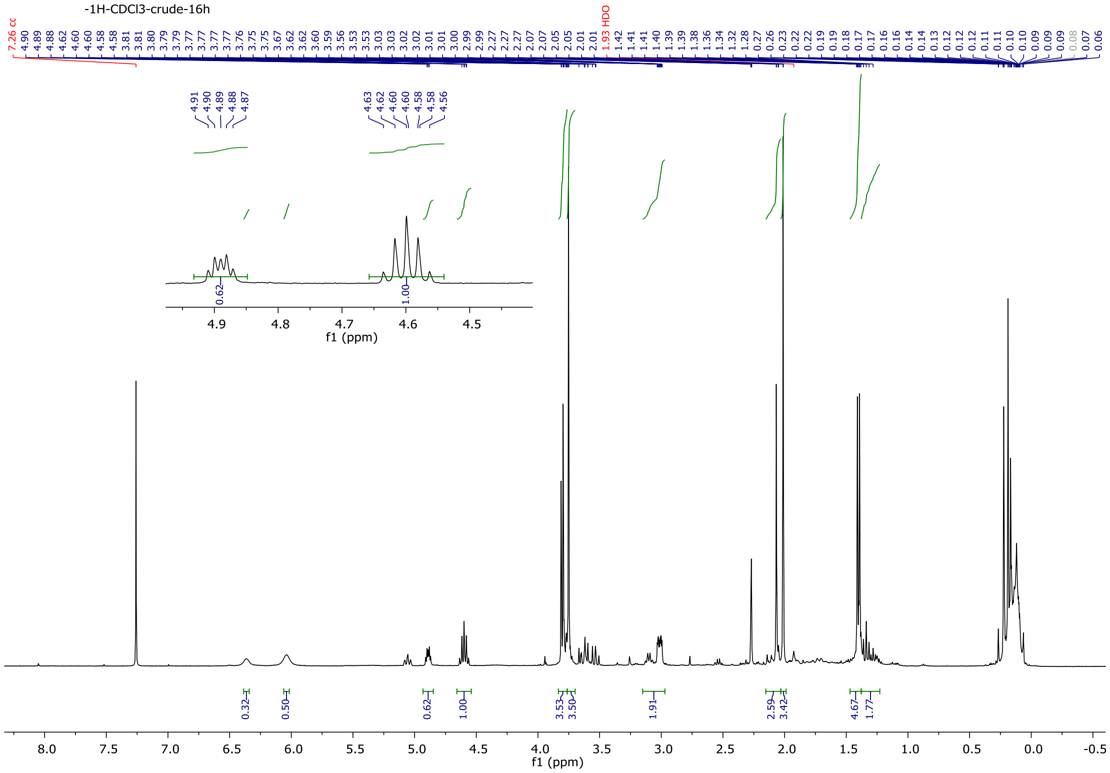

Figure *SI.19*. Crude ^{1}H-NMR of Entry 7, Table 2.

Entry 8, Table 2 (RMI-VIII-053): ***1a*** (0.09 g, 0.508 mmol, 1 equiv), THF (10 mL, 0.05 M), TTMSS (0.31 mL, 1 mmol, 2 equiv), ACHN (0.012 g, 0.051 mmol, 0.1 equiv), $P(OMe)_3$ (0.012 mL, 0.102 mmol, 0.2 equiv). Crude ^{1}H-NMR showed 82% conversion.

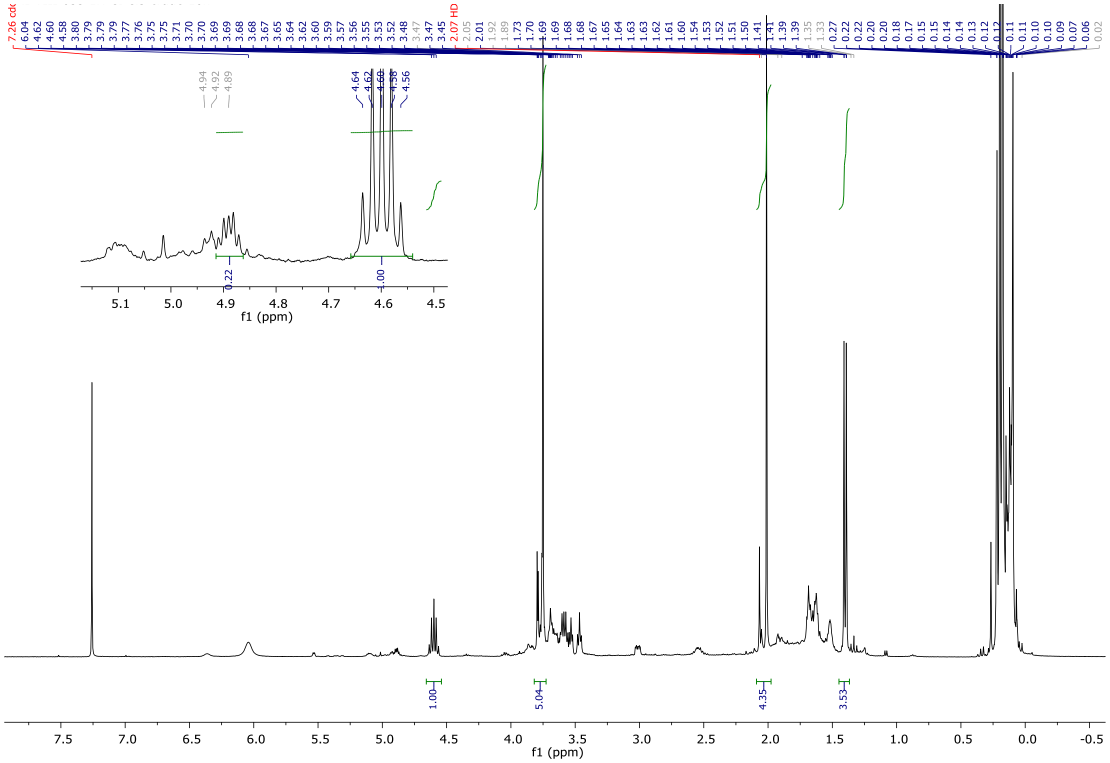

Figure *SI.20*. Crude ^{1}H-NMR of Entry 8, Table 2.

Entry 9, Table 2 (RMI-VIII-052): ***1a*** (0.09 g, 0.508 mmol, 1 equiv), 1,4-dioxane (10 mL, 0.05 M), TTMSS (0.31 mL, 1 mmol, 2 equiv), ACHN (0.012 g, 0.051 mmol, 0.1 equiv), $P(OMe)_3$ (0.012 mL, 0.102 mmol, 0.2 equiv). Crude ^{1}H-NMR showed > 99% conversion.

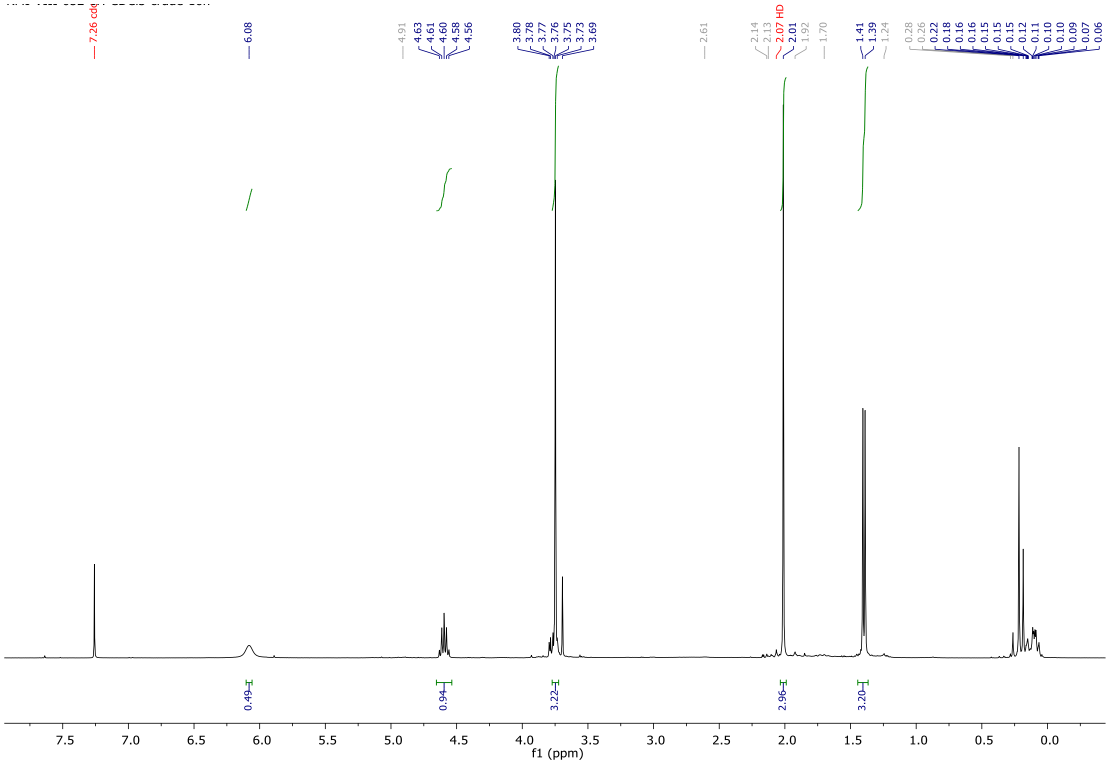

Figure *SI.21*. Crude ^{1}H-NMR of Entry 9, Table 2.

Experimental and spectroscopic data of Table 3.

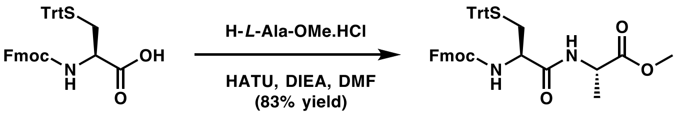

SI-1 ***SI-2***

Fmoc-*L*-Cys(Trt)-*L*-Ala-OMe (*SI-2*, GS-VIII-057). To a stirred solution of Fmoc-*L*-Cys(Trt)-OH ***SI-1*** (2.343 g, 4 mmol, 1 equiv) in dry DMF (20 mL, 0.2 M) was added HATU (1.673 g, 4.4 mmol, 1.1 equiv), DIEA (2.1 mL, 12 mmol, 3 equiv) and H-*L*-Ala-OMe.HCl (0.586 g, 4.2 mmol, 1.05 equiv) under an atmosphere of argon. The reaction mixture was stirred for 3h at room temperature. The reaction mixture was quenched with water and extracted with EtOAc. Combined organic layers were dried over Na_2SO_4, filtered, and concentrated under vacuum. Crude product was purified by flash column chromatography eluting with 25% EtOAc/Hexane to afford ***SI-2*** as white solid (2.23 g, 83% yield). ^{1}H NMR (499 MHz, Chloroform-*d*) δ 7.75 (t, *J* = 8.4 Hz, 2H), 7.57 (d, *J* = 7.5 Hz, 2H), 7.46 – 7.34 (m, 9H), 7.32 – 7.25 (m, 9H), 7.25 – 7.18 (m, 3H), 6.35 (dd, *J* = 7.6, 2.9 Hz, 1H), 5.05 (s, 1H), 4.48 (h, *J* = 7.2, 6.8 Hz, 1H), 4.38 (dd, *J* = 7.0, 2.5 Hz, 2H), 4.20 (t, *J* = 6.9 Hz, 1H), 3.81 – 3.73 (m, 1H), 3.69 (s, 3H), 2.72 (dd, *J* = 13.3, 7.8 Hz, 1H), 2.62 (dd, *J* = 13.4, 5.2 Hz, 1H), 1.35 (d, *J* = 7.2 Hz, 3H). ^{13}C NMR (126 MHz, Chloroform-*d*) δ 172.70, 169.57, 144.34, 143.74, 143.65, 141.28, 129.59, 128.09, 127.96, 127.74, 127.73, 127.08, 126.92, 125.05, 125.04, 119.98, 119.97, 67.35, 52.43, 48.18, 47.07, 33.90, 18.24. HRMS $[M+H]^+$ calc'd for $[C_{41}H_{38}N_2O_5S+H]$: *m/z* 671.2574, found 671.2566.

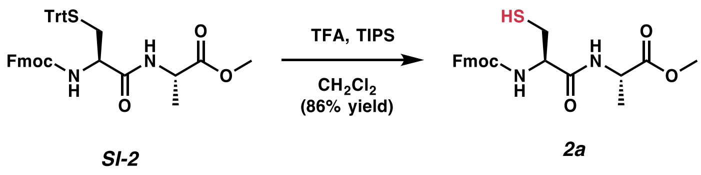

Fmoc-*L*-Cys-*L*-Ala-OMe (*2a*, GS-VIII-058). To a stirred solution of ***SI-2*** (0.671 g, 1 mmol, 1 equiv) in dry CH_2Cl_2 (15 mL, 0.07 M) was added TIPS (1.022 mL, 5 mmol, 5 equiv) followed by dropwise addition of TFA (0.765 mL, 10 mmol, 10 equiv) at room temperature. The reaction mixture was stirred for 45 mins. Reaction mixture was concentrated under vacuum and cold diethyl ether (20 mL) was added to get solid product. The solid was filtered, washed with cold ether (2 X 10 mL) and dried to afford ***2a*** as white solid (0.368 g, 86% yield). ^{1}H NMR (499 MHz, Chloroform-*d*) δ 7.77 (d, *J* = 7.6 Hz, 2H), 7.59 (d, *J* = 7.5 Hz, 2H), 7.41 (t, *J* = 7.5 Hz, 2H), 7.32 (tdd, *J* = 7.5, 2.2, 1.2 Hz, 2H), 6.72 (s, 1H), 5.72 (s, 1H), 4.57 (p, *J* = 7.2 Hz, 1H), 4.53 – 4.45 (m, 1H), 4.41 (d, *J* = 10.3 Hz, 2H), 4.23 (t, *J* = 6.8 Hz, 1H), 3.75 (s, 3H), 3.03 (d, *J* = 17.8 Hz, 1H), 2.75 (s, 1H), 1.43 (d, *J* = 7.2 Hz, 3H). ^{13}C NMR (126 MHz, Chloroform-*d*) δ 172.86, 143.67, 141.33, 141.31, 127.79, 127.09, 124.95, 120.04, 120.03, 67.22, 52.61, 48.37, 47.13, 27.08, 18.14. HRMS [M+H]$^+$ calc'd for [$C_{22}H_{24}N_2O_5S$+H]: *m/z* 429.1479, found 429.1482.

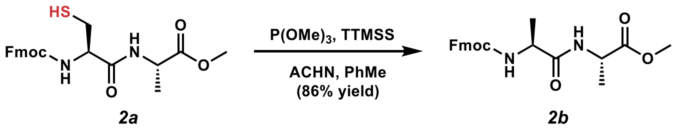

Fmoc-*L*-Ala-*L*-Ala-OMe (*2b*, GS-VIII-060). To a 20-mL microwave vial with 14/20 septum under an atmosphere of argon, was added ***2a*** (0.11 mL, 0.25 mmol, 1 equiv), PhMe (10 mL, 0.025 M), TTMSS (154 mL, 0.5 mmol, 2 equiv). The reaction mixture was purged with argon for few minutes then P(OMe)$_3$ (0.006 mL, 0.05 mmol, 0.2 equiv) and ACHN (0.006 g, 0.025 mmol, 0.1 equiv) were added. The reaction vial was sealed and placed in pre-heated oil bath at 88 °C and stirred for 20 h. The reaction mixture was cooled to room temperature and concentrated under vacuum. Residue was purified using silica gel flash column chromatography using 10% EtOAc/petroleum ether as an eluent to afford ***2b*** as white solid (0.088 g, 86% yield). ^{1}H NMR (600 MHz, Chloroform-*d*) δ 7.75 (d, *J* = 7.5 Hz, 2H), 7.57 (dd, *J* = 7.6, 3.1 Hz, 2H), 7.38 (t, *J* = 7.5 Hz, 2H), 7.30 (t, *J* = 7.4 Hz, 2H), 6.56 (d, *J* = 7.3 Hz, 1H), 5.44 (d, *J* = 7.9 Hz, 1H), 4.56 (p, *J* = 7.3 Hz, 1H), 4.38 (d, *J* = 7.1 Hz, 2H), 4.27 (h, *J* = 6.5 Hz, 1H), 4.20 (t, *J* = 7.1 Hz, 1H), 3.73 (s, 3H), 1.40 (t, *J* = 6.2 Hz, 6H). ^{13}C NMR (151 MHz, Chloroform-*d*) δ 173.09, 171.74, 143.77, 143.72, 141.27, 141.26, 127.70, 127.04, 125.03, 119.97, 119.96, 67.08, 52.49, 48.10, 47.10, 18.77, 18.28, 14.17. HRMS [M+H]$^+$ calc'd for [$C_{22}H_{24}N_2O_5$+H]: *m/z* 397.1758, found 397.1759. *These data are consistent with previously reported data.*[1]

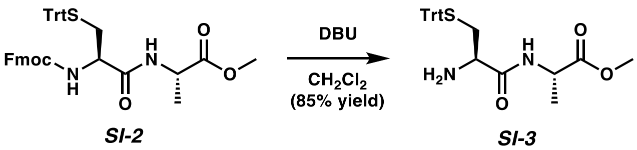

H-*L*-Cys(Trt)-*L*-Ala-OMe (*SI-3*, GS-VIII-062). To a stirred solution of ***SI-2*** (0.154 g, 2.3 mmol, 1 equiv) in CH_2Cl_2 (15 mL, 0.15 M), was added DBU (0.41 mL, 2.76 mmol, 1.2 equiv) under N_2-balloon atmosphere. Reaction mixture was stirred for 1 h and quenched with water (~20 mL). Reaction mixture was extracted with CH_2Cl_2 (3 X 25 mL) and combined organic layers were dried over anhydrous Na_2SO_4, filtered and concentrated under vacuum. The crude product was purified using silica gel with flash column chromatography using 20% EtOAc/Hexanes as an eluent to afford ***SI-3*** as yellow foam solid (0.766 g, 74% yield). ^{1}H NMR (499 MHz, Chloroform-*d*) δ 7.51 (d, *J* = 7.8 Hz, 1H), 7.48 – 7.39 (m, 6H), 7.32 – 7.25 (m, 7H), 7.25 – 7.15 (m, 3H), 4.48 (p, *J* = 7.3 Hz, 1H), 3.70 (s, 3H), 3.01 (dd, *J* = 8.5, 3.9 Hz, 1H), 2.71 (dd, *J* = 12.8, 3.9 Hz, 1H), 2.58 (dd, *J* = 12.8, 8.5 Hz, 1H), 1.35 (d, *J* = 7.2 Hz, 3H). ^{13}C NMR (126 MHz, Chloroform-*d*) δ 173.22, 172.52, 144.56, 129.59, 128.16, 127.98, 127.04, 126.81, 77.27, 66.99, 53.84, 52.34, 52.33, 47.72, 37.21, 18.31. HRMS [M+H]$^+$ calc'd for [$C_{26}H_{28}N_3O_2S$+H]$^+$: *m/z* 449.1893, found 449.1888.

[1] Kamiński, Z. J.; Kolesińska, B.; Kolesińska, J.; Sabatino, G.; Chelli, M.; Rovero, P.; Błaszczyk, M.; Główka, M. L.; Papini, A. M. *N*-Triazinylammonium tetrafluoroborates. A new generation of efficient coupling reagents useful for peptide synthesis. *J. Am. Chem. Soc.* **2005**, *127*, 16912-16920. https://doi.org/10.1021/ja054260y

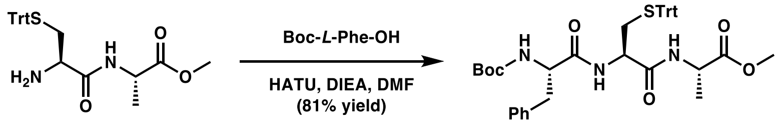

Boc-*L*-Phe-*L*-Cys(Trt)-*L*-Ala-OMe (*SI-4*, GS-VIII-063). To a stirred solution of Boc-*L*-Phe-OH (0.265 g, 1 mmol, 1 equiv) in dry DMF (10 mL, 0.1 M) under an atmosphere of argon, was added HATU (0.418 g, 1.1 mmol, 1.1 equiv), DIEA (0.35 mL, 2 mmol, 2 equiv) followed by ***SI-3*** (0.449 g, 1 mmol, 1 equiv). The reaction mixture was stirred for 3h at room temperature. Crushed Ice was added to precipitate the solids of the reaction mixture and the obtained solids were filtered through sintered funnel. The obtained crude solid was washed with hexanes then purified with silica gel using flash column chromatography using 2% MeOH in CH_2Cl_2 to afford ***SI-4*** as white solid (0.56 g, 81% yield). ^{1}H NMR (499 MHz, Chloroform-*d*) δ 7.43 – 7.36 (m, 6H), 7.33 – 7.27 (m, 7H), 7.27 – 7.16 (m, 8H), 7.16 – 7.11 (m, 2H), 6.62 (s, 1H), 6.18 (d, *J* = 7.0 Hz, 1H), 4.92 (s, 1H), 4.45 (h, *J* = 7.4 Hz, 1H), 4.16 (q, *J* = 7.2 Hz, 1H), 3.93 (s, 1H), 3.68 (s, 3H), 3.04 (dd, *J* = 14.0, 6.6 Hz, 1H), 2.96 (dd, *J* = 14.2, 5.6 Hz, 1H), 2.63 (d, *J* = 17.6 Hz, 1H), 2.47 (dd, *J* = 13.0, 6.0 Hz, 1H), 1.35 (d, *J* = 5.1 Hz, 12H). ^{13}C NMR (101 MHz, Chloroform-*d*) δ 172.58, 171.05, 169.19, 144.34, 136.37, 129.57, 129.20, 128.69, 128.03, 127.91, 127.89, 126.99, 126.87, 81.10, 77.32, 77.01, 76.69, 67.17, 52.32, 52.18, 48.22, 33.17, 28.19. HRMS [M+H]$^+$ calc'd for [$C_{40}H_{45}N_3O_6S$ +H]: *m/z* 696.3102, found 696.3096.

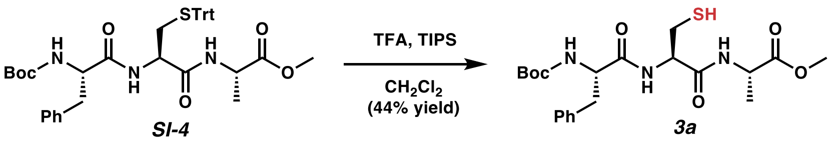

Boc-*L*-Phe-*L*-Cys-*L*-Ala-OMe (*3a*, GS-VIII-064). To a stirred solution of ***SI-4*** (0.564 g, 0.81 mmol, 1 equiv) in dry CH_2Cl_2 (15 mL, 0.54 M), was added TIPS (0.92 mL, 4.5 mmol, 5 equiv) followed by dropwise addition of TFA (0.5 mL, 6.48 mmol, 8 eq) at 0 °C. The reaction mixture was stirred at room temperature for 1.5 h. Reaction mixture was concentrated to dryness and diethyl ether (20 mL) was added. The formed solid was filtered and washed with ether (2 X 10 mL) and purified using silica gel with flash column chromatography using 1% MeOH in CH_2Cl_2 as eluent to afford ***3a*** as white solid (0.163 g, 44% yield). ^{1}H NMR (499 MHz, Chloroform-*d*) δ 7.35 – 7.22 (m, 4H), 7.22 – 7.17 (m, 2H), 7.11 (s, 1H), 6.61 (d, *J* = 8.2 Hz, 1H), 5.15 (d, *J* = 6.8 Hz, 1H), 4.64 (ddd, *J* = 9.3, 5.7, 3.8 Hz, 1H), 4.49 (p, *J* = 7.2 Hz, 1H), 4.24 (q, *J* = 7.3 Hz, 1H), 3.71 (s, 3H), 3.04 (dt, *J* = 12.7, 7.2 Hz, 3H), 2.30 (s, 1H), 1.46 – 1.39 (m, 12H). ^{13}C NMR (126 MHz, Chloroform-*d*) δ 172.69, 171.38, 169.04, 136.22, 129.17, 128.85, 127.27, 80.59, 52.43, 48.42, 38.05, 28.22, 26.32, 17.62. HRMS [M+H]$^+$ calc'd for [$C_{21}H_{31}N_3O_6S$ +H]: *m/z* 454.2006, found 454.2007.

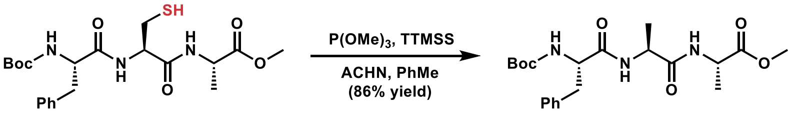

Boc-*L*-Phe-*L*-Ala-*L*-Ala-OMe (*3b*, GS-VIII-065). To a 20-mL microwave vial with 14/20 septum under an atmosphere of argon, was added ***3a*** (0.091 g, 0.2 mmol, 1 equiv), PhMe (10 mL, 0.02 M), TTMSS (0.123 mL, 0.4 mmol, 2 equiv). The reaction mixture was purged with argon for few minutes then $P(OMe)_3$ (0.0047 mL, 0.04 mmol, 0.2 equiv) and ACHN (0.0049 g, 0.02 mmol, 0.1 equiv) were added. The reaction vial was sealed and placed in pre-heated oil bath at 88 ºC and stirred for 12 h. The reaction mixture was cooled to room temperature and concentrated under vacuum. The crude was purified using silica gel with flash column chromatography using 10% EtOAc in petroleum ether as an eluent to afford ***3b*** as white solid (0.072 g, 86% yield). ^{1}H NMR (499 MHz, Chloroform-*d*) δ 7.26 (t, *J* = 7.2 Hz, 3H), 7.25 – 7.13 (m, 4H), 7.10 (d, *J* = 7.5 Hz, 1H), 6.60 (d, *J* = 7.8 Hz, 1H), 5.31 (d, *J* = 7.9 Hz, 1H), 4.50 (p, *J* = 7.2 Hz, 2H), 4.37 – 4.32 (m, 1H), 3.71 (s, 3H), 3.02 (d, *J* = 7.3 Hz, 2H), 1.37 (d, *J* = 3.5 Hz, 15H). ^{13}C NMR (126 MHz, Chloroform-*d*) δ 173.03, 171.58, 171.15, 136.53, 129.24, 128.75, 128.60, 126.95, 77.23, 52.39, 48.53, 48.07, 28.22, 17.93, -0.59, -0.76. HRMS [M+H]$^+$ calc'd for [$C_{21}H_{31}N_3O_6$+H]: *m/z* 422.2286, found 422.2290.

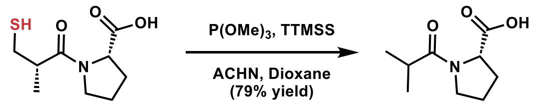

4a *4b*

Isobutyryl-*L*-proline (*4b*, NMV-XI-025): To a 20-mL microwave vial (with 14/20 septum under an atmosphere of argon), ***4a*** (0.0435 g, 0.2 mmol, 1 equiv), dioxane (4 mL, 0.05 M), and TTMSS (0.123 mL, 0.4 mmol, 2 equiv) were added. The reaction mixture was purged with argon for few minutes then $P(OMe)_3$ (0.0047 mL, 0.04 mmol, 0.2 equiv) and ACHN (0.0049 g, 0.02 mmol, 0.1 equiv) were added. The reaction vial was sealed and placed in pre-heated silicon oil bath at 88 °C and stirred for 48 h. The reaction mixture was cooled to room temperature and concentrated under vacuum. The crude was purified using reversed phase semi prep HPLC to afford ***4b*** as white solid. (0.029 g, 79% yield). Mixture of rotamers. ^{1}H NMR (400 MHz, Methanol-*d*$_4$) δ 4.59 (dd, *J* = 8.6, 2.6 Hz, 0H), 4.41 (dd, *J* = 9.0, 3.7 Hz, 1H), 3.74 – 3.62 (m, 1H), 3.58 (td, *J* = 7.9, 4.1 Hz, 0H), 3.48 (ddd, *J* = 11.8, 8.7, 7.5 Hz, 0H), 2.81 (hept, *J* = 6.8 Hz, 1H), 2.56 (p, *J* = 6.7 Hz, 0H), 2.44 – 2.16 (m, 1H), 2.15 – 1.80 (m, 3H), 1.17 – 1.03 (m, 6H). ^{13}C NMR (101 MHz, Methanol-*d*$_4$) δ 177.66, 176.95, 174.35, 174.24, 59.39, 58.82, 46.21, 32.40, 31.91, 30.84, 28.82, 24.31, 22.02, 18.45, 17.79, 17.66, 17.60. *These data are consistent with previously reported data.*[2]

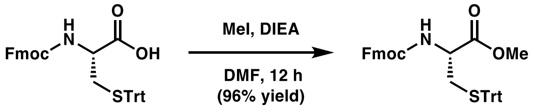

SI-1 *SI-5*

Fmoc-*L*-Cys(Trt)-OMe (*SI-5*, NMV-X-077): To a solution of ***SI-1*** (1.25 g, 2.13 mmol, 1 equiv) in DMF (5 mL, 0.43 M), DIEA (0.744 mL, 4.27 mmol, 2 equiv) was added and reaction mixture was stirred for 10 minutes. MeI (0.266 mL, 4.27 mmol, 2 equiv) was added. The reaction mixture was stirred at room temperature for additional 12 h. The reaction mixture was poured onto water (10 mL) and extracted with ethyl acetate. The aqueous layer was extract again with ethyl acetate and washed with brine solution. Combined organic layers were washed with brine solution. The combined organic layers were dried over anhydrous $MgSO_4$, filtered, and concentrated under vacuum. The residue was purified with silica gel using an automated flash column chromatography system (0–90% EtOAc/Hexane) to afford ***SI-5*** as a white solid. (1.24 g, 96% yield). ^{1}H NMR (400 MHz, Chloroform-*d*) δ 7.79 (dd, *J* = 7.8, 3.5 Hz, 2H), 7.64 (dd, *J* = 7.7, 4.1 Hz, 2H), 7.48 – 7.38 (m, 8H), 7.37 – 7.19 (m, 12H), 5.31 (d, *J* = 8.2 Hz, 1H), 4.40 (dt, *J* = 11.3, 6.0 Hz, 3H), 4.26 (t, *J* = 7.2 Hz, 1H), 3.74 (s, 3H), 2.71 (d, *J* = 5.5 Hz, 2H). ^{13}C NMR (101 MHz, Chloroform-*d*) δ 170.98, 155.60, 144.30, 143.90, 143.76, 141.33, 141.31, 129.54, 128.04, 127.75, 127.74, 127.12, 127.11, 126.94, 125.17, 125.13, 120.00, 77.41, 77.29, 77.09, 76.77, 67.15, 67.09, 52.97, 52.65, 47.14, 34.05. HRMS [M+Na]$^+$ m/z calc'd for $[C_{38}H_{33}NO_4S+Na]^+$: 622.2023, found 622.2008.

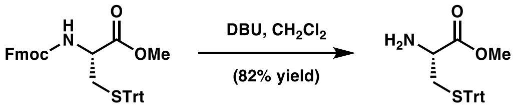

SI-5 *SI-6*

H-*L*-Cys(Trt)-OMe (*SI-6*, NMV-X-078): To a solution of ***SI-5*** (0.700 g, 1.17 mmol, 1 equiv) in CH_2Cl_2 (3.8 mL, 0.3 M), DBU (0.192 mL, 1.28 mmol, 1.1 equiv) was added and reaction mixture was stirred for 15 minutes at room temperature. The reaction mixture was concentrated under vacuum and purified with silica gel using an automated flash column chromatography system (0–10% MeOH/CH_2Cl_2) to afford ***SI-6*** as a colorless oil. (0.362 g, 82% yield). ^{1}H NMR (400 MHz, Chloroform-*d*) δ 7.46 (dd, *J* = 7.8, 1.9 Hz, 6H), 7.36 – 7.20 (m, 9H), 3.69 (s, 3H), 3.24 (dd, *J* = 7.8, 4.8 Hz, 1H), 2.63 (dd, *J* = 12.5, 4.8 Hz, 1H), 2.51 (dd, *J* = 12.5, 7.8 Hz, 1H). ^{13}C NMR (101 MHz, Chloroform-*d*) δ 174.27, 144.69, 129.73, 128.08, 126.92, 67.02, 53.95, 52.29, 37.05. HRMS [2M+H]$^+$ m/z calc'd for $[C_{46}H_{46}N_2O_4S_2+H]^+$: 755.2970, found 755.2959.

[2] Stamm, S.; Heimgartner, H. Novel N-(2, 2-Dimethyl-2H-azirin-3-yl)-l-prolinates as Aib-Pro Synthons. *Helv. Chim. Acta* **2006**, *89*, 1841-1855. https://doi.org/10.1002/hlca.200690178

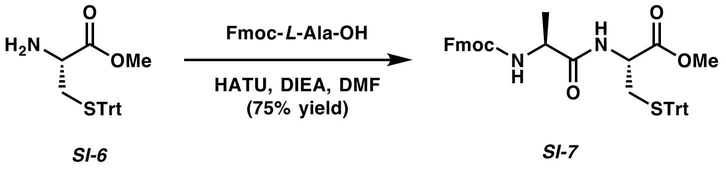

Fmoc-*L*-Ala-*L*-Cys(Trt)-OMe (*SI-7*, NMV-X-079): To a solution of Fmoc-*L*-Ala-OH (0.261 g, 0.839 mmol, 1.1 equiv) in DMF (3.3 mL, 0.25 M) was added HATU (0.319 g, 0.839 mmol, 1.1 equiv) then DIEA (0.217 mL,1.678 mmol, 2.2 equiv). ***SI-6*** (0.288 g, 0.763 mmol, 1 equiv) was added as a solution in DMF (3 mL, 0.25 M). Reaction mixture was stirred for 2 h. Water was added, and the reaction mixture was extracted with ethyl acetate (2 x 100 mL). Combined organic layers were washed with brine solution (2 X 100 mL). The combined organic layers were dried over anhydrous $MgSO_4$, filtered, and concentrated under vacuum. The residue was purified with silica gel using an automated flash column chromatography system (0–12% Metanol/CH_2Cl_2) to afford ***SI-7*** as a white solid. (0.384 g, 75% yield). ^{1}H NMR (400 MHz, Chloroform-*d*) δ 7.77 (d, *J* = 7.5 Hz, 2H), 7.58 (t, *J* = 6.8 Hz, 2H), 7.44 – 7.34 (m, 8H), 7.28 (dt, *J* = 14.9, 7.5 Hz, 8H), 7.19 (dd, *J* = 8.2, 6.2 Hz, 3H), 6.32 (d, *J* = 7.8 Hz, 1H), 5.42 (d, *J* = 7.5 Hz, 1H), 4.54 (dt, *J* = 7.5, 5.2 Hz, 1H), 4.40 (d, *J* = 7.2 Hz, 2H), 4.23 (q, *J* = 8.2, 7.0 Hz, 2H), 3.70 (s, 3H), 2.79 – 2.57 (m, 2H), 1.38 (d, *J* = 7.0 Hz, 3H). ^{13}C NMR (101 MHz, Chloroform-*d*) δ 171.95, 170.61, 155.90, 144.35, 144.04, 143.92, 141.46, 141.43, 129.60, 128.15, 127.84, 127.21, 127.07, 125.23, 120.10, 67.25, 67.19, 52.77, 51.40, 50.41, 47.31, 33.68, 19.07. HRMS $[M+Na]^+$ m/z calc'd for $[C_{41}H_{38}N_2O_5S+Na]^+$: 693.2393, found 693.2383.

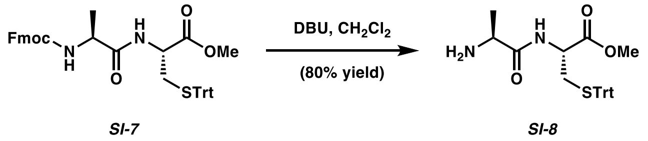

H-*L*-Ala-*L*-Cys(Trt)-OMe (*SI-8*, NMV-X-080): To a solution of ***SI-7*** (0.400 g, 0.596 mmol, 1 equiv) in CH_2Cl_2 (2 mL, 0.3 M), DBU (0.098 mL, 0.656 mmol, 1.1 equiv) was added and reaction mixture was stirred for 15 minutes at room temperature. The reaction mixture was concentrated under vacuum and purified with silica gel using an automated flash column chromatography system (0–15% Methanol/CH_2Cl_2) to afford ***SI-8*** as a colorless oil. (0.214 g, 80% yield). 1H NMR (600 MHz, Methanol-d4) δ 7.63 – 6.86 (m, 15H), 4.23 (dd, J = 8.7, 5.0 Hz, 1H), 3.67-3.65 (m, 1H), 3.64 (s, 3H), 2.78 – 2.42 (m, 2H), 1.36 (d, J = 6.9 Hz, 3H). 13C NMR (126 MHz, Methanol-d4) δ 170.58, 170.58, 144.38, 129.29, 127.64, 126.62, 66.81, 51.89, 51.55, 49.28, 32.83, 17.17. HRMS $[M+Na]^+$ m/z calc'd for $[C_{26}H_{28}N_2O_3S+Na]^+$: 471.1713, found 471.1709.

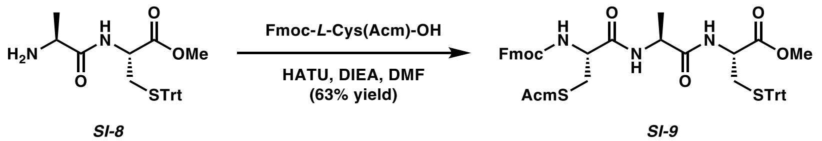

Fmoc-*L*-Cys(Acm)-*L*-Ala-*L*-Cys(Trt)-OMe (*SI-9*, NMV-X-081): To a solution of Fmoc-*L*-Cys(Acm)-OH (0.097 g, 0.235 mmol, 1.1 equiv) in DMF (0.93 mL, 0.25 M), HATU (0.089 g, 0.235 mmol, 1.1 equiv) and DIEA (0.082 mL,0.471 mmol, 2.2 equiv) were added. ***SI-8*** (0.096 g, 0.214 mmol, 1 equiv) was then added as a solution in DMF (0.85 mL, 0.25 M). Reaction mixture was stirred for 2 h. Water was added, and the reaction mixture was extracted with ethyl acetate (2 x 50 mL). Combined organic layers were washed with brine solution (4 X 50 mL). The combined organic layers were dried over anhydrous $MgSO_4$, filtered, and concentrated under vacuum. The residue was purified with silica gel using an automated flash column chromatography system (0–12% MeOH/CH_2Cl_2) to afford ***SI-9*** as a white solid. (0.114 g, 63% yield). ^{1}H NMR (400 MHz, Methanol-*d*$_4$) δ 7.89 (d, *J* = 7.5 Hz, 2H), 7.77 (t, *J* = 6.5 Hz, 2H), 7.55 – 7.28 (m, 18H), 4.60 – 4.42 (m, 5H), 4.40 – 4.29 (m, 3H), 3.76 (s, 3H), 3.19 – 3.09 (m, 1H), 2.97 – 2.86 (m, 1H), 2.74 (qd, *J* = 12.7, 7.4 Hz, 2H), 2.09 (s, 3H), 1.50 (d, *J* = 7.1 Hz, 3H). ^{13}C NMR (101 MHz, Methanol-*d*$_4$) δ 173.95, 173.15, 172.34, 171.77, 171.75, 145.37, 144.71, 142.20, 130.35, 128.74, 128.50, 127.89, 127.66, 125.94, 120.67, 67.99, 67.89, 55.51, 52.93, 52.86, 49.86, 48.01, 41.39, 34.01, 33.81, 22.85, 18.04. LRMS $[M+NH_4]^+$ m/z calc'd for $[C_{47}H_{48}N_4O_7S_2+NH_4]^+$: 862.33, found 862.50.

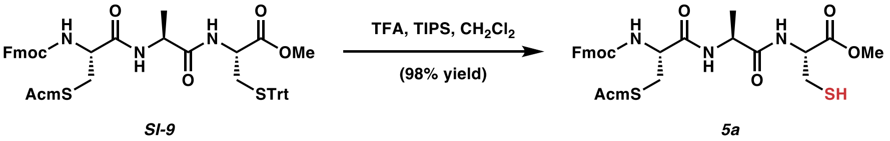

Fmoc-*L*-Cys(Acm)-*L*-Ala-*L*-Cys-OMe (*5a*, NMV-X-082): To a solution of ***SI-9*** (0.092 g, 0.108 mmol, 1 equiv) in CH_2Cl_2 (1 mL, 0.1 M), TIPS (0.110 mL, 0.539 mmol, 5 equiv) was added followed by TFA (0.082 mL, 1.077 mmol, 10 equiv). Reaction mixture was stirred for 1 h. Benzene (2 X 1 mL) was added, and reaction mixture was evaporated to 1/4th of its volume. The residue was purified with silica gel using an automated flash column chromatography system (0–15% MeOH/CH_2Cl_2) to afford ***5a*** as a white solid. (0.0645 g, 98% yield). ^{1}H NMR (400 MHz, Methanol-*d*$_4$) δ 8.38 (d, *J* = 7.0 Hz, 1H), 8.30 (d, *J* = 7.9 Hz, 1H), 8.04 (d, *J* = 7.5 Hz, 2H), 7.92 (dd, *J* = 7.6, 3.7 Hz, 2H), 7.67 (t, *J* = 7.5 Hz, 2H), 7.58 (td, *J* = 7.4, 1.2 Hz, 2H), 4.98 (dt, *J* = 7.8, 5.1 Hz, 1H), 4.76 – 4.60 (m, 5H), 4.56 – 4.47 (m, 2H), 4.04 (s, 3H), 3.29 (dd, *J* = 14.4, 5.2 Hz, 1H), 3.20 (q, *J* = 8.1, 6.7 Hz, 2H), 3.07 (dd, *J* = 14.3, 8.5 Hz, 1H), 2.28 (s, 3H), 1.70 (d, *J* = 7.1 Hz, 3H). ^{13}C NMR (101 MHz, Methanol-*d*$_4$) δ 173.74, 172.87, 172.17, 172.09, 171.19, 144.42, 144.35, 141.90, 128.28, 127.64, 125.66, 120.45, 67.76, 55.21, 52.94, 49.91, 49.81, 47.69, 41.19, 33.65, 26.42, 22.85, 17.67. LRMS [M+H]$^+$ m/z calc'd for [$C_{28}H_{34}N_4O_7S_2$+H]$^+$: 603.19, found 603.20.

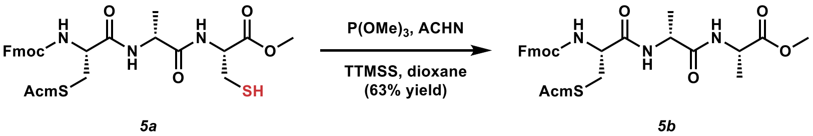

Fmoc-*L*-Cys(Acm)-*L*-Ala-*L*-Ala-OMe (*5b*, NMV-X-084): To a 20-mL microwave vial (with 14/20 septum under an atmosphere of argon), ***5a*** (0.037 g, 0.061 mmol, 1 equiv), dioxane (1.22 mL, 0.05 M), and TTMSS (0.038 mL, 0.122 mmol, 2 equiv) were added. The reaction mixture was purged with argon for few minutes then P(OMe)$_3$ (0.0014 mL, 0.012 mmol, 0.2 equiv) and ACHN (0.0015 g, 0.006 mmol, 0.1 equiv) were added. The reaction vial was sealed and placed in pre-heated silicon oil bath at 88 °C and stirred for 58 h. The reaction mixture was cooled to room temperature and concentrated under vacuum. The crude was purified using silica gel with an automated flash column chromatography system using 0-15% MeOH/CH_2Cl_2 as an eluent to afford ***5b*** as white solid (0.022 g, 63% yield), Recovered starting material 12 mg (32%). ^{1}H NMR (400 MHz, Methanol-*d*$_4$) δ 7.94 (dt, *J* = 7.6, 1.0 Hz, 2H), 7.81 (dd, *J* = 7.7, 3.0 Hz, 2H), 7.58 – 7.49 (m, 2H), 7.45 (td, *J* = 7.5, 1.2 Hz, 2H), 4.62 – 4.45 (m, 6H), 4.43 – 4.34 (m, 2H), 3.85 (s, 3H), 3.20 – 3.14 (m, 1H), 2.93 (dd, *J* = 14.2, 9.1 Hz, 1H), 2.12 (s, 3H), 1.53 (dd, *J* = 7.2, 1.9 Hz, 6H). ^{13}C NMR (101 MHz, Methanol-*d*$_4$) δ 173.10, 173.08, 172.21, 172.17, 171.49, 143.79, 141.18, 127.37, 126.75, 124.84, 119.50, 66.74, 54.54, 51.30, 48.77, 40.19, 33.47, 32.51, 21.31, 16.66, 15.95. HRMS [M+H]$^+$ m/z calc'd for [$C_{28}H_{34}N_4O_7S$+H]$^+$: 571.2220, found 571.2214.

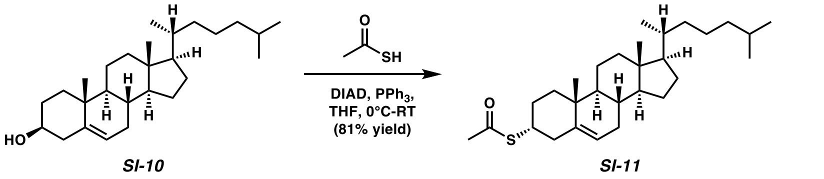

Cholest-5-ene-3α-thiyl acetate (*SI-11*, RMI-IV-033). Cholesterol ***SI-10*** (0.35 g, 0.905 mmol, 1 equiv) was dissolved in THF (3 mL, 0.3 M) and reaction mixture was cooled in an ice/water bath to 0°C. In a separate vial, PPh_3 (0.285 g, 1.086 mmol, 1.2 equiv) and DIAD solution (0.21 mL, 1.086 mmol, 1.2 equiv) were dissolved in THF (6 mL, 0.1 M total with respect to start) then added to the reaction mixture. Thioacetic acid (0.071 mL, 0.995 mmol, 1.1 equiv) was then added. Reaction was stirred for 3h. Reaction mixture was evaporated and purified using silica gel using an automated flash column chromatography system to afford ***SI-11*** as white solid (0.325 g, 81% yield). ^{1}H NMR (499 MHz, Chloroform-*d*) δ 5.31 (dt, *J* = 5.6, 2.1 Hz, 1H), 3.99 (dq, *J* = 4.5, 2.3 Hz, 1H), 2.81 – 2.71 (m, 1H), 2.28 (s, 3H), 2.09 – 1.90 (m, 4H), 1.83 (dtd, *J* = 13.4, 9.4, 5.8 Hz, 1H), 1.70 (ddt, *J* = 16.3, 5.9, 2.5 Hz, 2H), 1.65 – 1.55 (m, 2H), 1.55 – 1.37 (m, 5H), 1.37 – 1.20 (m, 5H), 1.20 – 1.11 (m, 3H), 1.07 (dd, *J* = 8.2, 6.4 Hz, 2H), 1.06 – 0.97 (m, 6H), 0.97 – 0.83 (m, 9H), 0.67 (s, 3H). ^{13}C NMR (126 MHz, Chloroform-*d*) δ 195.82, 139.08, 122.53, 77.26, 77.01, 76.76, 56.70, 56.15, 50.12, 43.20, 42.27, 39.73, 39.50, 37.66, 37.18, 36.18, 35.80, 35.53, 31.75, 31.71, 30.96, 28.23, 28.00, 27.53, 24.25, 23.85, 22.82, 22.56, 20.72, 19.14, 18.71, 11.83.

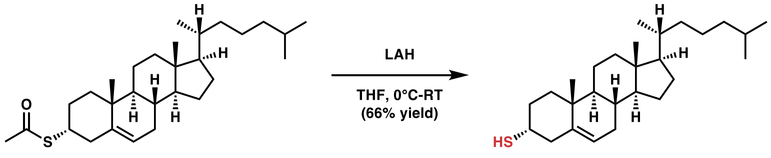

SI-11 → *6a*

Cholest-5-ene-3α-thiol (*6a*, RMI-IV-036). To a 0°C THF (2.5 mL, 0.6 M with respect to LAH) solution of LAH (0.055 g, 1.462 mmol, 2 equiv) under an atmosphere of argon, ***SI-11*** (0.0325g, 0.73 mmol, 1 equiv) solution in THF (6.5 mL, 0.1 M with respect to start) was added portion wise via syringe. Reaction mixture was stirred overnight. Reaction mixture was concentrated under vacuum and purified using silica gel on an automated flash column chromatography system (0-20%EtOAc/Hexanes) to afford ***6a*** as white solid (0.1947g, 66% yield). ^{1}H NMR (499 MHz, Chloroform-*d*) δ 5.37 (dt, *J* = 5.0, 2.1 Hz, 1H), 3.43 – 3.36 (m, 1H), 2.81 (ddt, *J* = 14.1, 5.1, 2.6 Hz, 1H), 2.06 – 1.90 (m, 4H), 1.84 (dtd, *J* = 13.3, 9.4, 5.7 Hz, 1H), 1.70 – 1.50 (m, 7H), 1.50 – 1.37 (m, 3H), 1.37 – 1.35 (m, 1H), 1.35 – 1.26 (m, 2H), 1.26 – 0.97 (m, 12H), 0.94 – 0.84 (m, 8H), 0.68 (s, 3H). ^{13}C NMR (126 MHz, Chloroform-*d*) δ 137.92, 123.96, 77.24, 76.99, 76.74, 56.72, 56.13, 50.22, 42.29, 40.67, 39.74, 39.51, 37.88, 37.24, 36.18, 35.80, 33.04, 31.88, 31.74, 30.16, 28.23, 28.00, 24.26, 23.84, 22.81, 22.55, 20.74, 19.11, 18.70, 11.84.

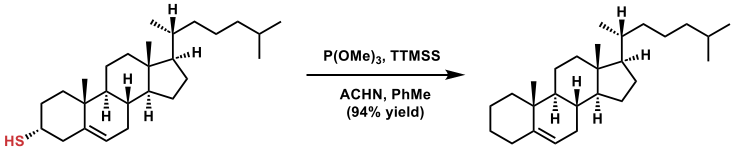

6a → *6b*

Cholest-5-ene (*6b*, RMI-IV-041). To a 20-mL microwave vial (with 14/20 septum under an atmosphere of argon), ***6a*** (0.19 g, 0.47 mmol, 1 equiv), PhMe (9.5 mL, 0.05 M) and TTMSS (0.29 mL, 0.94 mmol, 2 equiv) were added. The reaction mixture was purged with argon for few minutes then $P(OMe)_3$ (0.011 mL, 0.094 mmol, 0.2 equiv) and ACHN (0.0115 g, 0.047 mmol, 0.1 equiv) were added. The reaction vial was sealed and placed in pre-heated oil bath at 88 °C and stirred for 14 h. The reaction mixture was cooled to room temperature and concentrated under vacuum. The crude was purified using silica gel with an automated flash column chromatography system using 0-10% EtOAc in hexanes as an eluent to afford ***6b*** as white solid (0.165 g, 94% yield). ^{1}H NMR (499 MHz, Chloroform-*d*) δ 5.27 (dt, *J* = 4.6, 2.0 Hz, 1H), 2.28 – 2.19 (m, 1H), 2.04 – 1.90 (m, 3H), 1.86 – 1.77 (m, 2H), 1.77 – 1.69 (m, 1H), 1.63 – 1.46 (m, 7H), 1.44 (dt, *J* = 8.5, 3.2 Hz, 1H), 1.42 – 1.30 (m, 4H), 1.29 – 1.24 (m, 2H), 1.24 – 1.17 (m, 2H), 1.17 – 1.06 (m, 5H), 1.06 – 0.94 (m, 7H), 0.94 – 0.83 (m, 9H), 0.68 (s, 3H). ^{13}C NMR (126 MHz, Chloroform-*d*) δ 143.61, 118.98, 77.23, 76.98, 76.72, 62.98, 56.88, 56.19, 50.60, 42.30, 39.89, 39.88, 39.54, 37.52, 36.22, 35.83, 32.91, 31.89, 31.84, 28.27, 28.07, 28.02, 26.00, 24.29, 23.87, 22.83, 22.58, 22.57, 21.97, 20.78, 19.46, 18.73, 11.87. *These data are consistent with previously reported data.*[3]

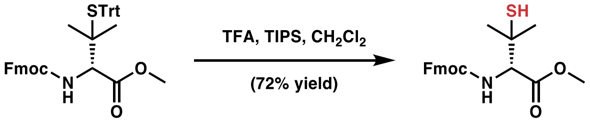

SI-12 → *7a*

Fmoc-*D*-Pen-OMe (*7a*, RMI-V-019). To a solution of ***SI-12*** (0.16 g, 0.255 mmol, 1 equiv) in CH_2Cl_2 (2.5 mL, 0.1 M), was added TIPS (0.26 mL, 1.274 mmol, 5 equiv) followed by TFA (0.2 mL, 2.549 mmol, 10 equiv). Reaction mixture was stirred for 45 min. Reaction mixture was concentrated and the residue was purified using silica gel with an automated flash column chromatography system using 0-50% EtOAc in hexanes as an eluent to afford ***7a*** as white solid (0.071 g, 72% yield). ^{1}H NMR (400 MHz, Chloroform-*d*) δ 7.81 – 7.73 (m, 2H), 7.64 – 7.53 (m, 2H), 7.41 (tt, *J* = 7.5, 1.5 Hz, 2H), 7.32 (tt, *J* = 7.5, 1.5 Hz, 2H), 5.81 – 5.73 (m, 1H), 4.46 (dd, *J* = 10.6, 7.5 Hz, 1H), 4.40 (t, *J* = 8.3 Hz, 2H), 4.24 (t, *J* = 6.9 Hz, 1H), 3.77 (s, 3H), 1.98 (s, 1H), 1.50 (s, 3H), 1.39 (s, 3H). ^{13}C NMR (101 MHz, Chloroform-*d*) δ 170.96, 156.14, 143.62, 141.32, 127.75, 127.08, 120.01, 119.99, 77.31, 76.99, 76.67, 67.32, 62.63, 52.21, 47.15, 46.47, 30.80, 29.37. HRMS $[M+H]^+$ calc'd for $[C_{21}H_{23}NO_4S+H]$: *m/z* 386.1421, found 386.1419.

[3] Yasuda, H.; Uenoyama, Y.; Nobuta, O.; Kobayashi, S.; Ryu, I. Radical chain reactions using THP as a solvent. *Tetrahedron Lett.* ***2008***, *49*, 367-370. **https://doi.org/10.1016/j.tetlet.2007.11.039**

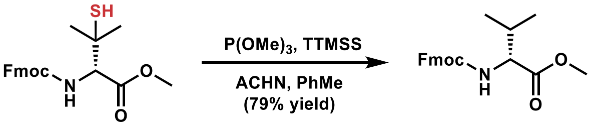

7a *7b*

Fmoc-*D*-Val-OMe (*7b*, RMI-V-032). To a 15 mL microwave vial (with 14/20 septum under an atmosphere of argon), ***7a*** (0.081 g, 0.211 mmol, 1 equiv), PhMe (4.2 mL, 0.05 M), and TTMSS (0.13 mL, 0.422 mmol, 2 equiv) were added. The reaction mixture was purged with argon for few minutes then $P(OMe)_3$ (0.005 mL, 0.042 mmol, 0.2 equiv) and ACHN (0.0052g, 0.021 mmol, 0.1 equiv) were added. The reaction vial was sealed and placed in pre-heated silicon oil bath at 88 °C and stirred for 45 h. The reaction mixture was cooled to room temperature and concentrated under vacuum. The crude was purified using silica gel with an automated flash column chromatography system using 0-50% EtOAc in hexanes as an eluent to afford ***7b*** as white solid (0.0585 g, 79% yield). ^{1}H NMR (400 MHz, Chloroform-*d*) δ 7.77 (d, *J* = 7.5 Hz, 2H), 7.61 (dd, *J* = 7.6, 3.2 Hz, 2H), 7.40 (t, *J* = 7.4 Hz, 2H), 7.32 (td, *J* = 7.4, 1.3 Hz, 2H), 5.33 (d, *J* = 9.2 Hz, 1H), 4.47 – 4.37 (m, 2H), 4.33 (dd, *J* = 9.2, 4.9 Hz, 1H), 4.24 (t, *J* = 7.1 Hz, 1H), 3.76 (s, 3H), 2.25 – 2.11 (m, 1H), 0.95 (dd, *J* = 22.3, 6.8 Hz, 6H). ^{13}C NMR (101 MHz, Chloroform-*d*) δ 172.56, 156.20, 143.92, 143.78, 141.31, 130.05, 127.68, 127.05, 125.07, 119.97, 119.96, 77.35, 77.03, 76.71, 67.02, 59.04, 52.15, 47.22, 31.31, 18.94, 17.64. HRMS $[M+H]^+$ calc'd for $[C_{21}H_{23}NO_4+H]$: *m/z 354*.1700, found 354.1698.

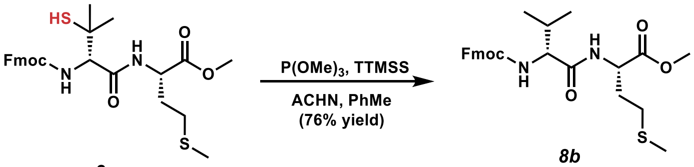

8a *8b*

Fmoc-*D*-Val-*L*-Met-OMe (*8b*, RMI-XII-022). To a 20-mL microwave vial (with 14/20 septum under an atmosphere of argon), ***8a*** (0.1405 g, 0.272 mmol, 1 equiv), PhMe (5.6 mL, 0.05 M), and TTMSS (0.168 mL, 0.5444 mmol, 2 equiv) were added. The reaction mixture was purged with argon for few minutes then $P(OMe)_3$ (0.0064 mL, 0.054 mmol, 0.2 equiv) and ACHN (0.0066 g, 0.027 mmol, 0.1 equiv) were added. The reaction vial was sealed and placed in pre-heated silicon oil bath at 88 °C and stirred for 20 h. The reaction mixture was cooled to room temperature and concentrated under vacuum. The crude was purified using silica gel with an automated flash column chromatography system using 0-90% EtOAc in hexanes as an eluent to afford ***8b*** as white solid (0.1 g, 76% yield). ^{1}H NMR (400 MHz, Chloroform-*d*) δ 7.76 (d, *J* = 7.5 Hz, 2H), 7.59 (d, *J* = 7.4 Hz, 2H), 7.40 (dd, *J* = 8.4, 6.9 Hz, 2H), 7.31 (td, *J* = 7.5, 1.2 Hz, 2H), 6.71 (d, *J* = 7.7 Hz, 1H), 5.38 (d, *J* = 8.5 Hz, 1H), 4.72 (td, *J* = 7.4, 5.1 Hz, 1H), 4.41 (tt, *J* = 17.5, 8.7 Hz, 2H), 4.22 (t, *J* = 7.0 Hz, 1H), 4.14 – 4.04 (m, 1H), 3.73 (s, 3H), 2.49 (t, *J* = 7.4 Hz, 2H), 2.16 (td, *J* = 13.1, 6.9 Hz, 2H), 2.06 (s, 3H), 1.99 (dt, *J* = 14.3, 7.2 Hz, 1H), 0.96 (dd, *J* = 16.1, 6.6 Hz, 6H). ^{13}C NMR (101 MHz, Chloroform-*d*) δ 172.00, 171.04, 143.81, 143.72, 141.29, 127.71, 127.07, 125.01, 119.98, 119.96, 77.32, 77.01, 76.69, 67.13, 52.55, 51.55, 47.16, 31.39, 30.97, 29.96, 19.25, 17.61, 15.42. HRMS $[M+H]^+$ calc'd for $[C_{26}H_{32}N_2O_5S+H]$: *m/z* 485.2105, found 485.2096.

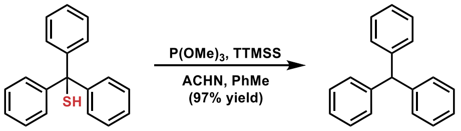

9a *9b*

Triphenyl methane (*9b*, RMI-IV-091): To a 20-mL microwave vial (with 14/20 septum under an atmosphere of argon), ***9a*** (0.15 g, 0.549 mmol, 1 equiv), PhMe (11 mL, 0.05 M), and TTMSS (0.34 mL, 1.09 mmol, 2 equiv) were added. The reaction mixture was purged with argon for few minutes then $P(OMe)_3$ (0.0128 mL, 0.109 mmol, 0.2 equiv) and ACHN (0.013 g, 0.055 mmol, 0.1 equiv) were added. The reaction vial was sealed and placed in pre-heated silicon oil bath at 88 °C and stirred for 21 h. The reaction mixture was cooled to room temperature and concentrated under vacuum. The crude was purified using silica gel with an automated flash column chromatography system using 0-20% EtOAc in hexanes as an eluent to afford ***9b*** as white solid (0.129 g, 97% yield). ^{1}H NMR (499 MHz, Chloroform-*d*) δ 7.29 (dd, *J* = 8.3, 6.8 Hz, 6H), 7.26 – 7.18 (m, 3H), 7.13 (dd, *J* = 7.5, 1.6 Hz, 6H), 5.56 (s, 1H). ^{13}C NMR (126 MHz, Chloroform-*d*) δ 143.88, 129.44, 128.28, 126.28, 77.26, 77.00, 76.75, 56.83. *These data are consistent with previously reported data.*[4]

[4] Prakash, G. S.; Panja, C.; Shakhmin, A.; Shah, E.; Mathew, T., Olah, G. A. BF_3– H_2O catalyzed hydroxyalkylation of aromatics with aromatic aldehydes and dicarboxaldehydes: efficient synthesis of triarylmethanes, diarylmethylbenzaldehydes, and anthracene derivatives. *J. Org. Chem.* **2009**, *74*, 8659-8668. https://doi.org/10.1021/jo901668j

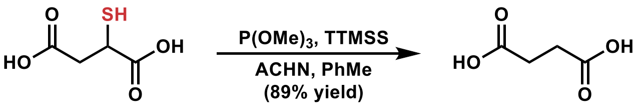

10a **10b**

Succinic acid (*10b*, RMI-VII-041). To a 20-mL microwave vial (with 14/20 septum under an atmosphere of argon), mercaptosuccinic acid ***10a*** (0.12 g, 0.799 mmol, 1 equiv), PhMe (16 mL, 0.05 M), and TTMSS (0.49 mL, 1.598 mmol, 2 equiv) were added. The reaction mixture was purged with argon for few minutes then $P(OMe)_3$ (0.0188 mL, 0.16 mmol, 0.2 equiv) and ACHN (0.012 g, 0.078 mmol, 0.1 equiv) were added. The reaction vial was sealed and placed in pre-heated oil bath at 88 °C and stirred for 27 h. The reaction mixture was cooled to room temperature and concentrated under vacuum. The crude was dissolved in hexane and cooled it for 5 hours in -4 °C refrigerator. The precipitated solid was filtered and washed with cold hexane (2 mL X 5) to afford ***10b*** as white solid (0.084 g, 89% yield). ^{1}H NMR (400 MHz, Actonitrile-*d*$_3$) δ 2.56 (s, 4H). ^{13}C NMR (101 MHz, Acetonmitrile-*d*$_3$) δ 174.88, 29.45. *These data are consistent with previously reported data.*[5]

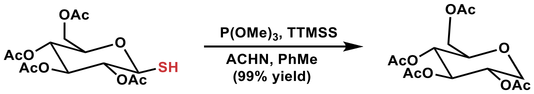

11a **11b**

(2R,3R,4R,5S)-2-(acetoxymethyl)tetrahydro-2H-pyran-3,4,5-triyl triacetate (*11b*, RMI-VII-040). To a 20-mL microwave vial (with 14/20 septum under an atmosphere of argon), ***11a*** (0.2 g, 0.549 mmol, 1 equiv), PhMe (11 mL, 0.05 M), and TTMSS (0.34 mL, 1.098 mmol, 2 equiv) were added. The reaction mixture was purged with argon for few minutes then $P(OMe)_3$ (0.013 mL, 0.11 mmol, 0.2 equiv) and ACHN (0.013 g, 0.055 mmol, 0.1 equiv) were added. The reaction vial was sealed and placed in pre-heated silicon oil bath at 88 °C and stirred for 27 h. The reaction mixture was cooled to room temperature and concentrated under vacuum. The crude was purified using silica gel with an automated flash column chromatography system using 0-90% EtOAc in hexanes as an eluent to afford ***11b*** as white solid (0.1809 g, 99% yield). ^{1}H NMR (400 MHz, Chloroform-*d*) δ 5.20 (t, *J* = 9.4 Hz, 1H), 5.07 – 4.95 (m, 2H), 4.24 – 4.10 (m, 3H), 3.59 (ddd, *J* = 10.1, 4.9, 2.3 Hz, 1H), 3.30 (t, *J* = 10.9 Hz, 1H), 2.09 (d, *J* = 1.3 Hz, 3H), 2.03 (d, *J* = 2.2 Hz, 9H). ^{13}C NMR (101 MHz, Chloroform-*d*) δ 170.66, 169.76, 169.51, 77.32, 77.00, 76.68, 76.45, 73.69, 68.94, 68.40, 66.86, 62.19, 20.72, 20.69, 20.67, 20.60. *These data are consistent with previously reported data.*[6]

Table *SI-01*: Peptide desulfurization screening

---

[5] Singh, S.; Verma, M.; Singh, K. N. Superoxide Ion Induced Oxidation of γ - Lactones to γ - Ketocarboxylic Acids. Synth. Commun. 2004, 34, 4471-4475. https://doi.org/10.1081/SCC-200043177

[6] Ruttens, B.; Blom, P.; Van Hoof, S.; Hubrecht, I.; Van der Eycken, J.; Sas, B.; Van hemel J.; Vandenkerckhove, J. Carbohydrate-based macrolides prepared via a convergent ring closing metathesis approach: in search for novel antibiotics. *J. Org. Chem.* **2007**, *72*, 5514-5522. https://doi.org/10.1021/jo061929q

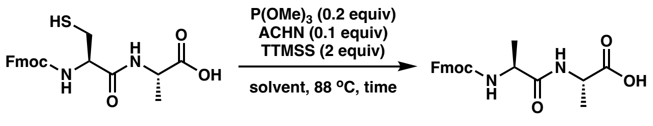

entry[a]	solvent	time (h)	product conversion (%)[b]
1	acetonitrile	24	14
2	acetonitrile/water (2:3)	24	28
3	acetonitrile/water (3:2)	24	49
4[c]	acetonitrile/water (3:2)	12 +12	62
5	1,4-dioxane/water (3:2)	24	43

[a] starting *SI-13* (0.100 g, 0.241 mmol, 1 equiv), $P(OMe)_3$ (5.7 μL, 0.048 mmol, 0.2 equiv), ACHN (5.9 mg, 0.024 mmol, 0.1 equiv), TTMSS (0.149 mL, 0.482 mmol, 2 equiv), solvent (0.05 M), 88 °C, [b] conversion of the product was measured using HPLC at 280 nm, [c] after 12 h, second batch of $P(OMe)_3$ (5.7 μL, 0.048 mmol, 0.2 equiv), and ACHN (5.9 mg, 0.024 mmol, 0.1 equiv) were added

Table *SI-01*, Entry 1 (NMV-XII-039): Conditions: Acetonitrile, 24 h

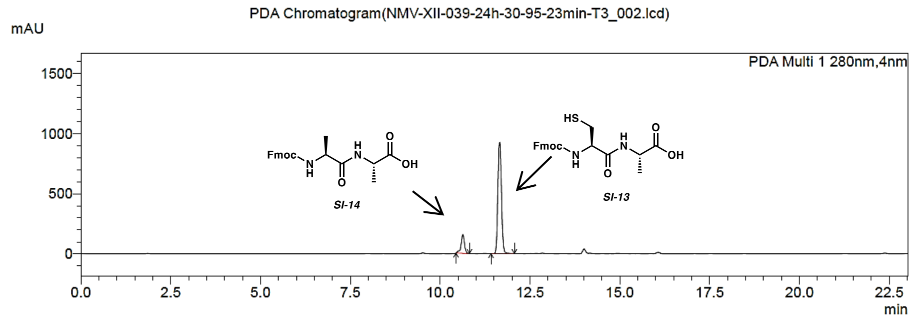

PDA Peak Table(NMV-XII-039-24h-30-95-23min-T3_002.lcd)
PDA Ch1 280nm

Peak#	Ret. Time	Area	Height	Area%
1	10.629	1027351	154230	13.833
2	11.654	6399459	925751	86.167
Total		7426810	1079981	100.000

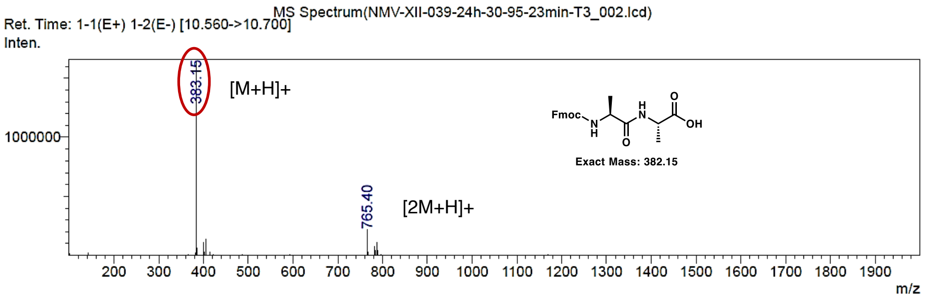

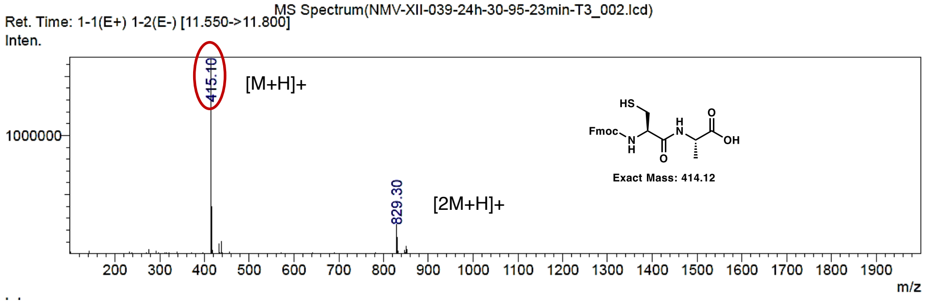

Figure *SI-22*: Method gradient: 30% acetonitrile/water (3 minutes), 30-95% acetonitrile/water (over 15 minutes), and 95% acetonitrile/water (5 minutes), using HPLC T3 analytical column and corresponding mass scans for starting material and desulfurized product peak

Table *SI-01*, Entry 2 (NMV-XII-041): Conditions: Acetonitrile/Water (2:3), 24 h

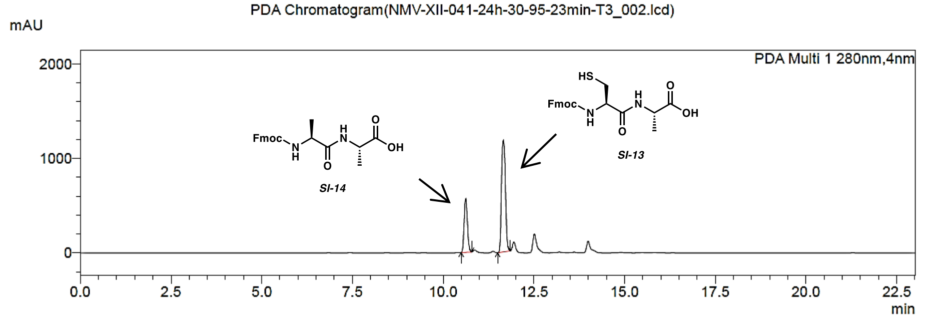

PDA Peak Table(NMV-XII-041-24h-30-95-23min-T3_002.lcd)
PDA Ch1 280nm

Peak#	Ret. Time	Area	Height	Area%
1	10.621	3538077	568000	28.372
2	11.660	8932351	1180935	71.628
Total		12470428	1748936	100.000

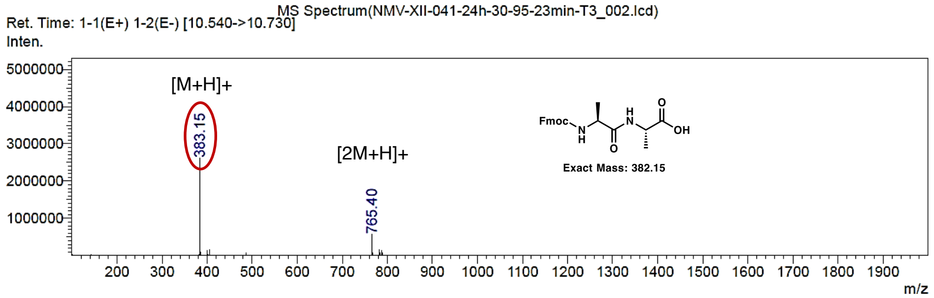

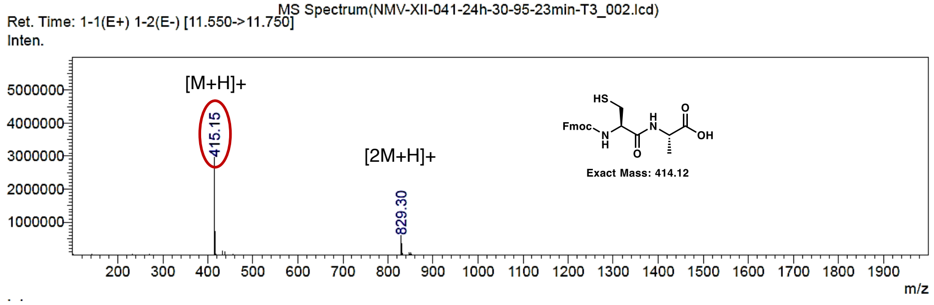

Figure *SI-23*: Method gradient: 30% acetonitrile/water (3 minutes), 30-95% acetonitrile/water (over 15 minutes), and 95% acetonitrile/water (5 minutes), using HPLC T3 analytical column and corresponding mass scans for starting material and desulfurized product peak

Table *SI-01*, Entry 3 (NMV-XII-040): Conditions: Acetonitrile/Water (3:2), 24 h

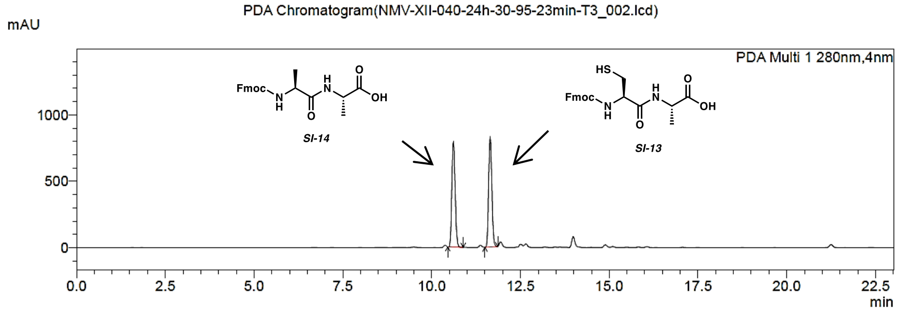

PDA Peak Table(NMV-XII-040-24h-30-95-23min-T3_002.lcd)
PDA Ch1 280nm

Peak#	Ret. Time	Area	Height	Area%
1	10.612	5106505	789052	49.198
2	11.647	5272921	823475	50.802
Total		10379426	1612527	100.000

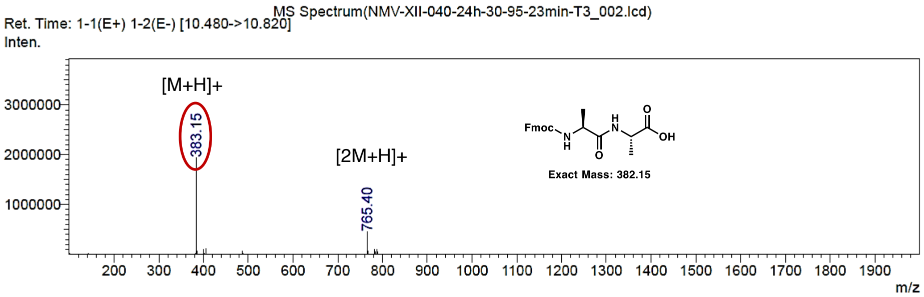

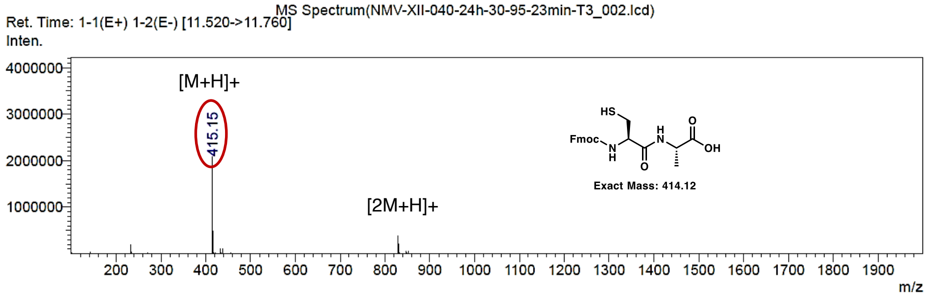

Figure *SI-24*: Method gradient: 30% acetonitrile/water (3 minutes), 30-95% acetonitrile/water (over 15 minutes), and 95% acetonitrile/water (5 minutes), using HPLC T3 analytical column and corresponding mass scans for starting material and desulfurized product peak.

Table *SI-01*, Entry 4 (NMV-XII-051): Conditions: Acetonitrile/Water (3:2), 12h + 12 h

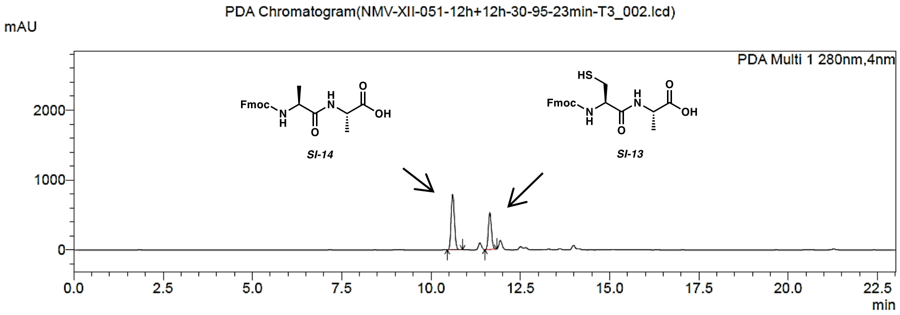

PDA Peak Table(NMV-XII-051-12h+12h-30-95-23min-T3_002.lcd)
PDA Ch1 280nm

Peak#	Ret. Time	Area	Height	Area%
1	10.606	5149370	788051	61.705
2	11.646	3195736	522020	38.295
Total		8345106	1310072	100.000

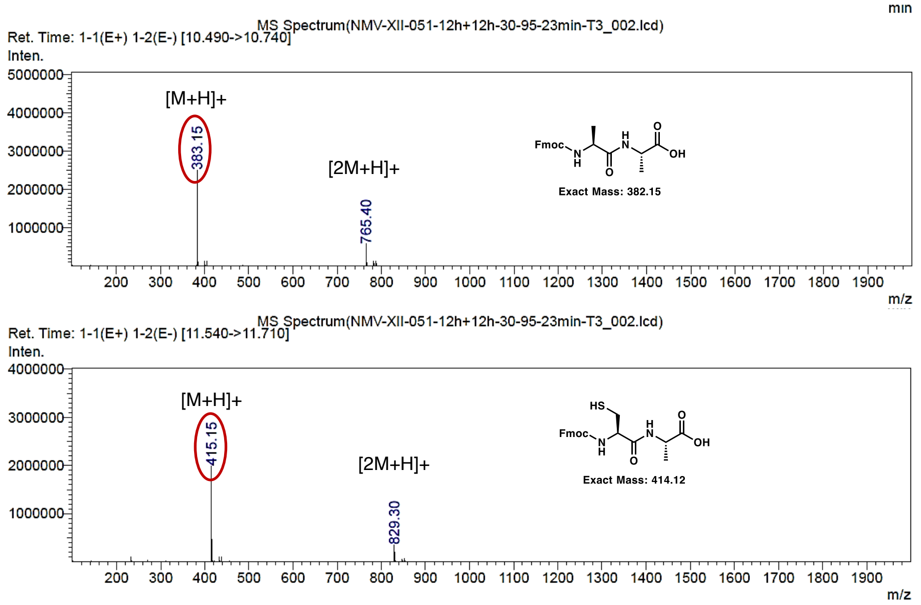

Figure *SI-25*: Method gradient: 30% acetonitrile/water (3 minutes), 30-95% acetonitrile/water (over 15 minutes), and 95% acetonitrile/water (5 minutes), using HPLC T3 analytical column and corresponding mass scans for starting material and desulfurized product peak.

Note: The following by-product traces were observed in entries 2, 3 and 4

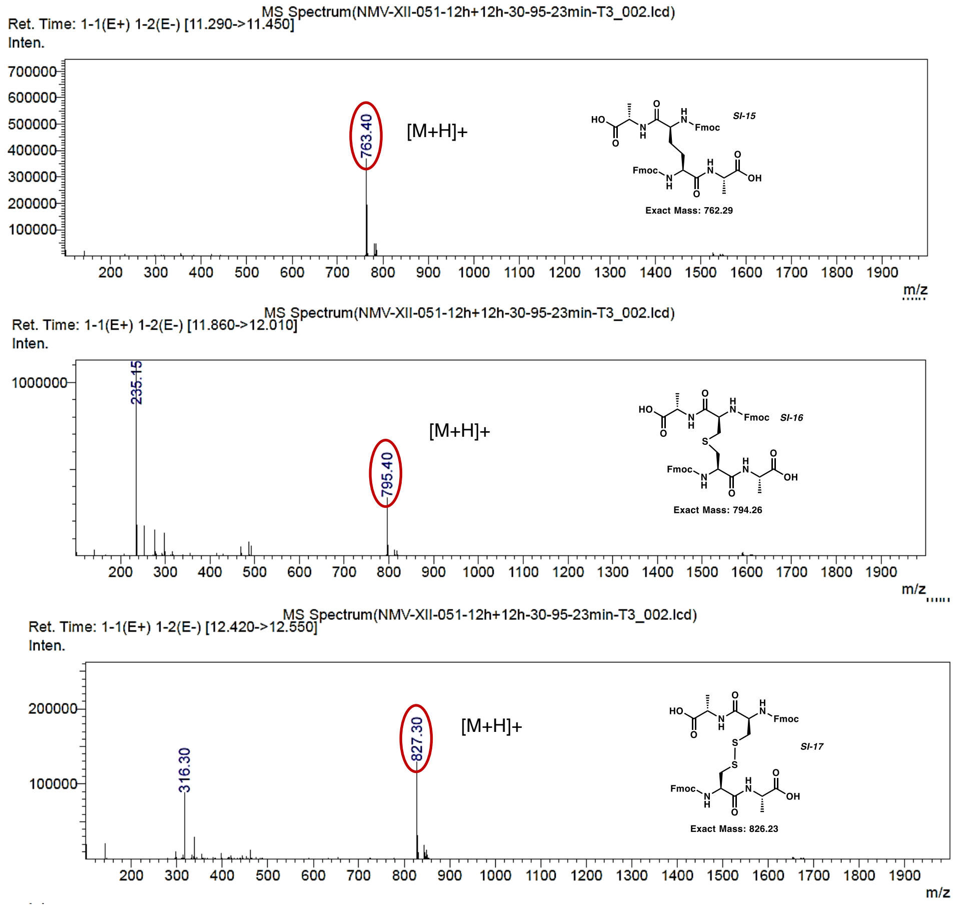

Figure *SI-26*: Mass scans for by-product traces (entry 4 reaction)

Table *SI-01*, Entry 5 (NMV-XII-054): Conditions: 1,4-dioxane/Water (3:2), 24 h

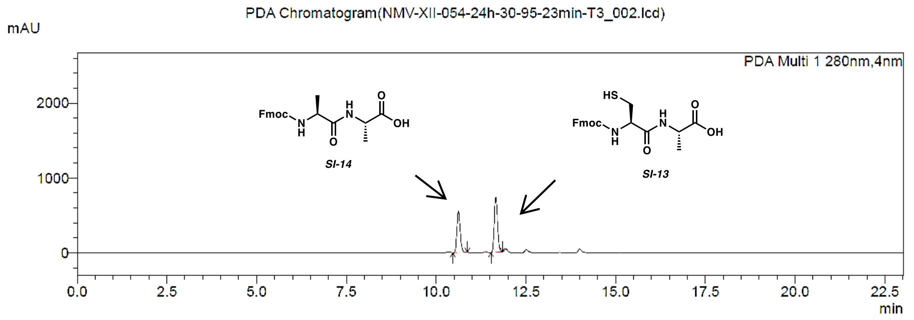

PDA Peak Table(NMV-XII-054-24h-30-95-23min-T3_002.lcd)
PDA Ch1 280nm

Peak#	Ret. Time	Area	Height	Area%
1	10.621	3477433	551803	42.564
2	11.663	4692507	737086	57.436
Total		8169940	1288889	100.000

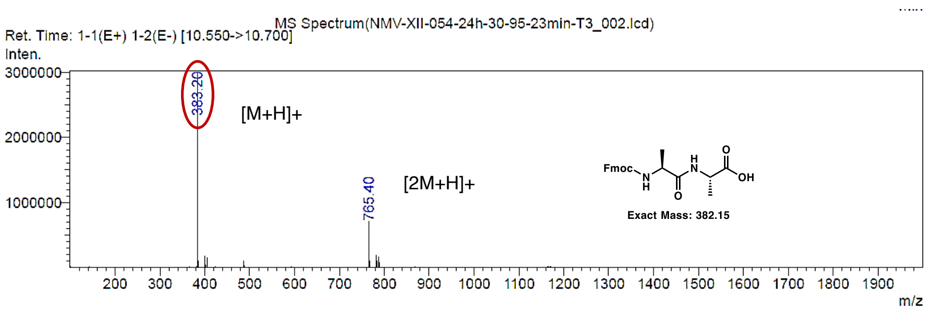

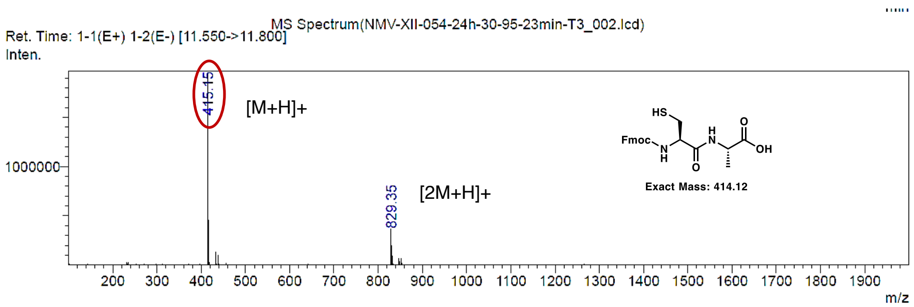

Figure *SI-27*: Method gradient: 30% acetonitrile/water (3 minutes), 30-95% acetonitrile/water (over 15 minutes), and 95% acetonitrile/water (5 minutes), using HPLC T3 analytical column and corresponding mass scans for starting material and desulfurized product peak.

Compatibility of the additives:

Procedure for additive screening: In a 20 mL vial (Environmental Express APC1670 Clear VOA vials), start material (27.8 mg, 0.2 mmol, 1 equiv) was dissolved in PhMe (4 mL, 0.05 M). TTMSS (0.123 mL, 0.4 mmol, 2 equiv) was added. Vial was closed with cap and N_2 was bubbled for 5 min. $P(OMe)_3$ (0.0047 mL, 0.04 mmol, 0.2 equiv) and ACHN (0.0049 mg, 0.02 mmol, 0.1 equiv) were added. To this additive (0.2 mmol, 1 equiv) was added. The reactions vials were placed in the pre-heated reaction block and heated at 88 °C for 3 hours.

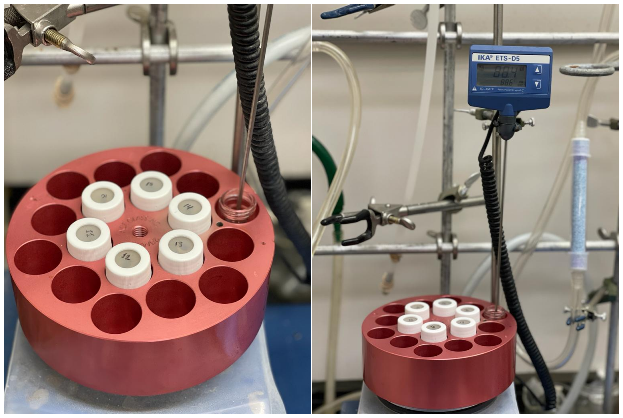

Figure *SI-28*: Batch screening with different additives

Calibration for reaction yield: The GCMS yield of the product was measured using 1,3,5-trimethoxybenzene as an internal standard (Rxi-5Sil MS Column). In case of unreacted starting material, the GCMS conversion was reported.

Method 1: Initial Temperature 50 °C-hold 5 min, increment 20 °C to reach 100 °C-hold 4 min, increment 40 °C to reach 250 °C-hold 4 min, increment 40 °C to reach 320 °C-hold 10 min (total 31 min)

Method 2: Initial Temperature 50 °C-hold 5 min, increment 10 °C to reach 150 °C, increment 30 °C to reach 250 °C, increment 30 ºC to reach 320 °C-hold 10 min (total 31 min)

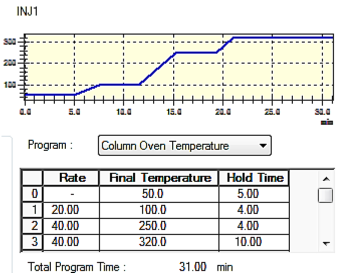

Method 1

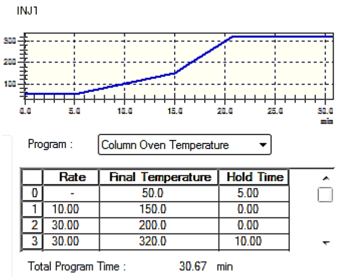

Method 2

Calibration graph for product yield:

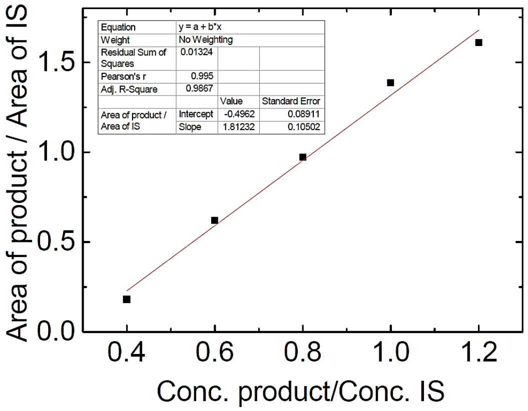

After the reaction was complete, 1,3,5-trimethoxybenzene (0.2 mmol) was added as an internal standard. A 50 μL sample was removed from the reaction vessel and diluted to 200 μL by adding 150 μL EtOAc. This sample was injected in the Shimadzu-GCMS, and the GCMS yield was calculated with respect to internal standard.

The GCMS profile for each reaction is depicted below **Table 4**.

Table 4: Desulfurization outcome in the presence of unreactive/stable additives

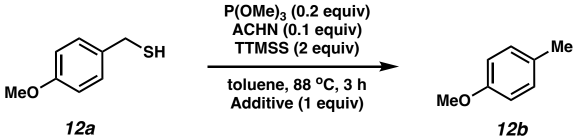

Entry[a]	Addtive	GC Yield *12b* (%)[b]	Unreacted *12a* (%)[b]	Entry[a]	Addtive	GC Yield *12b* (%)[b]	Unreacted *12a* (%)[b]
1	None	87	0	6	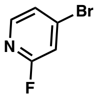	0	100
2	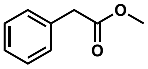	87	0	7	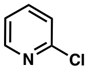	57	0
3	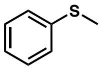	91	7	8	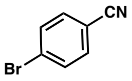	4	95
4	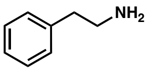	93	0	9	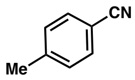	81	0
5	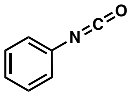	59	0	10	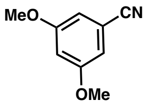	55	0

[a] Reaction conditions: (4-methoxyphenyl)methanethiol (0.2 mmol, 1 equiv), $P(OMe)_3$ (0.04 mmol, 0.2 equiv), ACHN (0.02 mmol, 0.1 equiv), TTMSS (0.4 mmol, 2 equiv), toluene (0.05 M), 88 °C, 3 h [b] Caluclated using GCMS with 1,3,5-trimethoxybenzene as internal standard.

Table 4, Entry 1 (NMV-XII-010): No additive

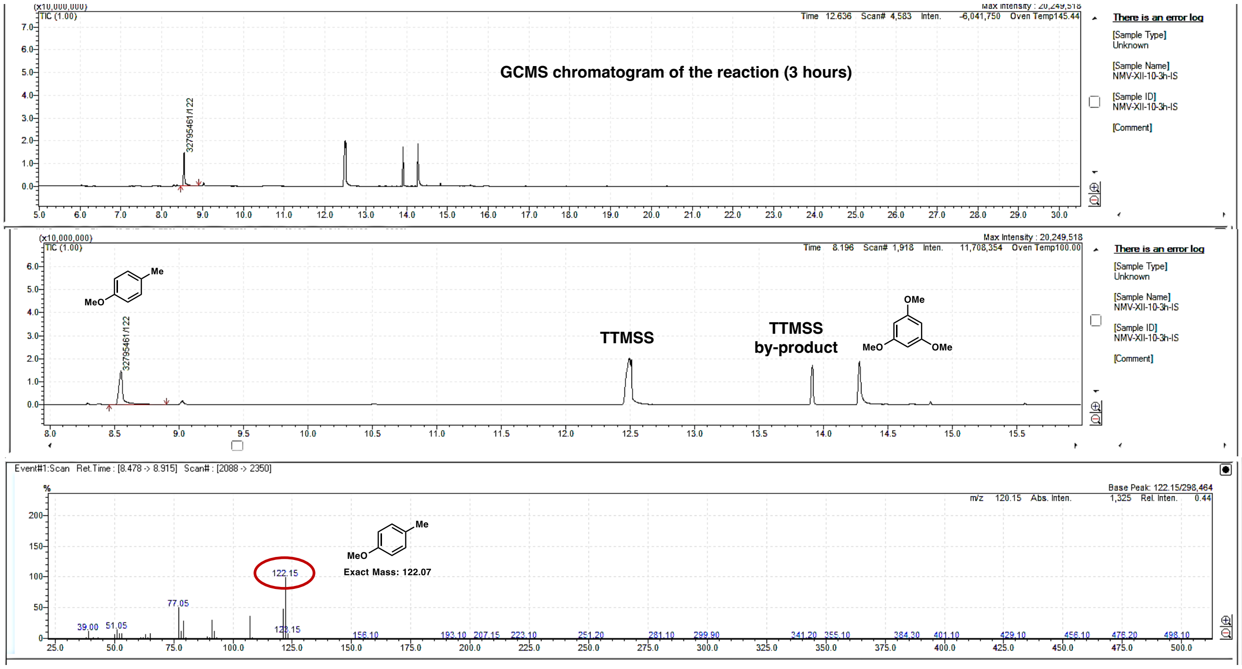

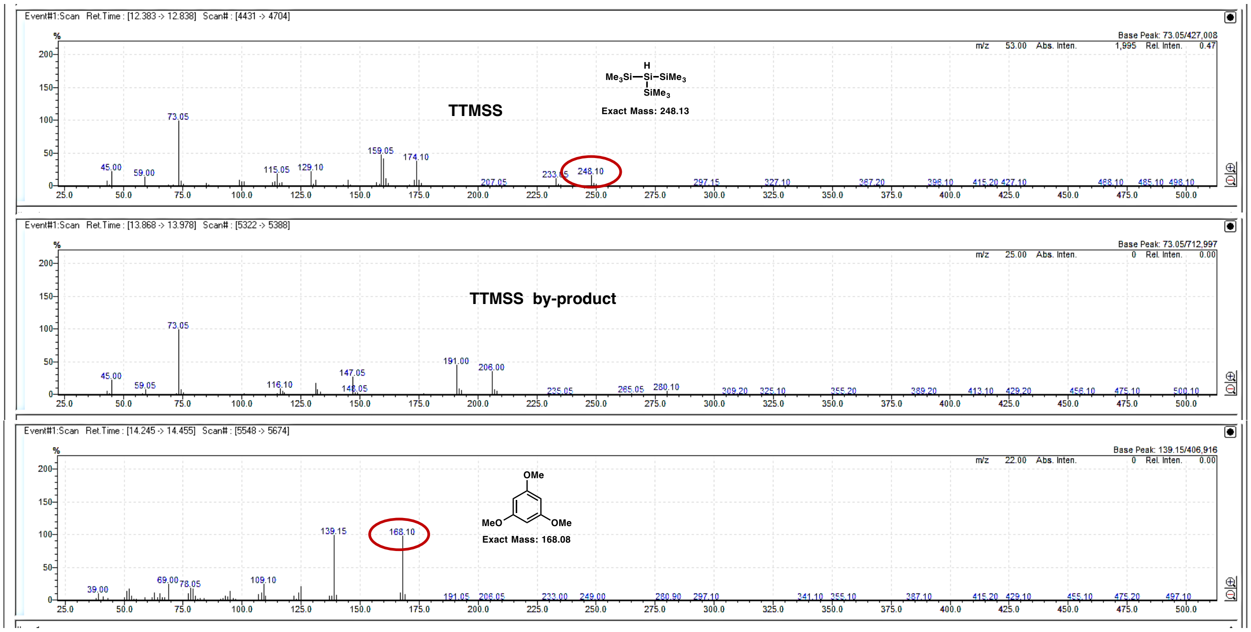

Figure *SI-28*: GCMS chromatogram and corresponding mass scans of the respective peaks

Table 4, Entry 1 (NMV-XII-014): Additive: Methyl 2-phenylacetate (0.2 mmol)

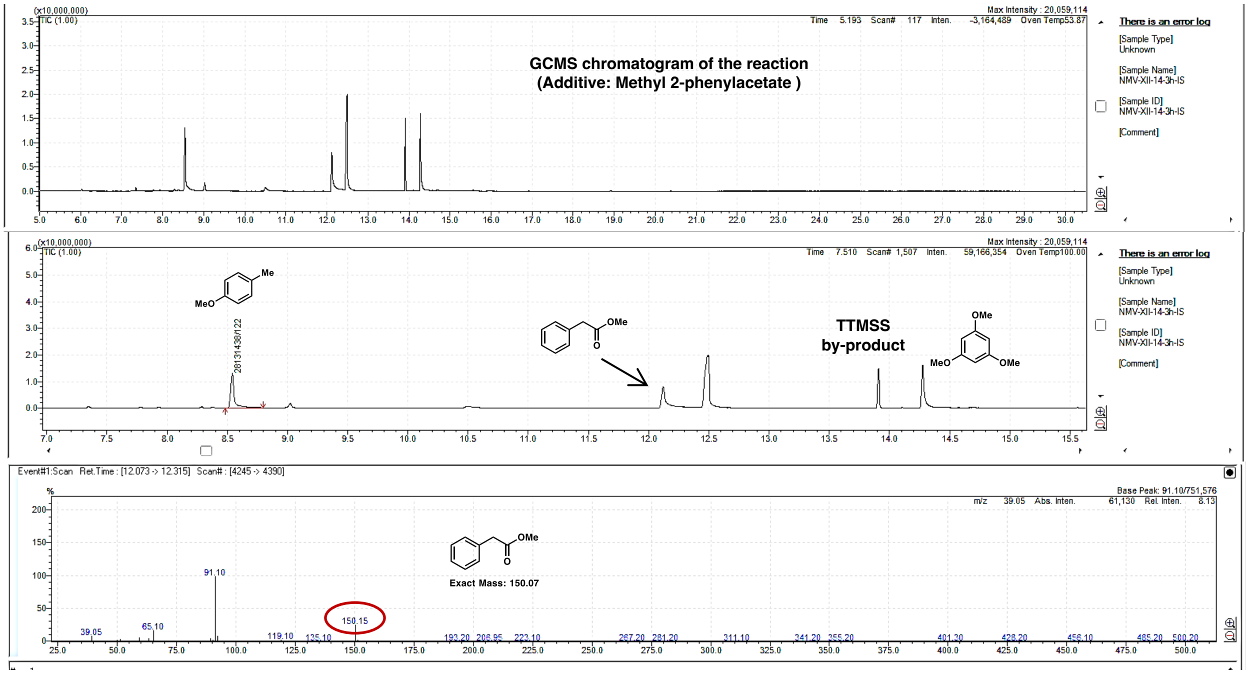

Figure *SI-29*: GCMS chromatogram and corresponding mass scan for additive peak

Table 4, Entry 3 (NMV-XII-017): Additive: Thioanisole (0.2 mmol)

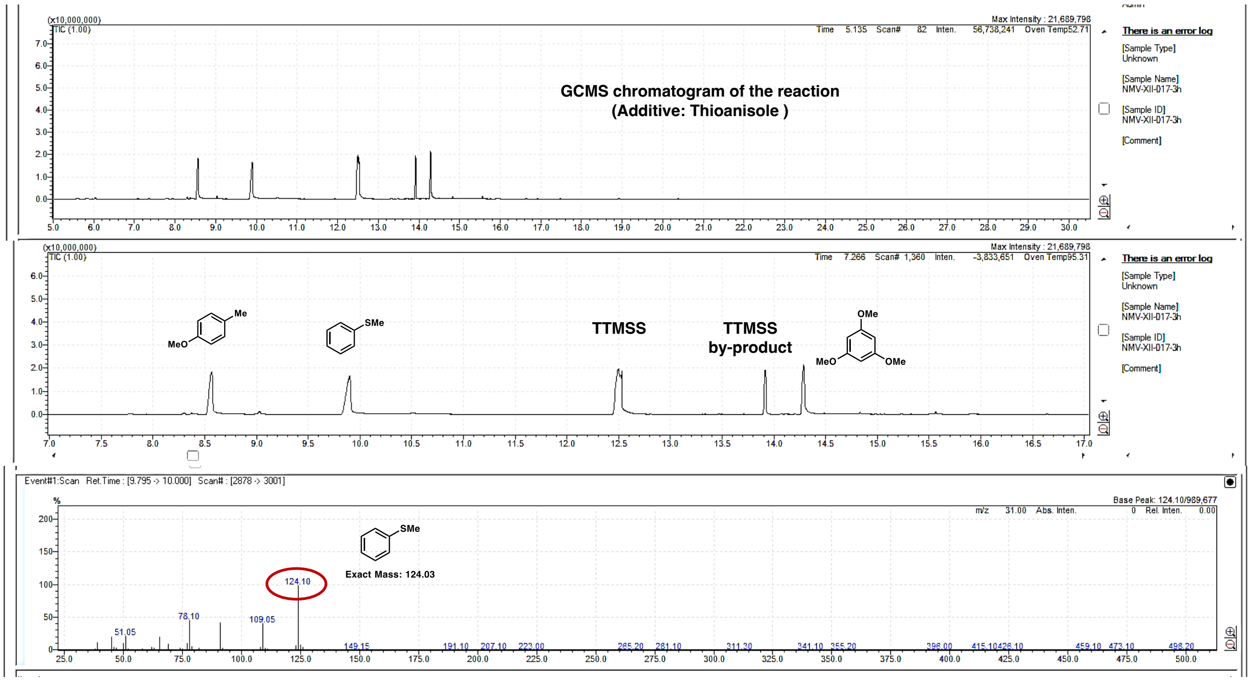

Figure *SI-30*: GCMS chromatogram and corresponding mass scan for additive peak

Table 4, Entry 4 (NMV-XII-016): Additive: 2-phenylethan-1-amine (0.2 mmol)

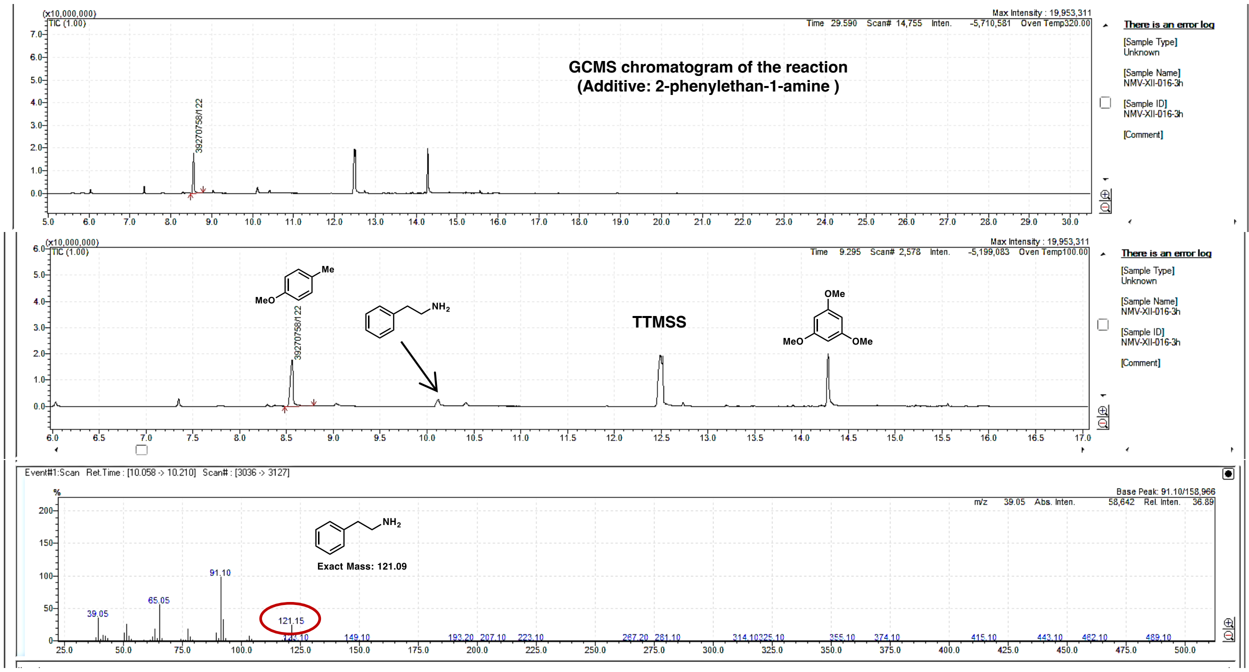

Figure *SI-31*: GCMS chromatogram and corresponding mass scan for additive peak

Table 4, Entry 5 (NMV-XII-031): Additive: Phenyl isocyanate (0.2 mmol)

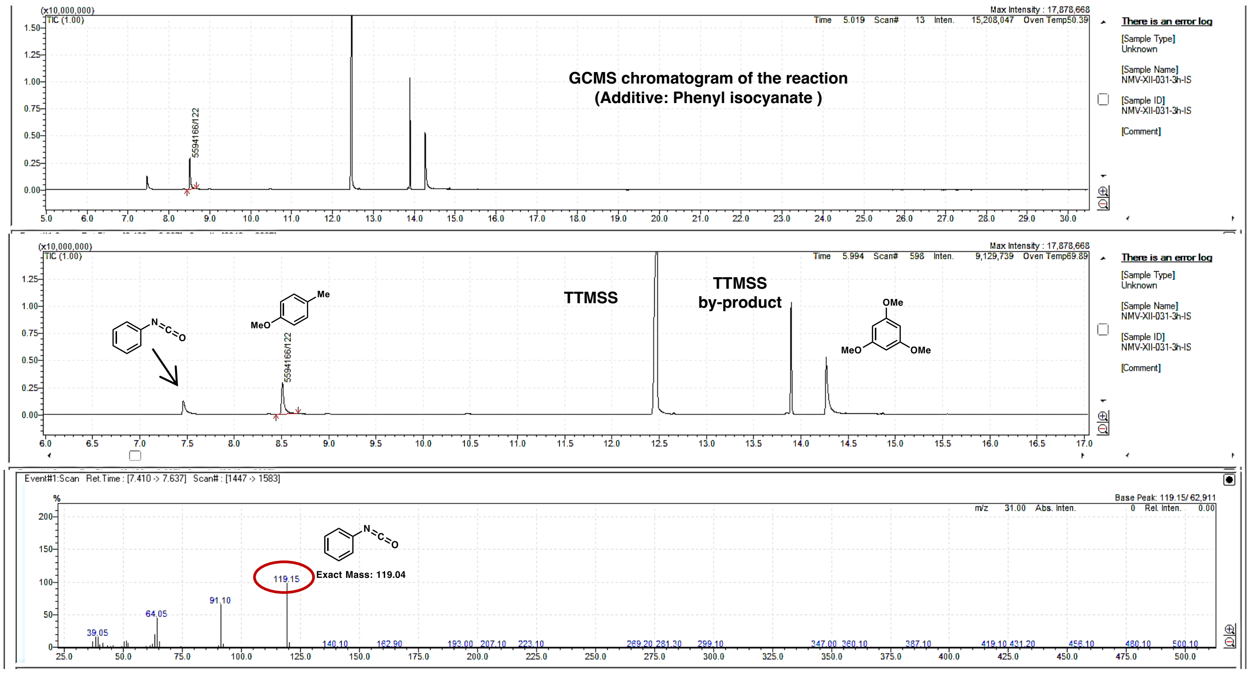

Figure *SI-32*: GCMS chromatogram and corresponding mass scan for additive peak

Table 4, Entry 6 (NMV-XII-030): Additive: 4-Bromo-2-fluoropyridine (0.2 mmol)

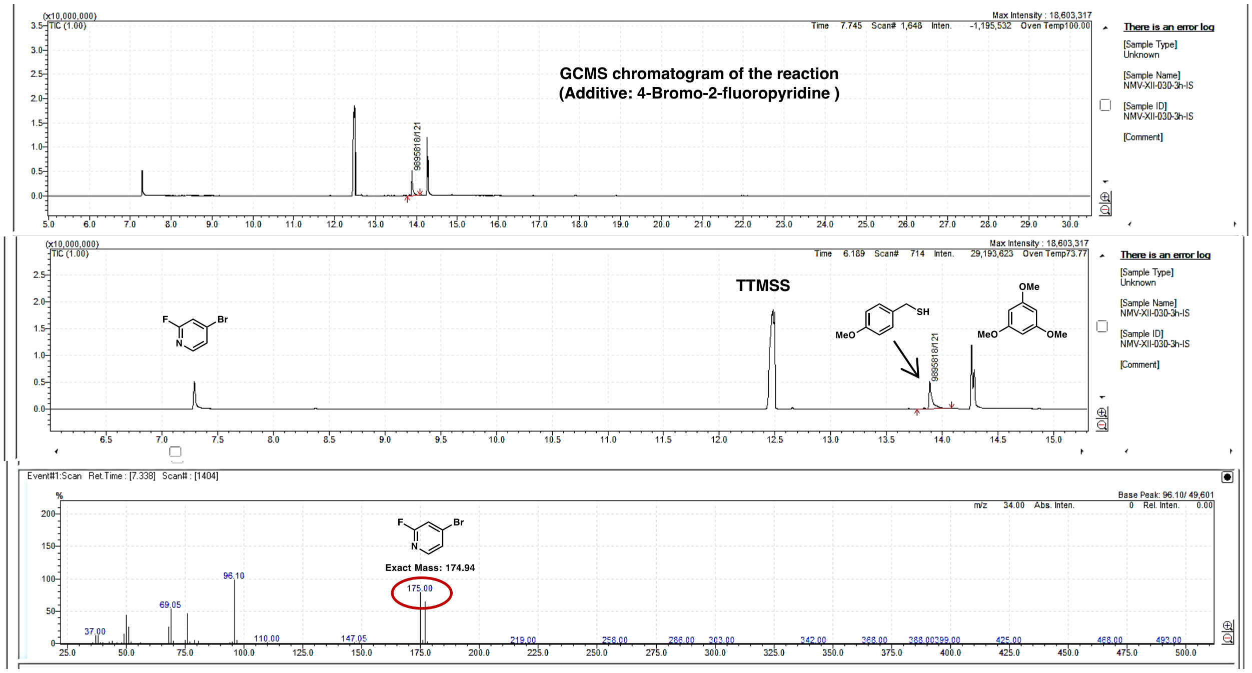

Figure *SI-33*: GCMS chromatogram and corresponding mass scan for additive peak

Table 4, Entry 7 (NMV-XII-029): Additive: 2-Chloropyridine (0.2 mmol)

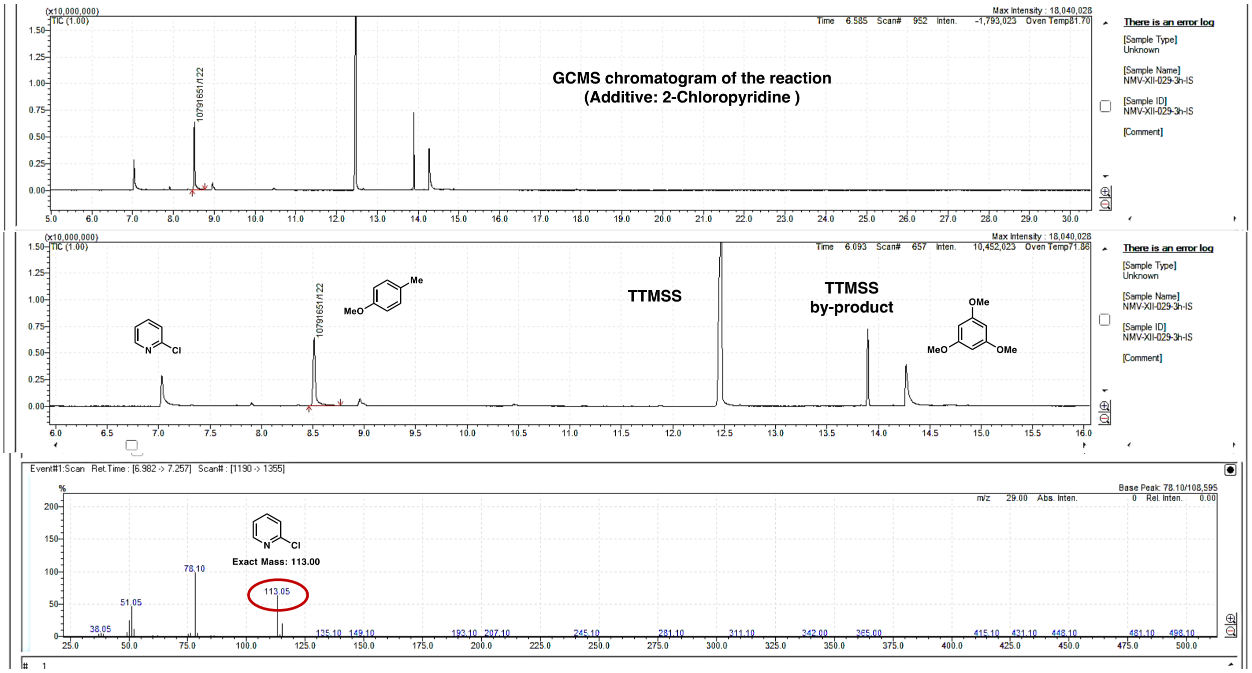

Figure *SI-34*: GCMS chromatogram and corresponding mass scan for additive peak

Table 4, Entry 8 (NMV-XII-035): Additive: 4-Bromobenzonitrile (0.2 mmol)

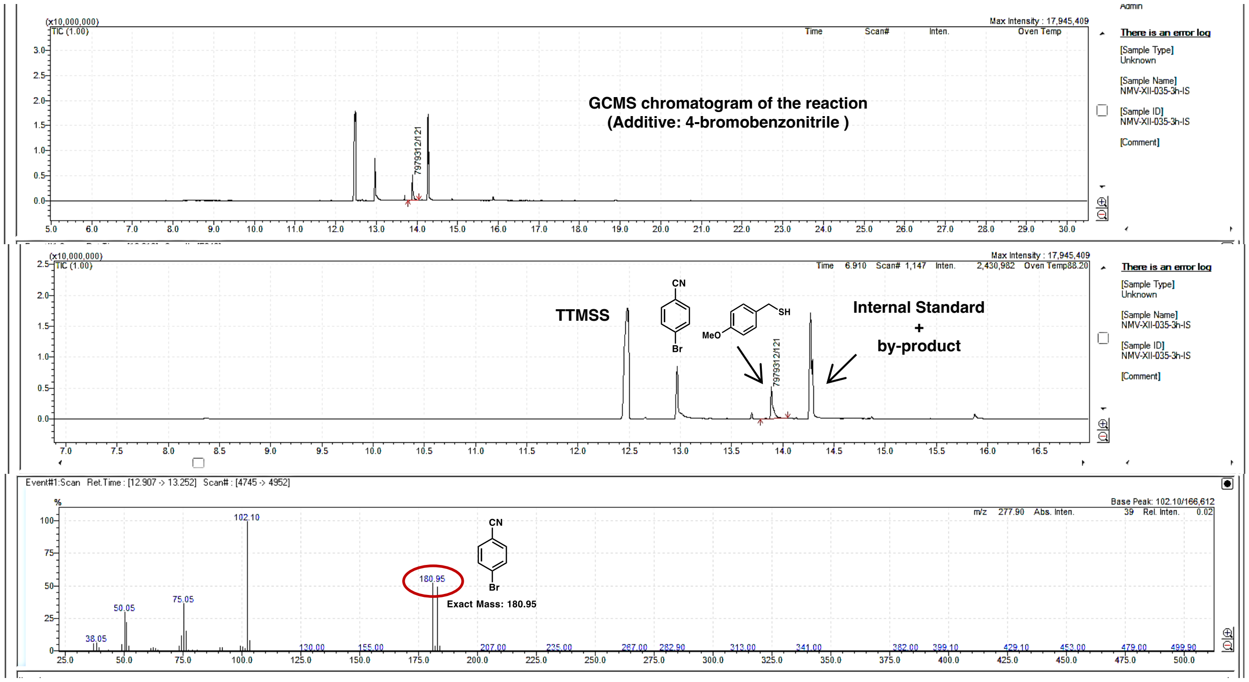

Figure *SI-35*: GCMS chromatogram and corresponding mass scan for additive peak

Table 4, Entry 9 (NMV-XII-033): Additive: *p*-Tolunitrile(0.2 mmol)

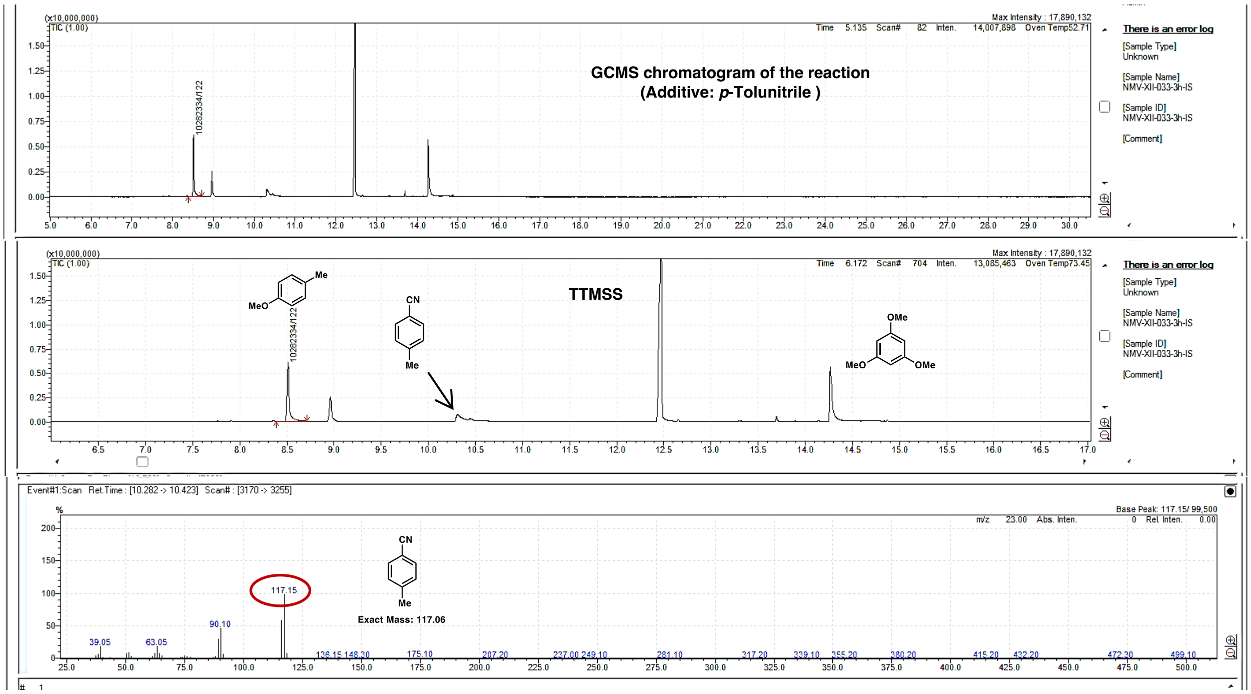

Figure *SI-36*: GCMS chromatogram and corresponding mass scan for additive peak

Table 4, Entry 10 (NMV-XII-034): Additive: 3,5-Dimethoxybenzonitrile (0.2 mmol)

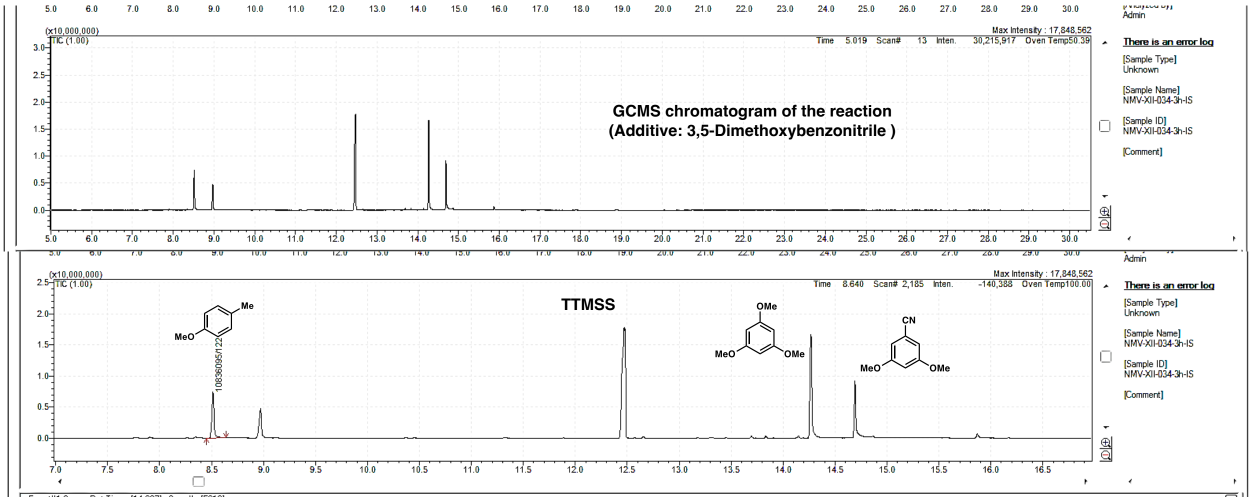

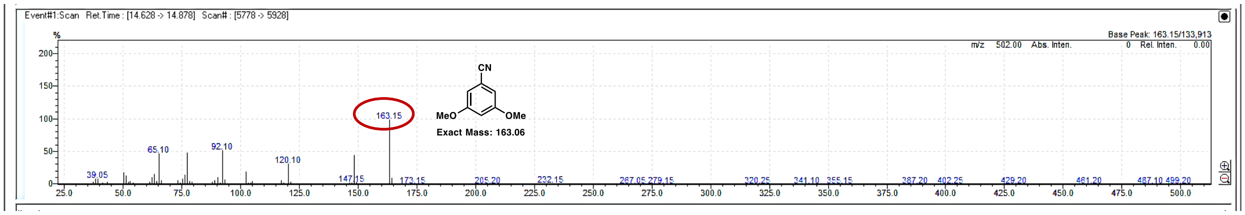

Figure *SI-37*: GCMS chromatogram and corresponding mass scan for additive peak

Table *SI-02:* Desulfurization outcome in the presence of reactive additives (by-products related to the additive were observed in these reactions).

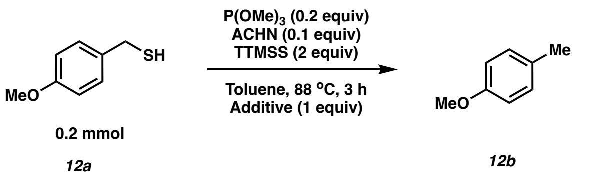

Entry[a]	Addtive	GCMS coversion - 12b formation (%)[b]	Unreacted *12a* (%)[b]
1	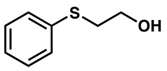	100	0
2	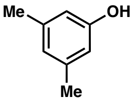	100	0
3	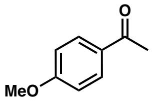	73	27
4	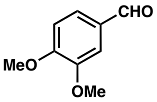	93	07

[a] Reaction conditions: (4-methoxyphenyl)methanethiol (0.2 mmol, 1 equiv), $P(OMe)_3$ (0.04 mmol, 0.2 equiv), ACHN (0.02 mmol, 0.1 equiv), TTMSS (0.4 mmol, 2 equiv), Toluene (0.05 M), 88 oC, 3 h [b] GCMS conversion was reported for product and starting material

Table *SI-02*, Entry 1 (NMV-XII-012): Additive: 2-(phenylthio)ethan-1-ol

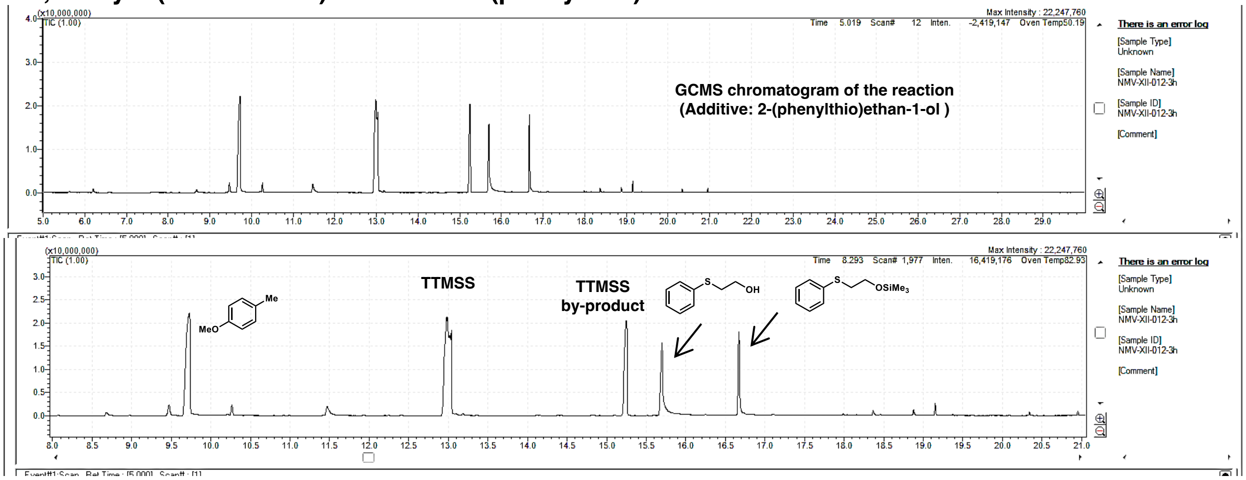

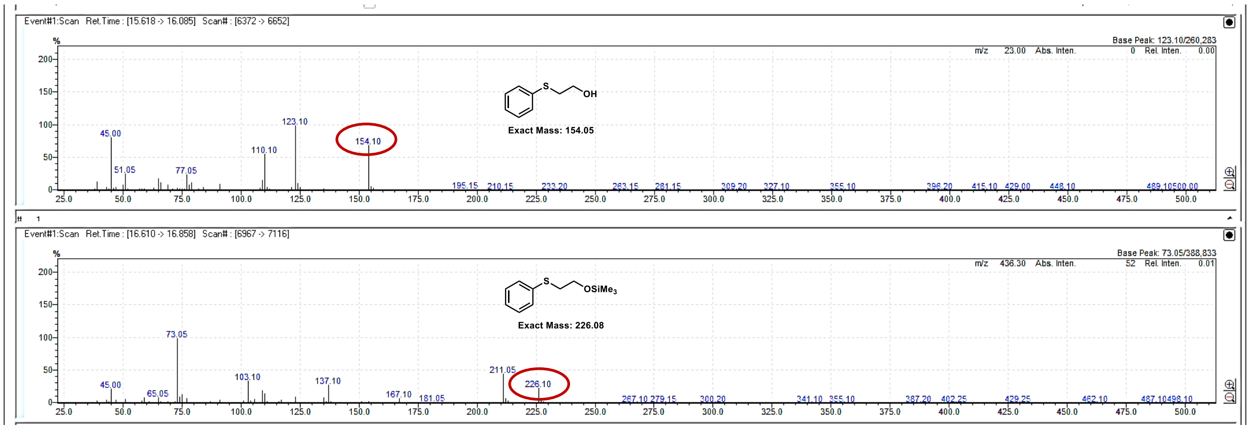

Figure *SI-38*: GCMS chromatogram and mass scan for additive and by-product peaks

Table *SI-02*, Entry 2 (NMV-XII-023): Additive: 3,5-dimethylphenol

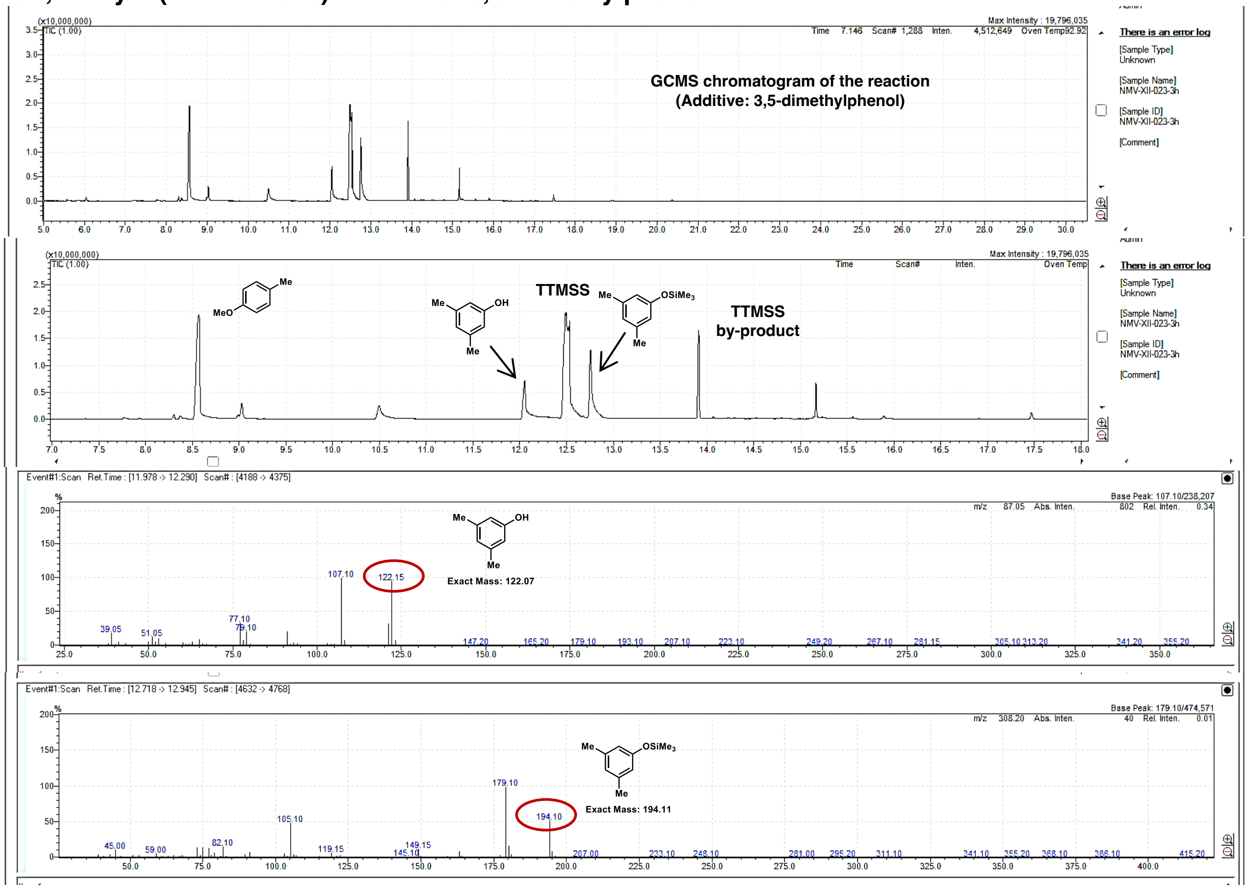

Figure *SI-39*: GCMS chromatogram and mass scan for additive and by-product peaks

Table *SI-02*, Entry 3 (NMV-XII-013): Additive: 1-(4-methoxyphenyl)ethan-1-one

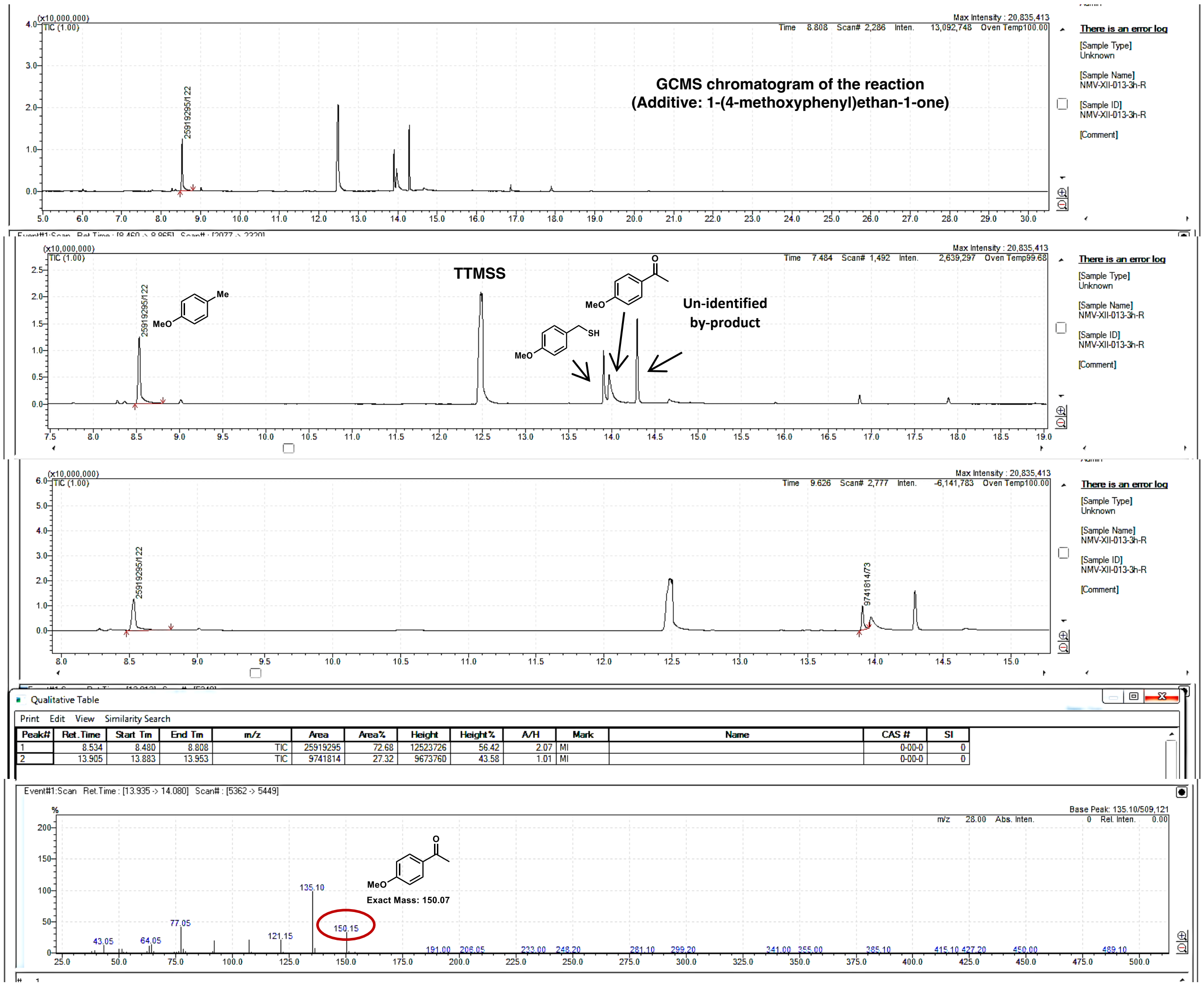

Figure *SI-40*: GCMS chromatogram and mass scan for additive peak

Table *SI-02*, Entry 4 (NMV-XII-015): Additive: 3,4-dimethoxybenzaldehyde

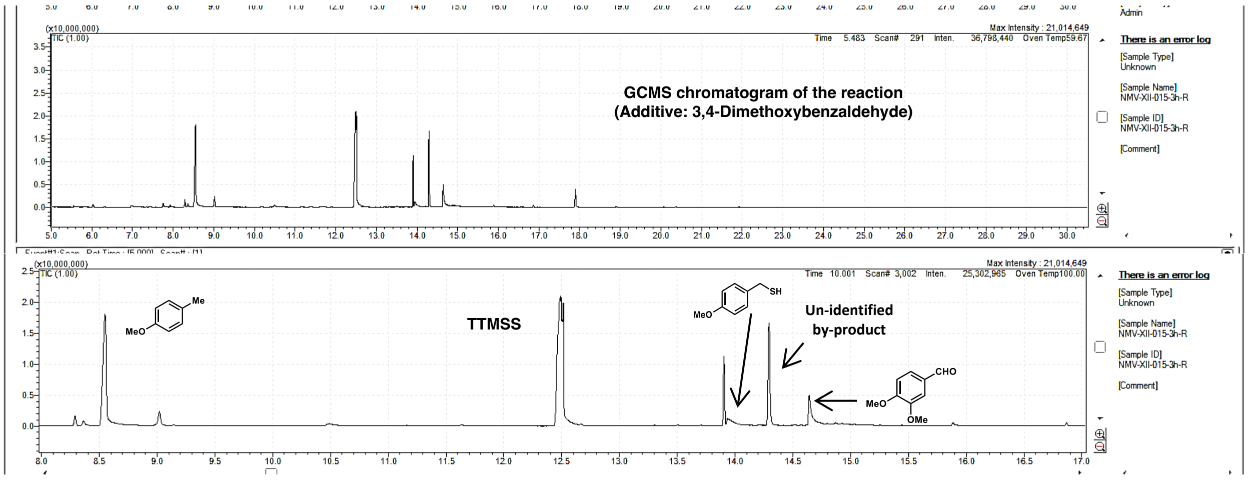

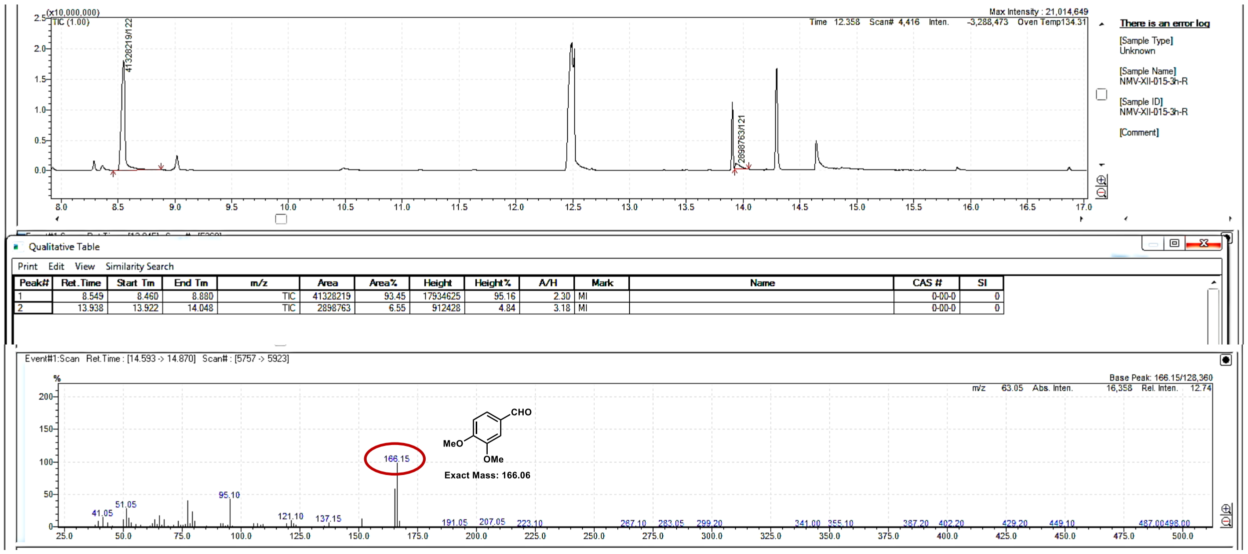

Figure *SI-41*: GCMS chromatogram and mass scan for additive peak

Gram Scale Reaction of Catalytic Desulfurization:

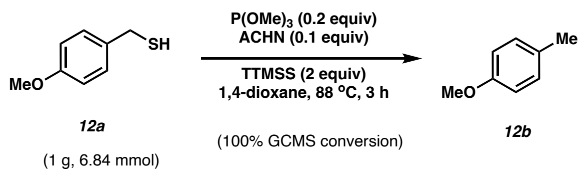

Scheme *SI-01*. Gram scale reaction of catalytic desulfurization

Procedure (NMV-XII-011): In a 250 ml round bottom flask, (4-methoxyphenyl) methanethiol ***SI-18*** (1g, 6.84 mmol, 1 equiv) was dissolved in 1,4-dioxane (130 mL, 0.05 M). TTMSS (4 mL, 12.97 mmol, 2 equiv) was added. The RB was sealed with rubber septa and protected with vinyl safety tape (see the image). This mixture was sparged with nitrogen for 20 minutes before adding $P(OMe)_3$ (0.153 mL, 1.297 mmol, 0.2 equiv) and ACHN (0.158 g, 0.648 mmol, 0.1 equiv). The reaction mixture was placed in preheated oil bath at 88 °C and the reaction mixture was stirred for three hours. The starting material was converted in 3 hours, which was determined by GCMS. Here we reported the GCMS conversion as the product distillation was difficult with other volatile reaction products.

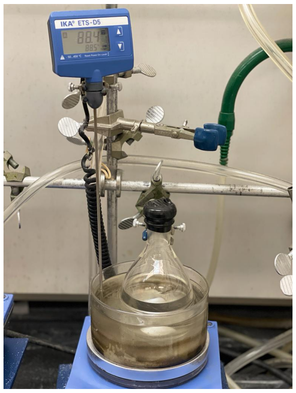

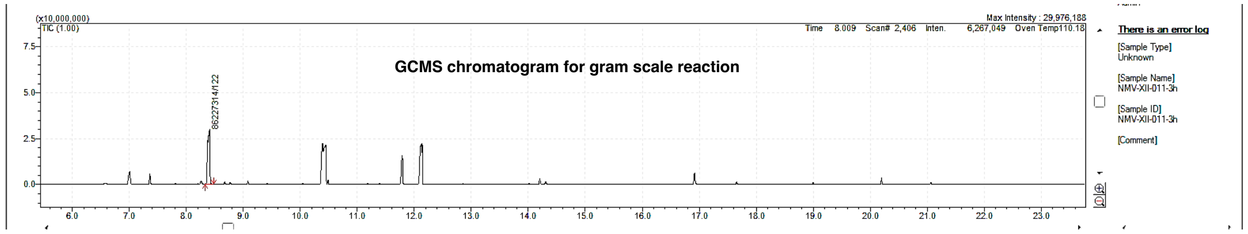

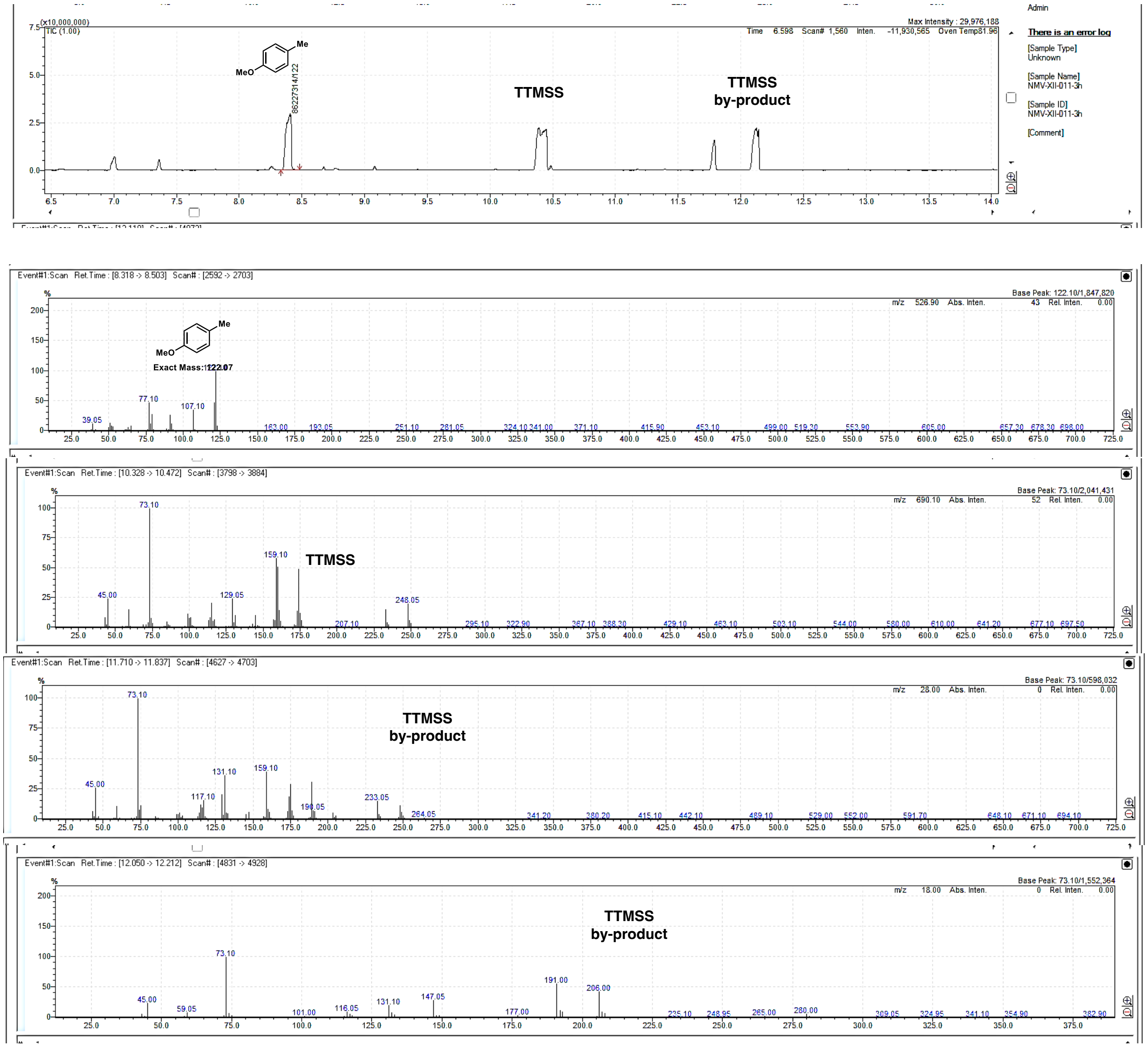

Figure *SI-42:* GCMS chromatogram and corresponding mass scans of the respective peaks

Experimental procedure and spectroscopic data of Scheme 1.

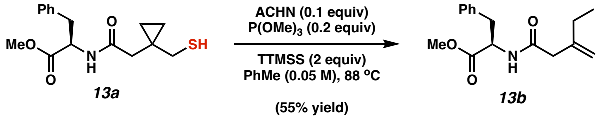

Methyl (3-methylenepentanoyl)-*L*-phenylalaninate (*13b*, RMI-XI-076). To a 20-mL microwave vial (with 14/20 septum under an atmosphere of argon), ***13a*** (0.08 g, 0.26 mmol, 1 equiv), PhMe (5.2 mL, 0.05 M), and TTMSS (0.16 mL, 0.52 mmol, 2 equiv) were added. The reaction mixture was purged with argon for few minutes then $P(OMe)_3$ (0.0061 mL, 0.052 mmol, 0.2 equiv) and ACHN (0.0063 g, 0.026 mmol, 0.1 equiv) were added. The reaction vial was sealed and placed in pre-heated silicon oil bath at 88 °C and stirred for 20 h. The reaction mixture was cooled to room temperature and concentrated under vacuum. The crude was purified using silica gel with an automated flash column chromatography system using 0-90% EtOAc in hexanes as an eluent to afford ***13b*** as off-white solid (0.0391 g, 55% yield). ^{1}H NMR (600 MHz, Chloroform-*d*) δ 7.30 – 7.17 (m, 3H), 7.14 – 7.03 (m, 2H), 6.14 (d, *J* = 8.0 Hz, 1H), 4.93 (q, *J* = 1.6 Hz, 1H), 4.88 – 4.82 (m, 2H), 3.71 (s, 3H), 3.13 (dd, *J* = 13.9, 5.7 Hz, 1H), 3.05 (dd, *J* = 13.9, 6.3 Hz, 1H), 2.95 (dd, *J* = 4.4, 1.0 Hz, 2H), 2.00 – 1.94 (m, 2H), 0.97 (t, *J* = 7.4 Hz, 3H). ^{13}C NMR (151 MHz, Chloroform-*d*) δ 171.97, 170.03, 145.48, 135.75, 129.16, 128.55, 127.12, 113.50, 77.23, 77.02, 76.81, 52.85, 52.28, 44.77, 37.78, 28.63, 11.90. HRMS $[M+H]^+$ calc'd for $[C_{16}H_{21}NO_3+H]^+$: *m/z* 276.1594, found 276.1594.

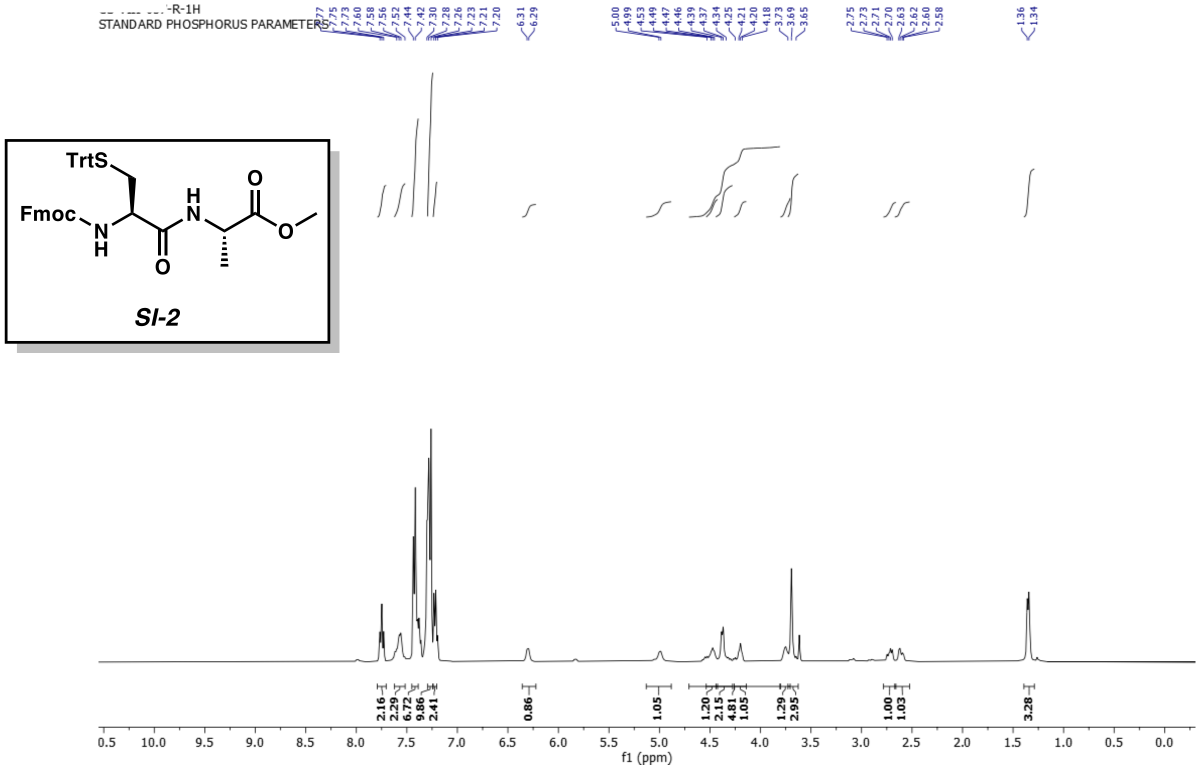

Figure *SI.43*. ^{1}H-NMR (499 MHz, $CDCl_3$) of compound ***SI-2***.

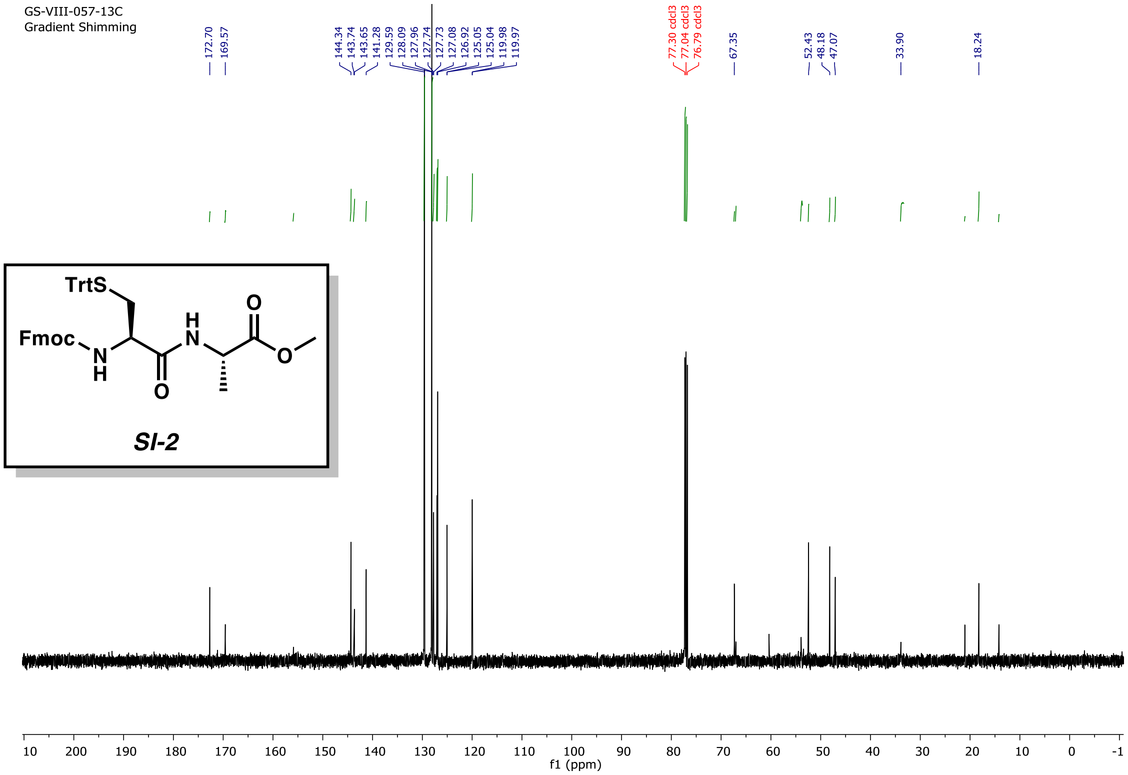

Figure *SI.44*. ^{13}C NMR (126 MHz, $CDCl_3$) of compound ***SI-2***.

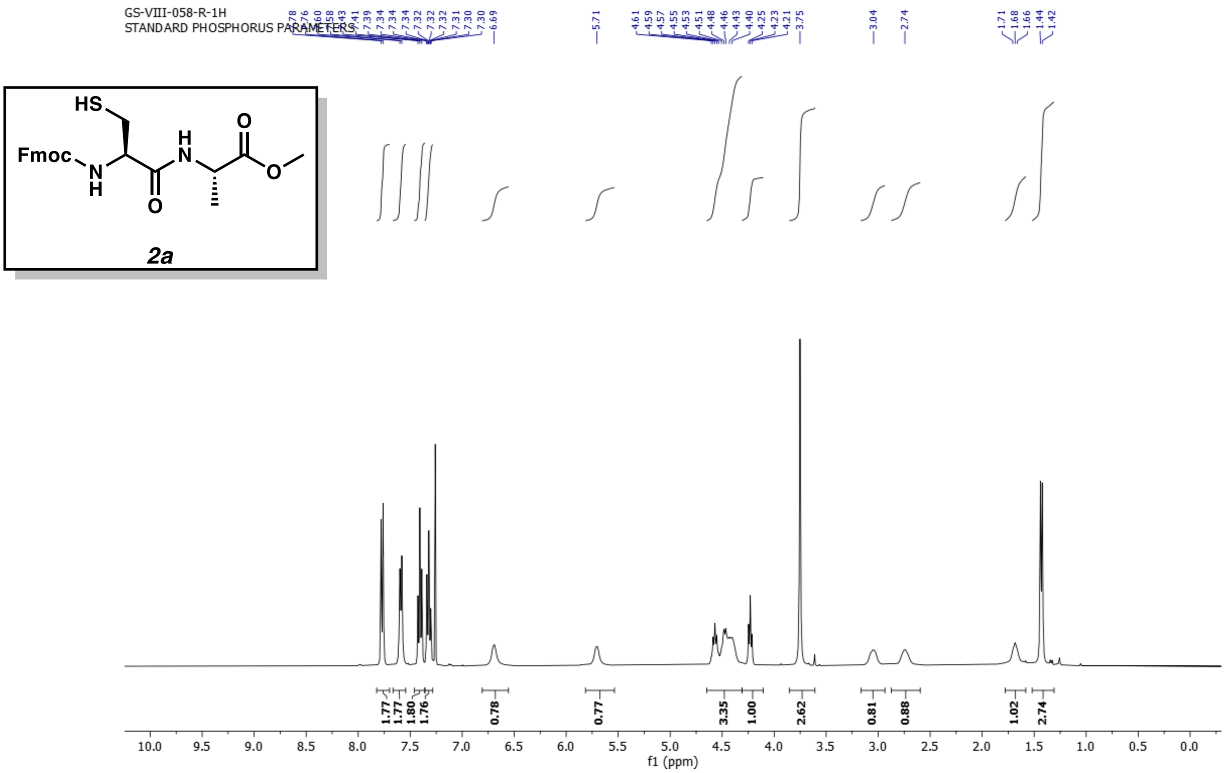

Figure *SI.45*. ^{1}H-NMR (499 MHz, $CDCl_3$) of compound ***2a*.**

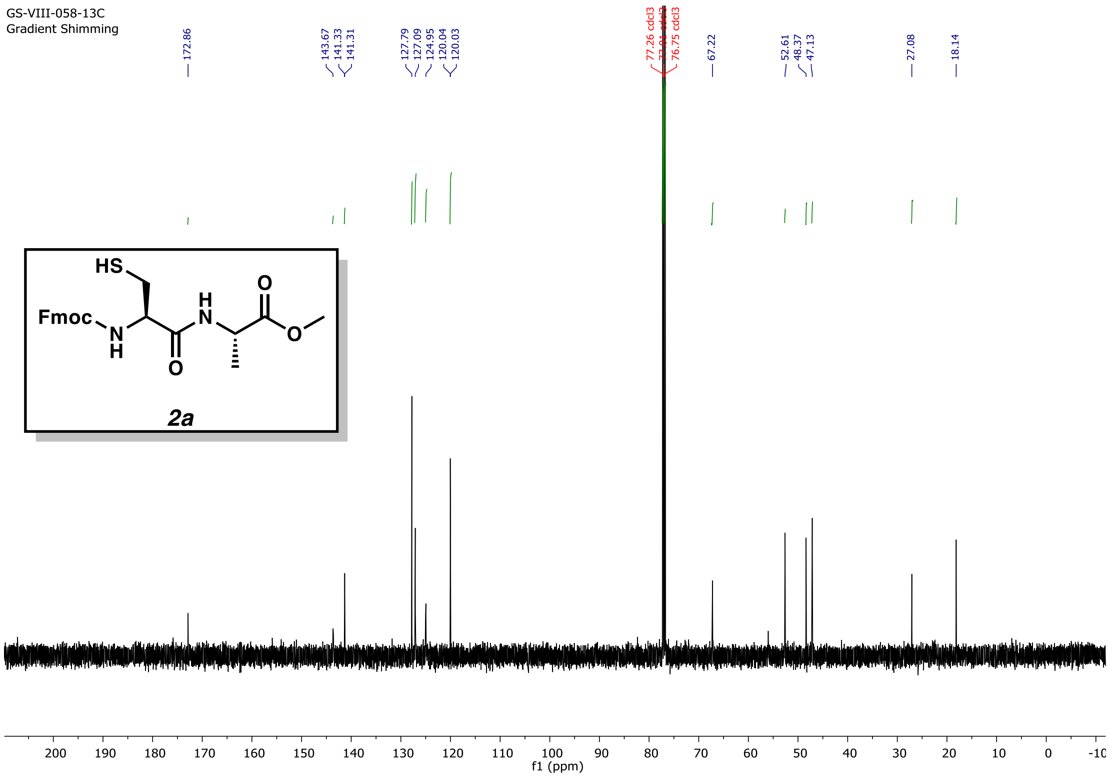

Figure *SI.46*. ^{13}C NMR (126 MHz, $CDCl_3$) of compound ***2a*.**

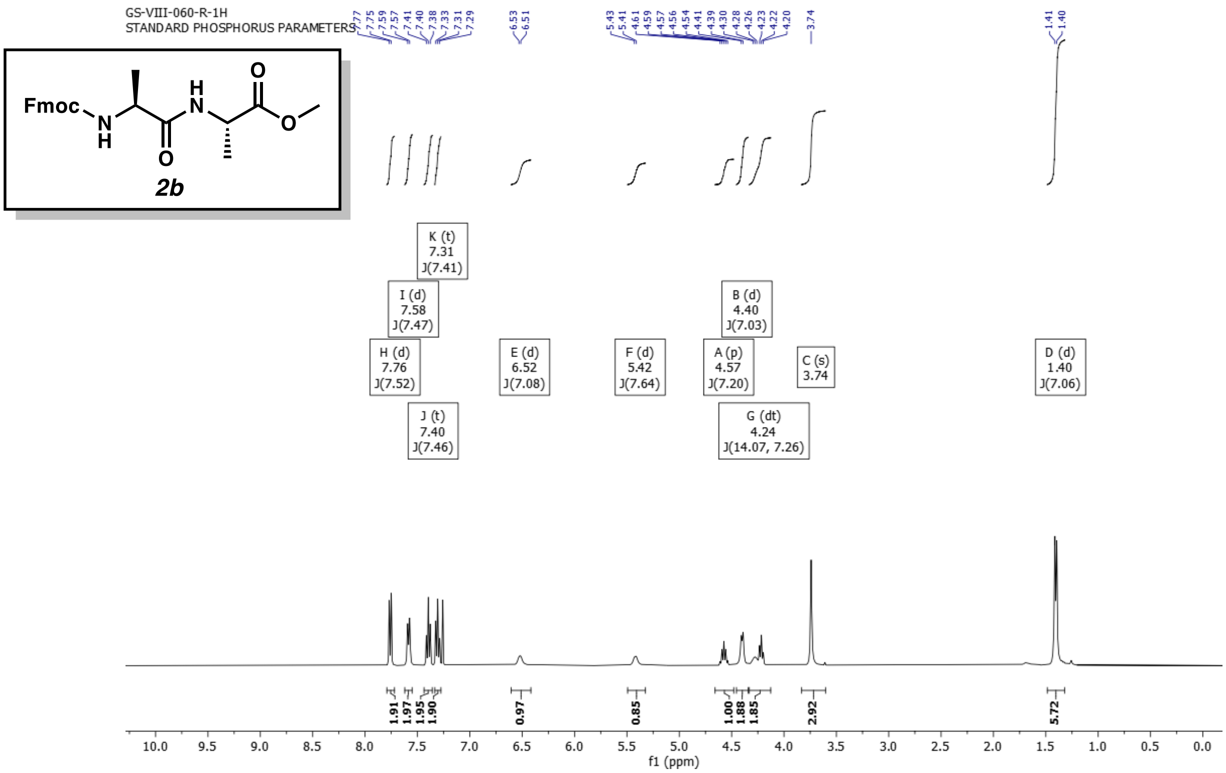

Figure *SI.47*. ^{1}H-NMR (600 MHz, $CDCl_3$) of compound ***2b*.**

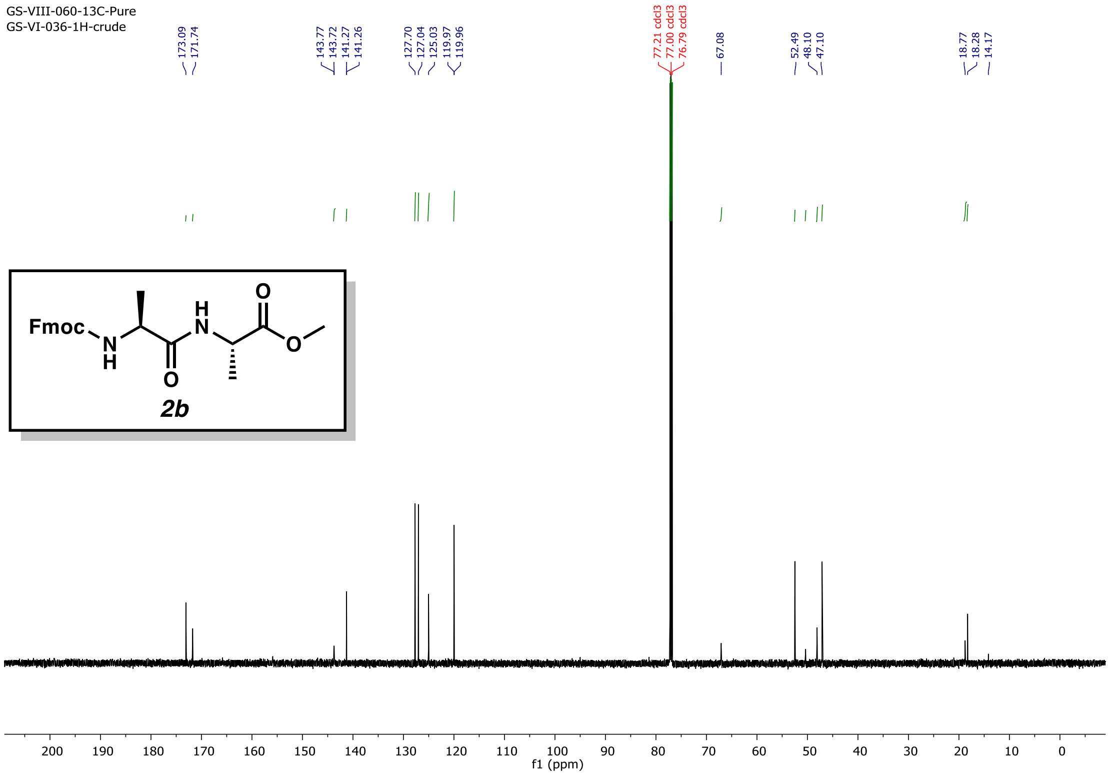

Figure *SI.48*. ^{13}C NMR (151 MHz, $CDCl_3$) of compound ***2b*.**

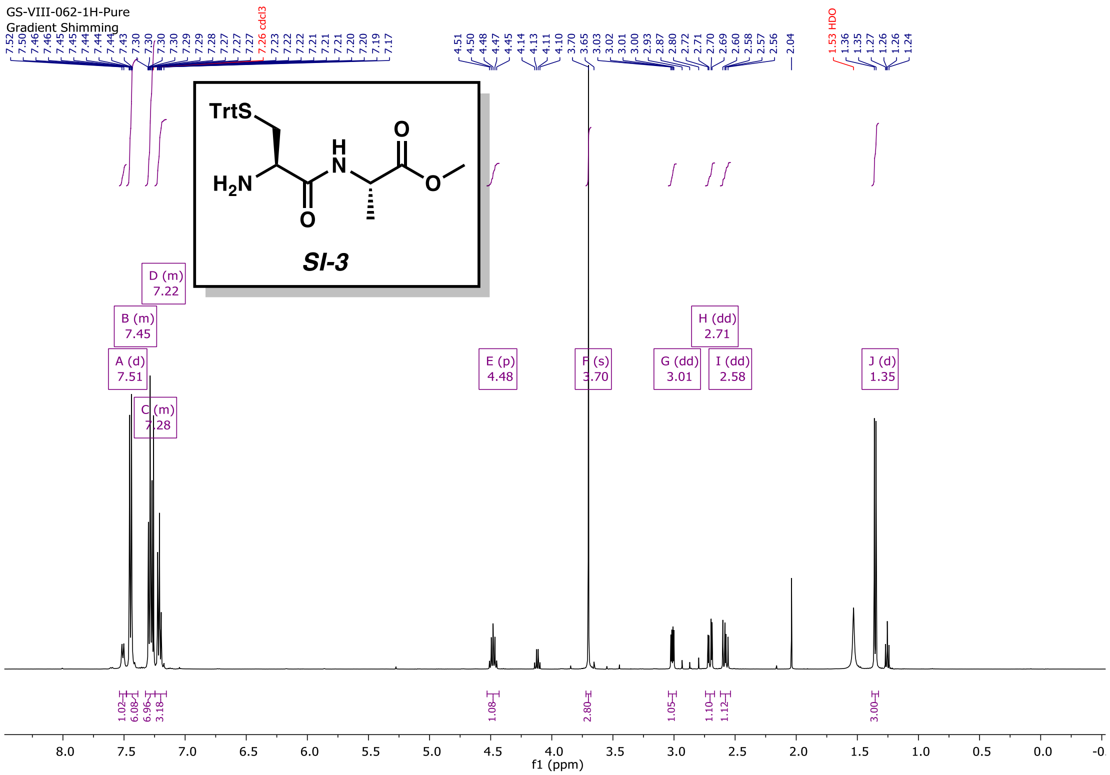

Figure *SI.49*. ^{1}H-NMR (499 MHz, $CDCl_3$) of compound ***SI-3*.**

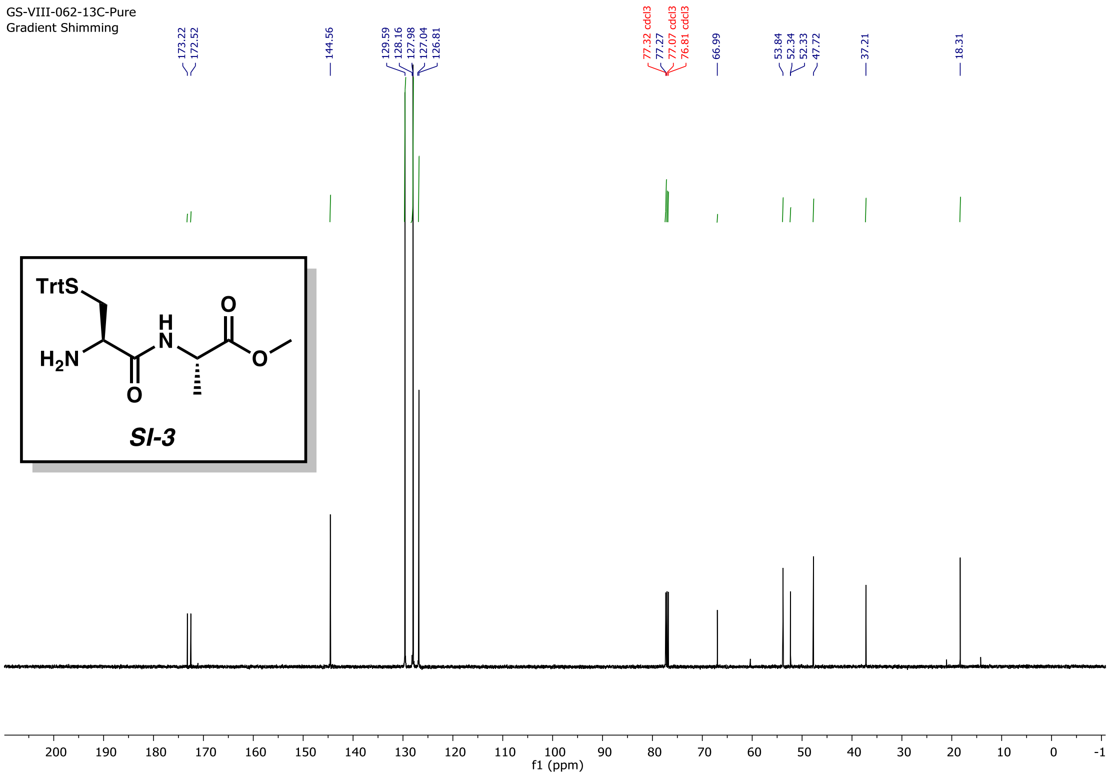

Figure *SI.50*. ^{13}C NMR (126 MHz, $CDCl_3$) of compound ***SI-3*.**

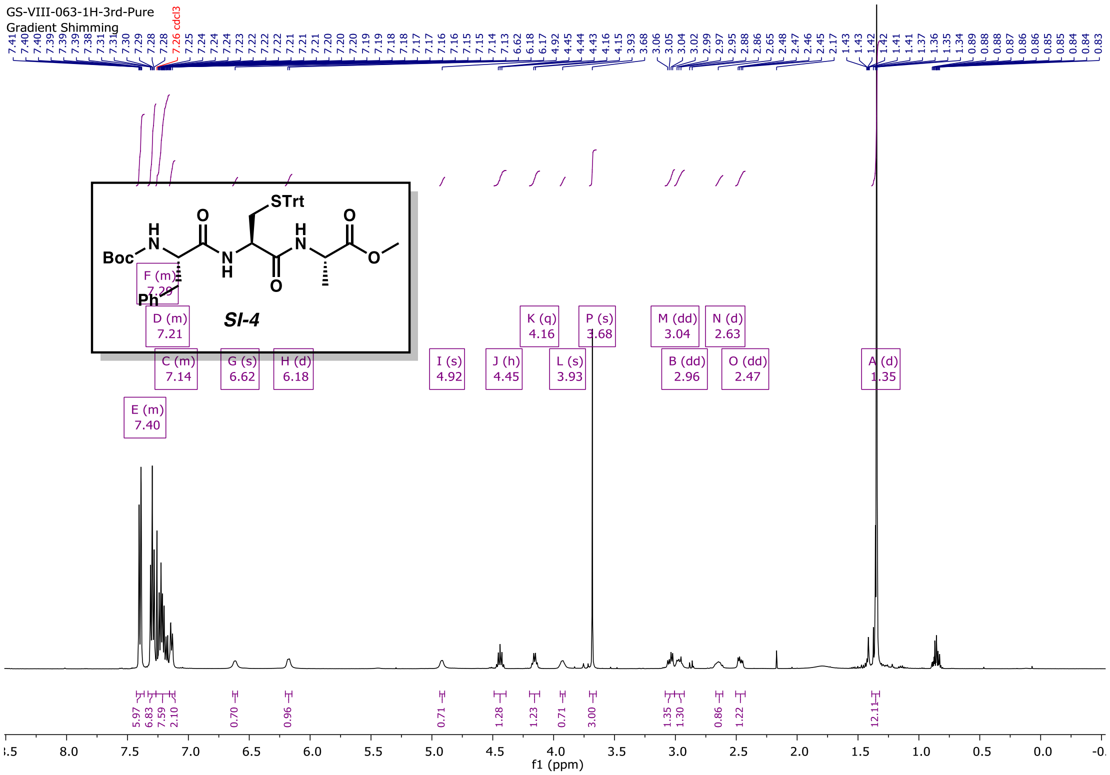

Figure *SI.51*. ^{1}H-NMR (499 MHz, $CDCl_3$) of compound ***SI-4*.**

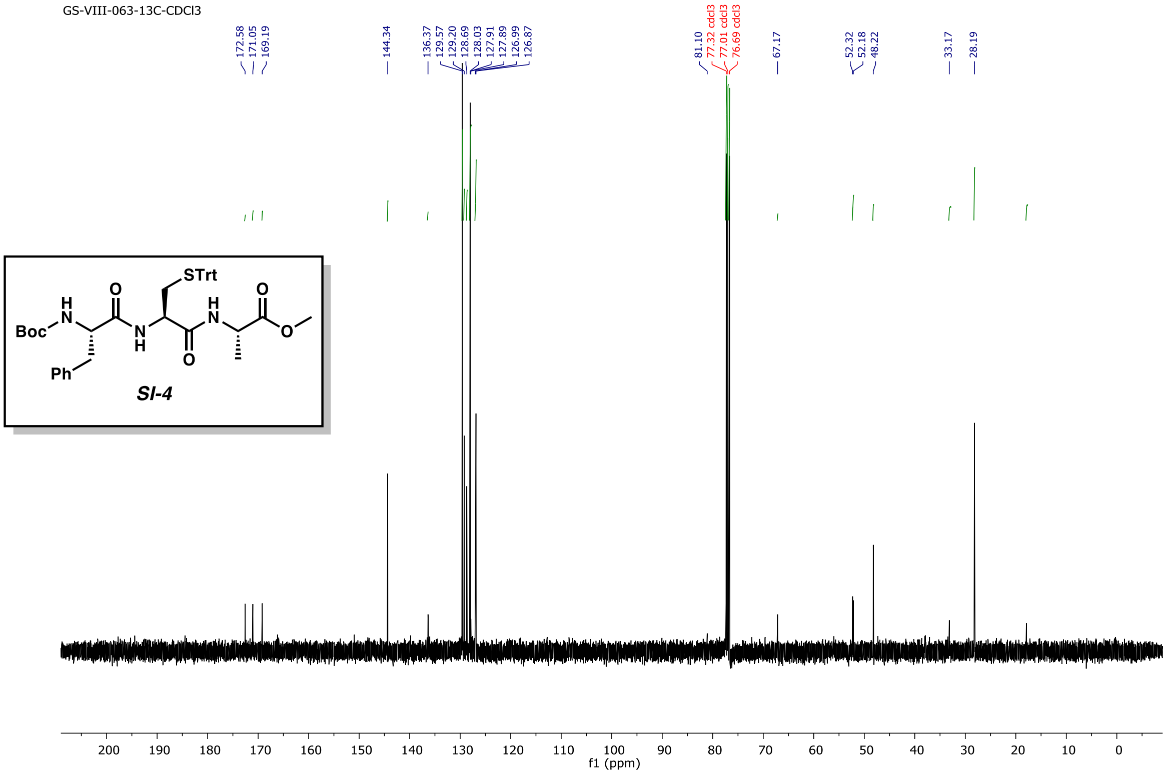

Figure *SI.52*. ^{13}C NMR (101 MHz, $CDCl_3$) of compound ***SI-4*.**

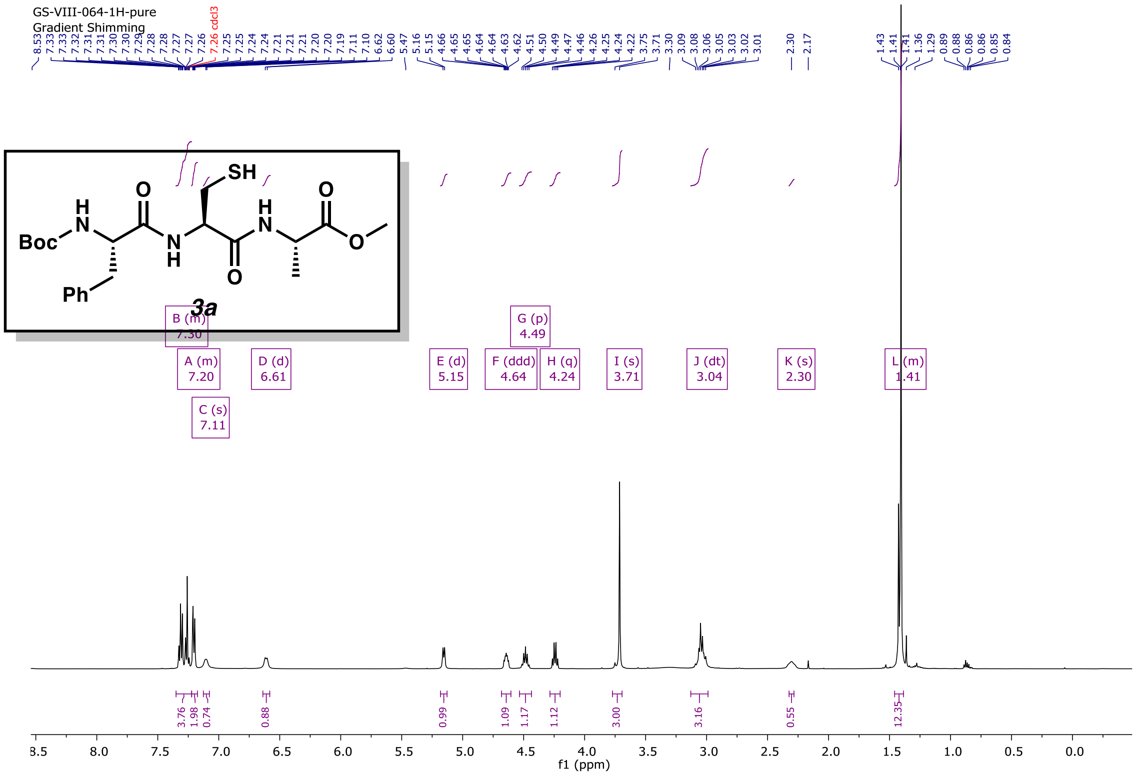

Figure *SI.53*. ^{1}H-NMR (499 MHz, $CDCl_3$) of compound ***3a***.

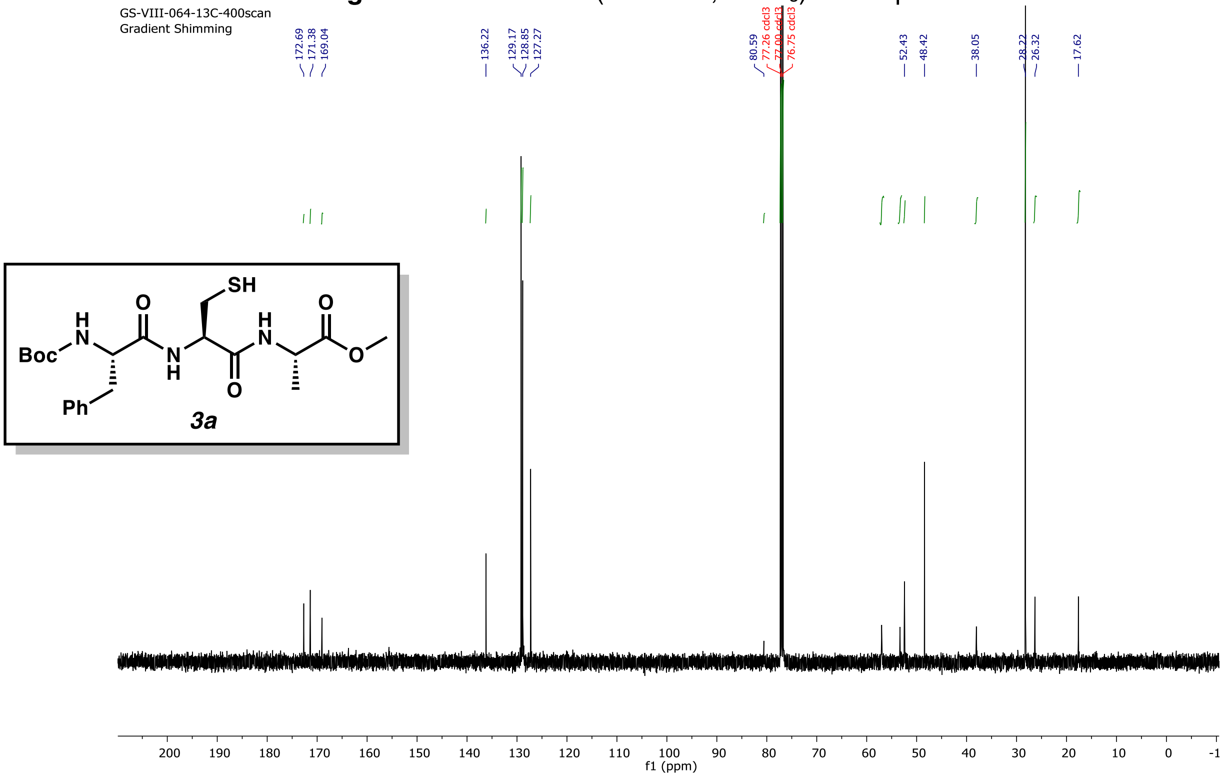

Figure *SI.54*. ^{13}C NMR (126 MHz, $CDCl_3$) of compound ***3a***.

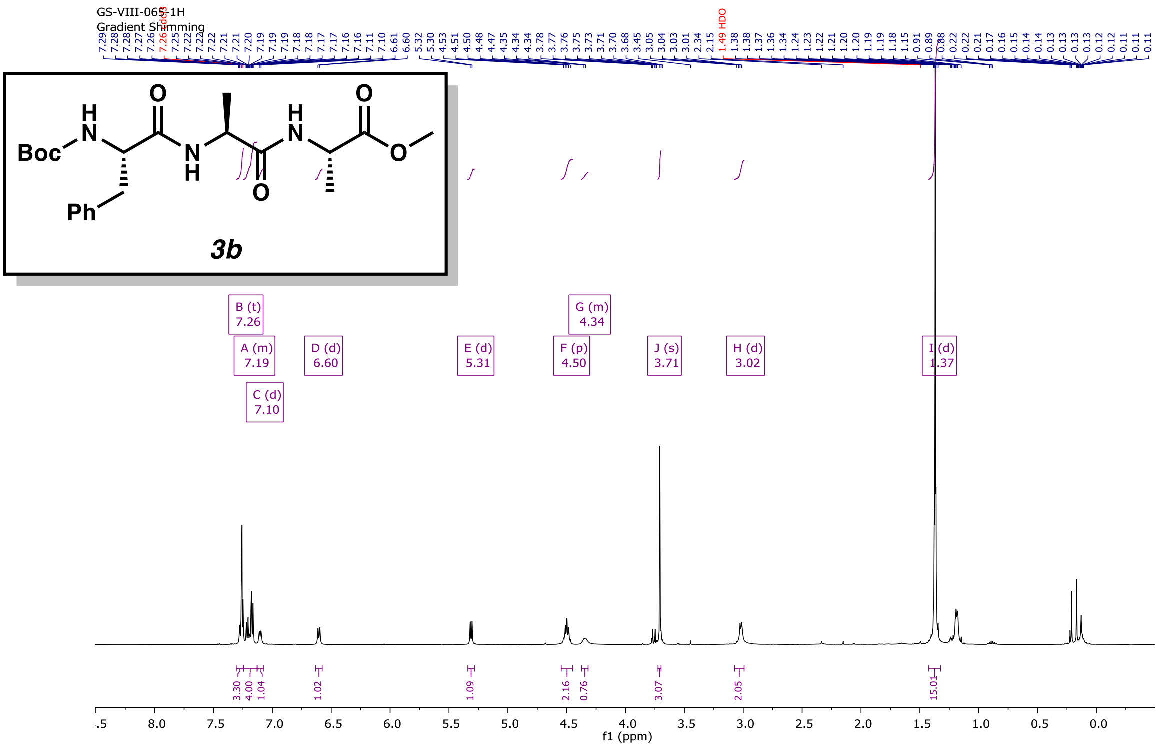

Figure *SI.55*. ^{1}H-NMR (499 MHz, $CDCl_3$) of compound ***3b*.**

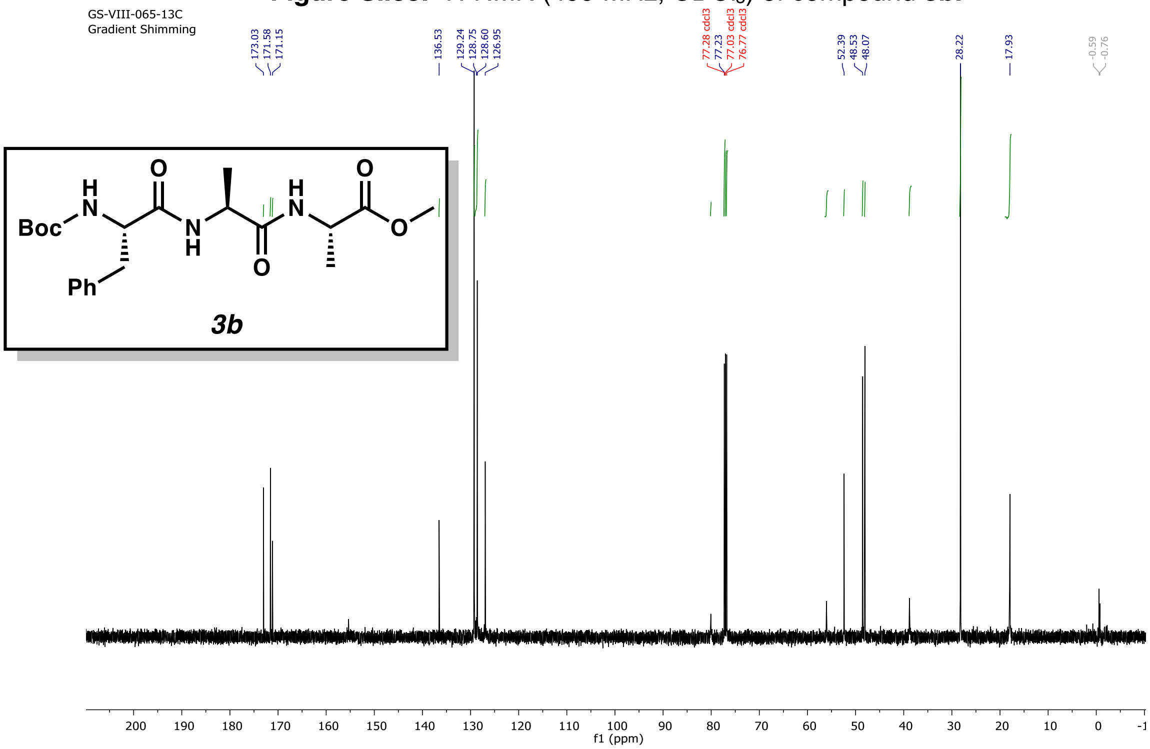

Figure *SI.56*. ^{13}C NMR (126 MHz, $CDCl_3$) of compound ***3b*.**

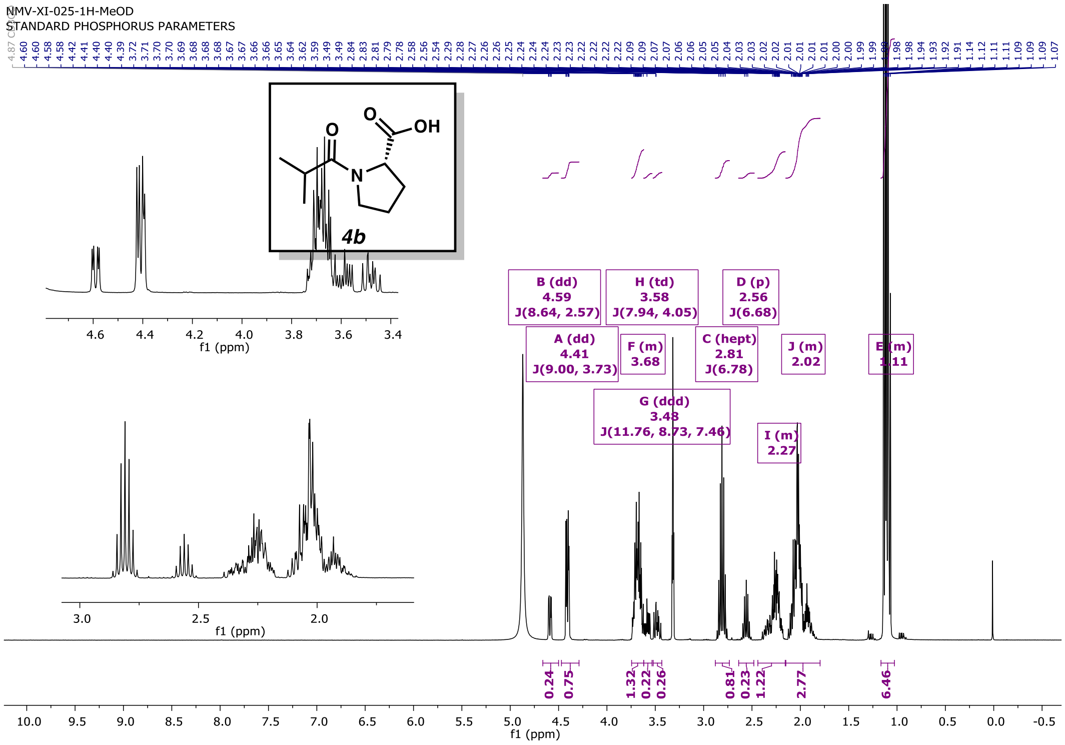

Figure *SI.57*. ^{1}H-NMR (400 MHz, Methanol-d_4) of compound ***4b***.

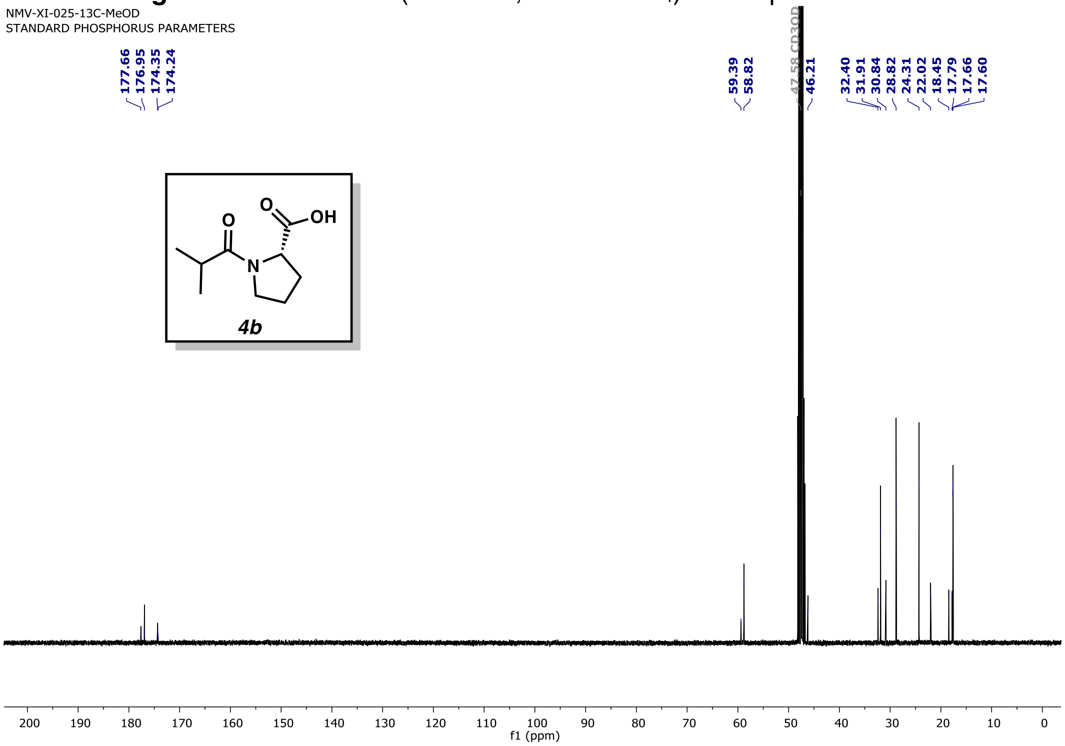

Figure *SI.58*. ^{13}C NMR (101 MHz, Methanol-d_4) of compound ***4b***.

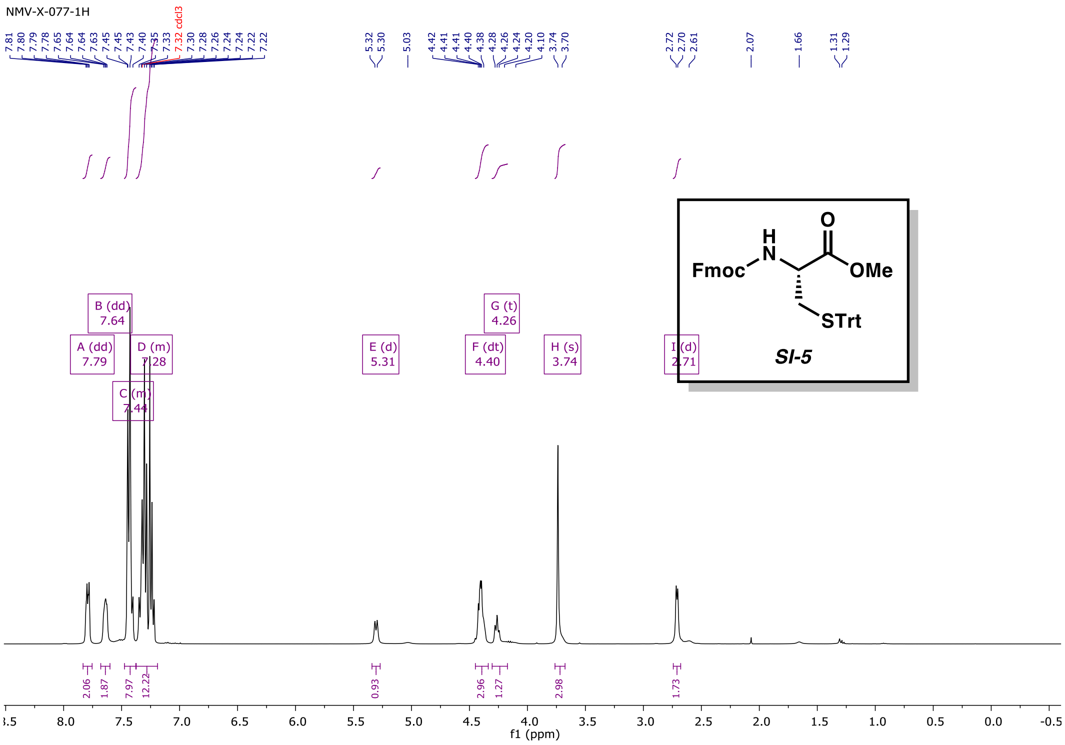

Figure *SI.59*. ^{1}H NMR (400 MHz, $CDCl_3$) of compound ***SI-5***.

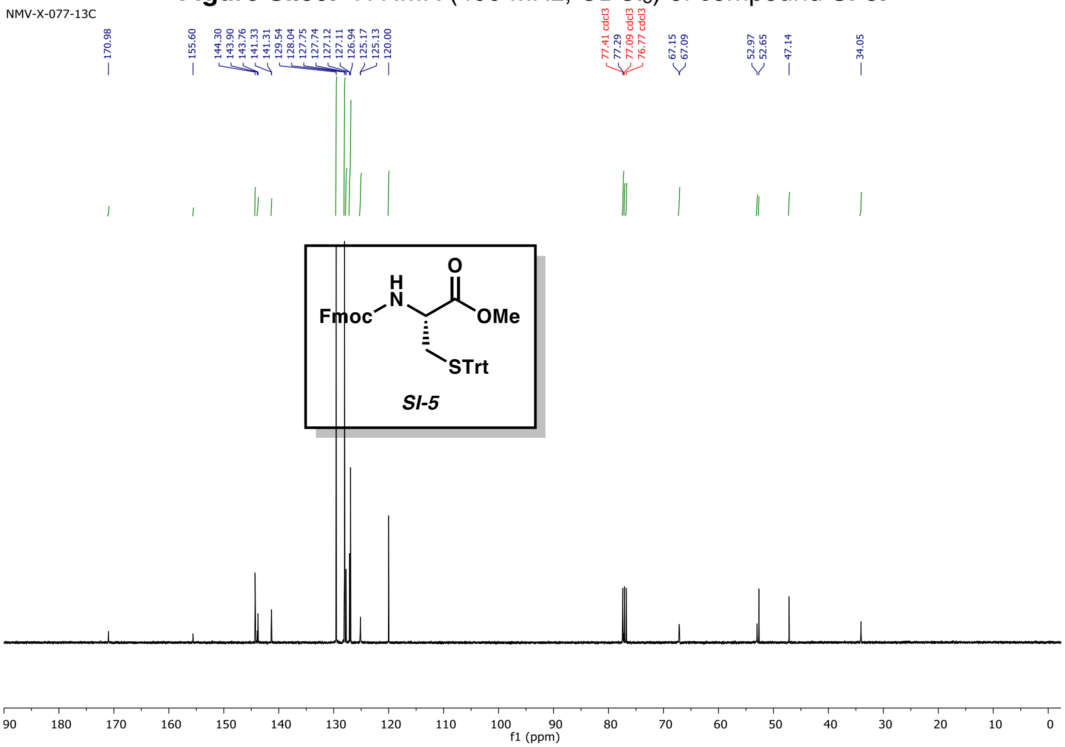

Figure *SI.60*. ^{13}C NMR (101 MHz, $CDCl_3$) of compound ***SI-5***.

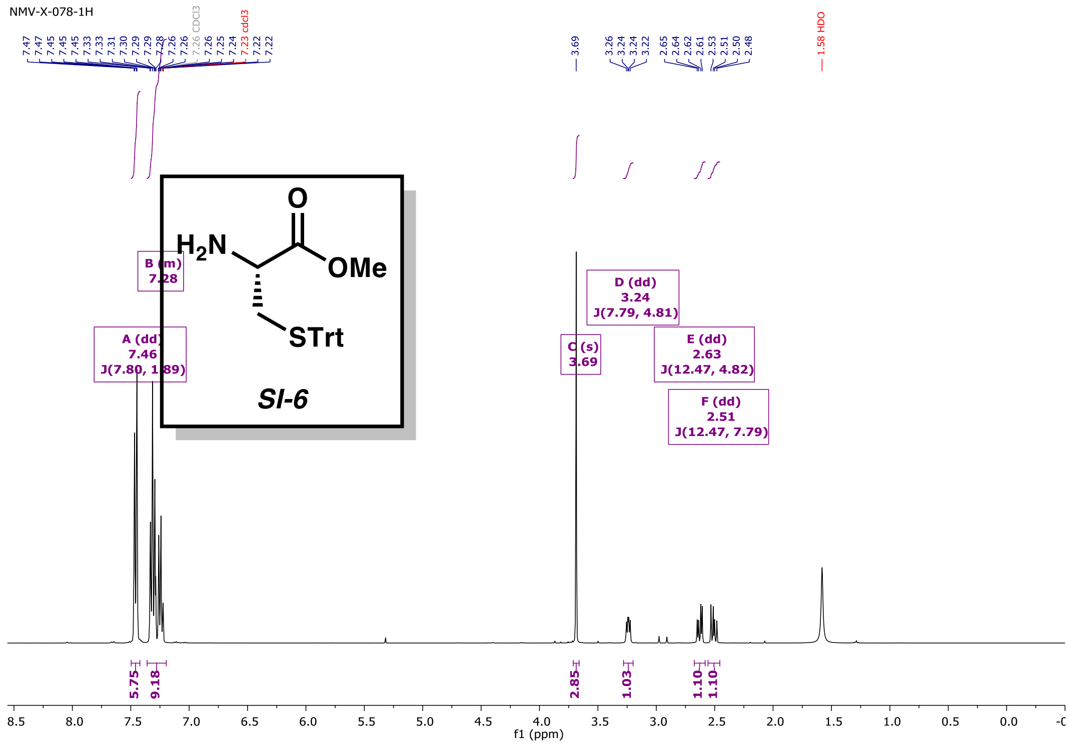

Figure *SI.61*. ^{1}H NMR (400 MHz, $CDCl_3$) of compound ***SI-6*.**

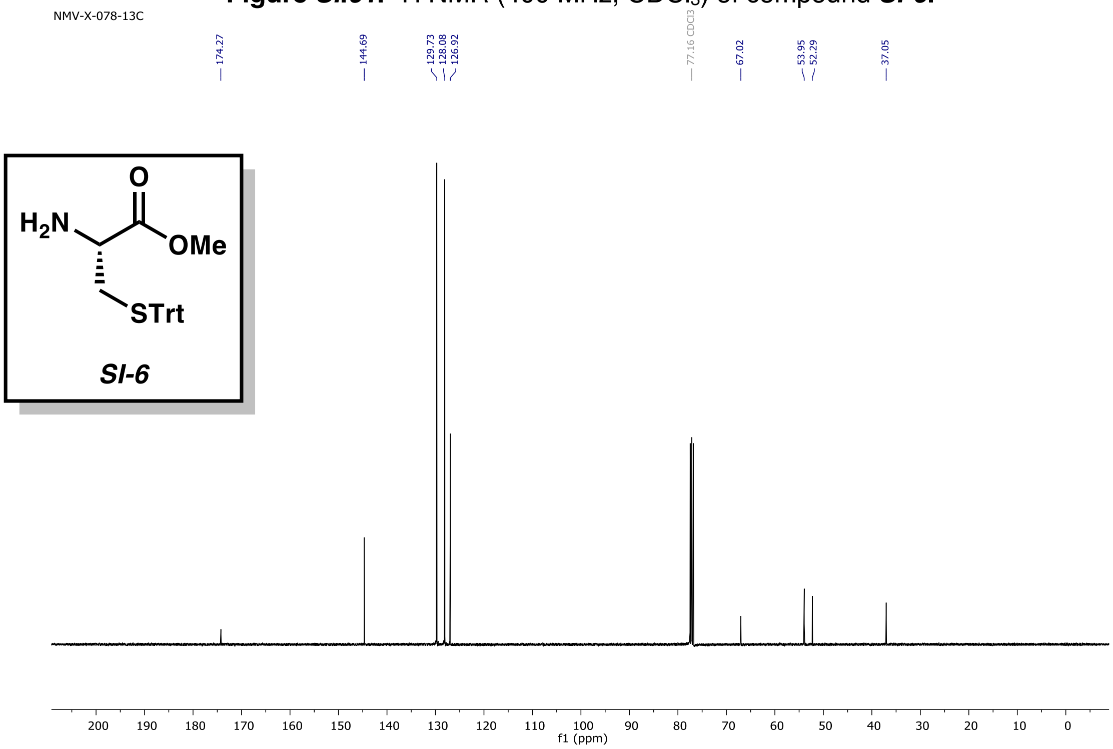

Figure *SI.62*. ^{13}C NMR (101 MHz, $CDCl_3$) of compound ***SI-6*.**

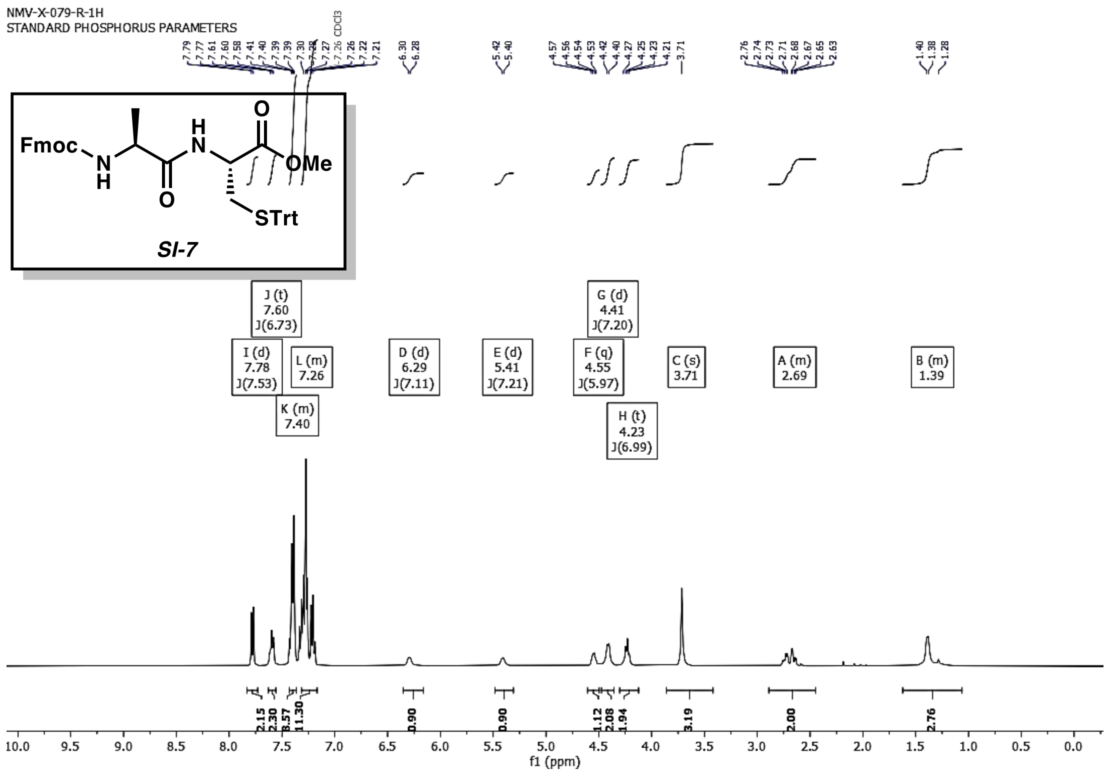

Figure *SI.63*. ^{1}H NMR (400 MHz, $CDCl_3$) of compound ***SI-7*.**

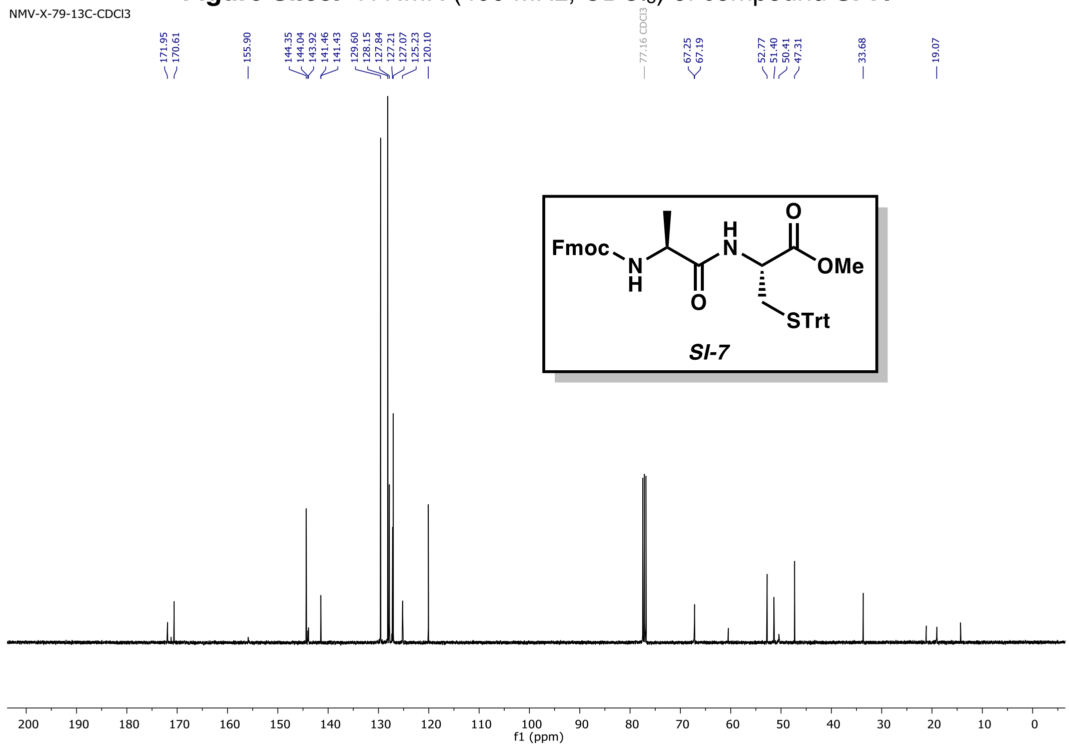

Figure *SI.64*. ^{13}C NMR (101 MHz, $CDCl_3$) of compound ***SI-7*.**

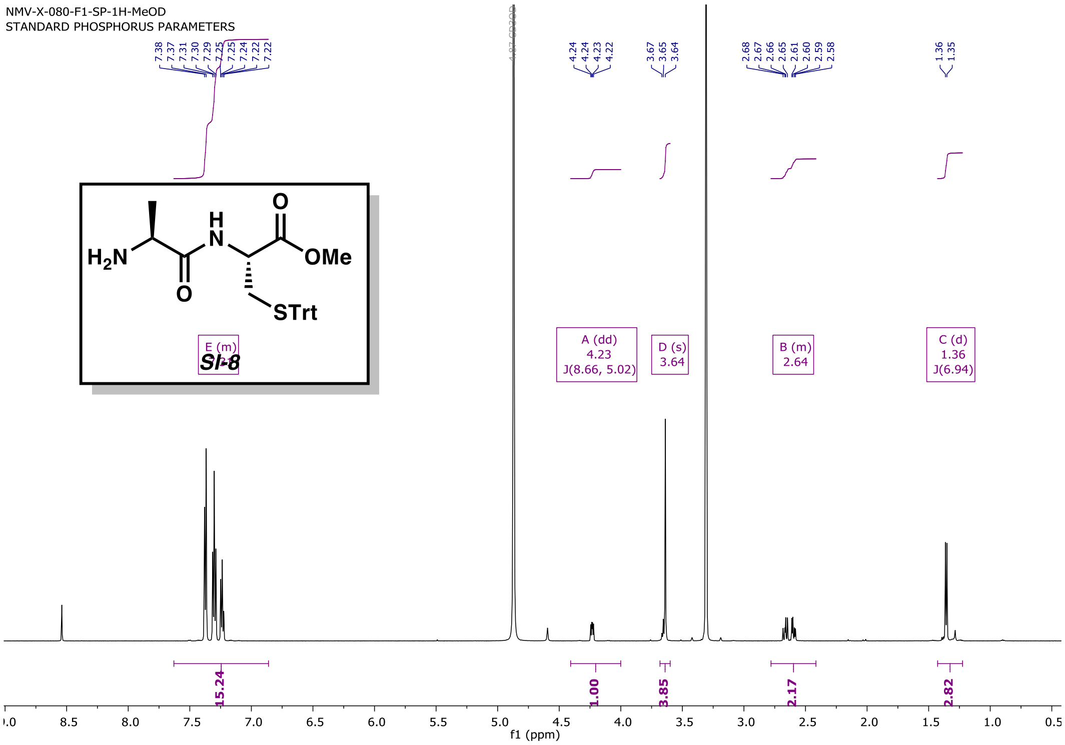

Figure *SI.65*. ¹H NMR (600 MHz, Methanol-d4) of compound ***SI-8*.**

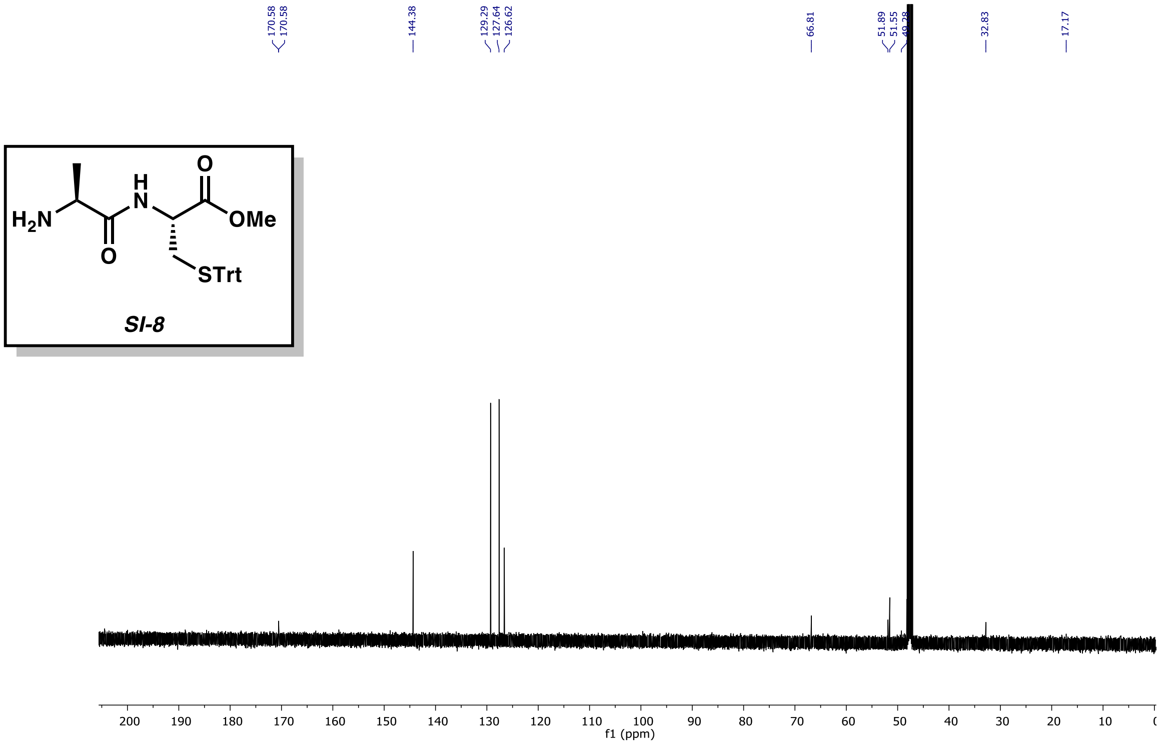

Figure *SI.66*. ¹³C NMR (126 MHz, Methanol-d4) of compound ***SI-8*.**

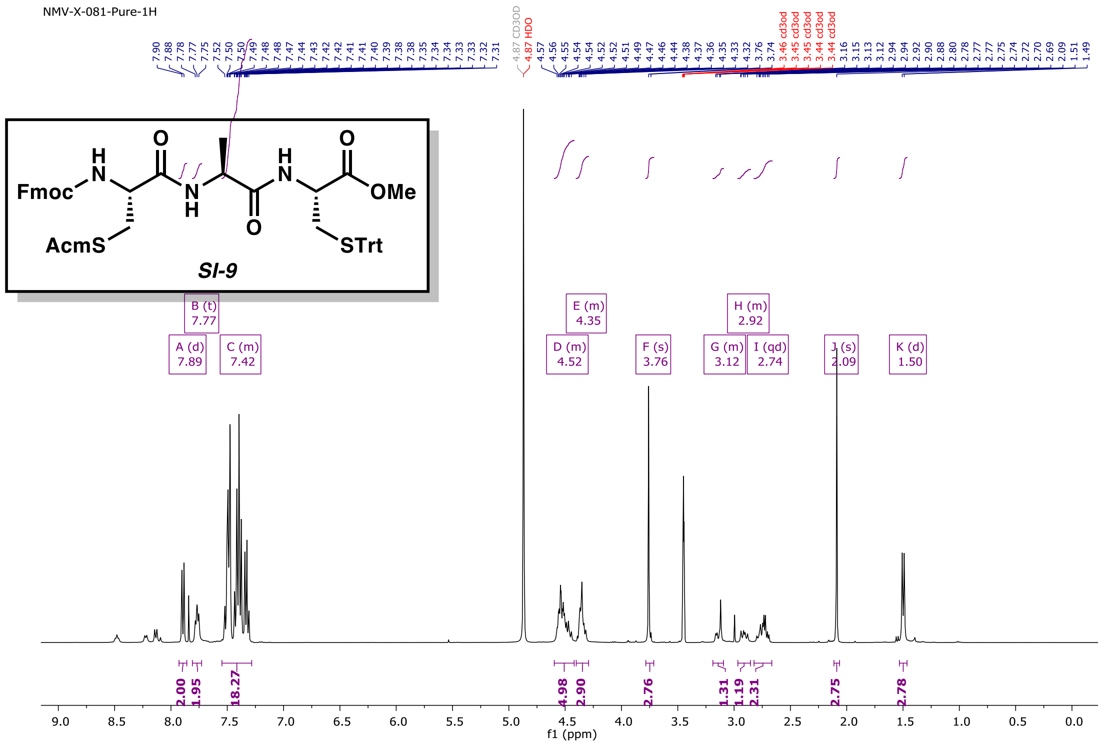

Figure *SI.67*. ^{1}H NMR (400 MHz, Methanol-d_4) of compound ***SI-9***.

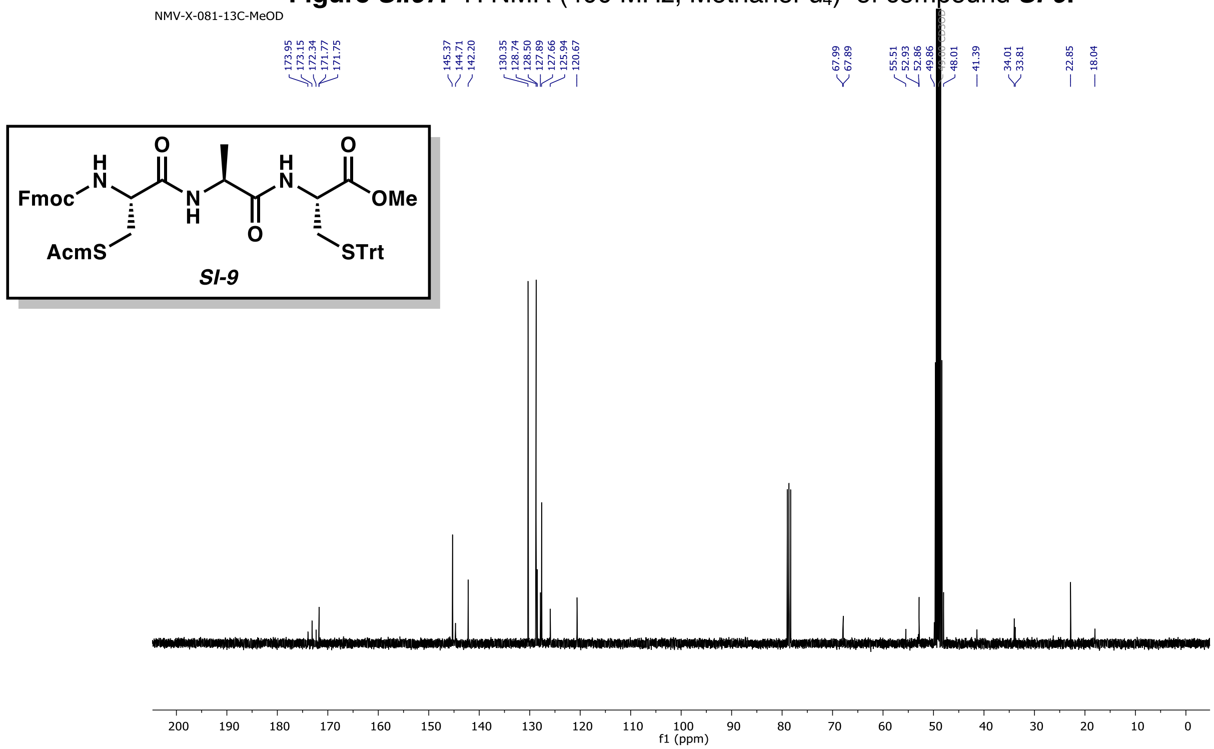

Figure *SI.68*. ^{13}C NMR (101 MHz, Methanol-d_4) of compound ***SI-9***.

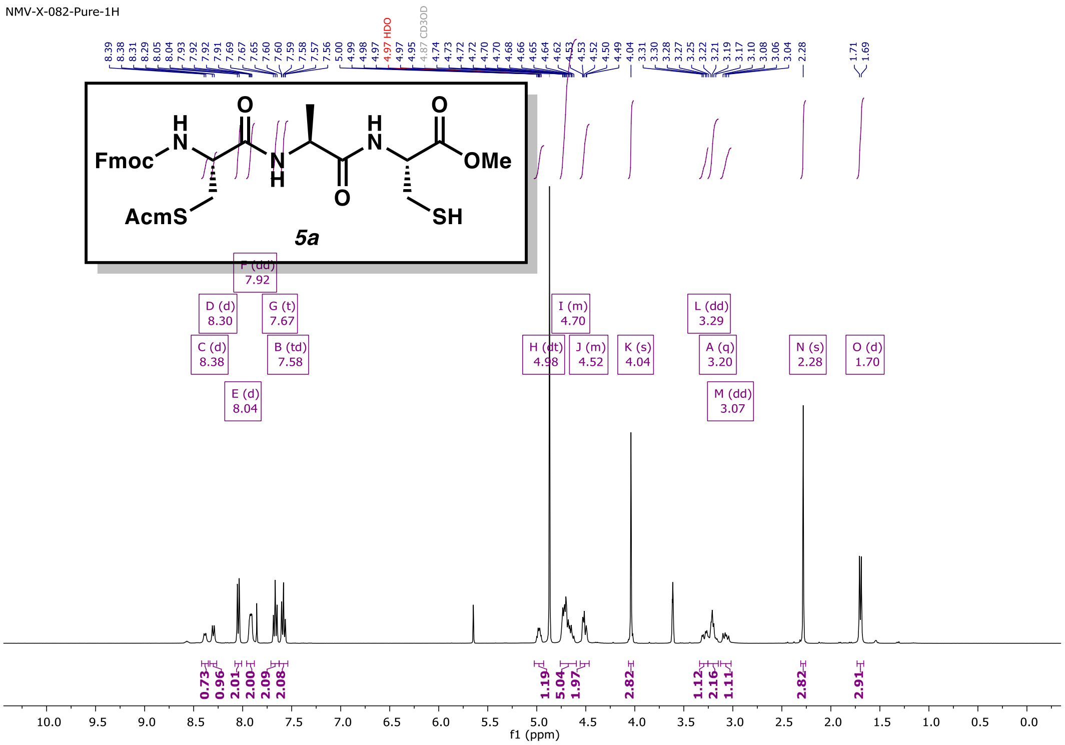

Figure *SI.69*. ^{1}H NMR (400 MHz, Methanol-d_4) of compound ***5a*.**

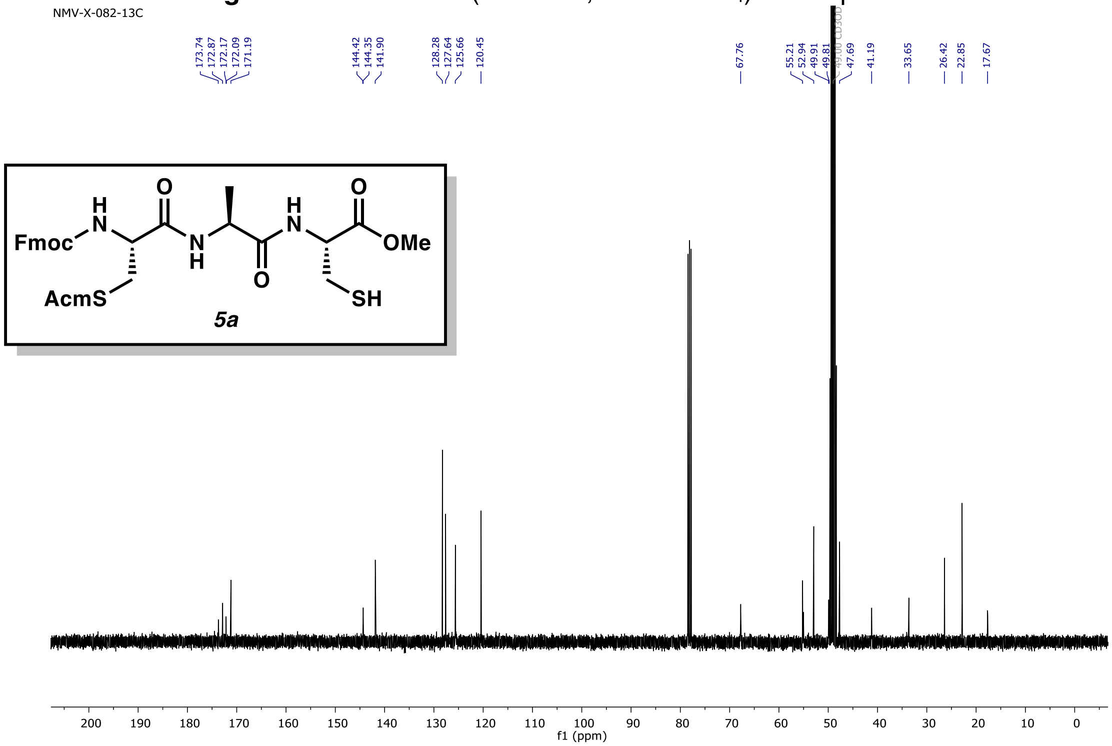

Figure *SI.70*. ^{13}C NMR (126 MHz, Methanol-d_4) of compound ***5a*.**

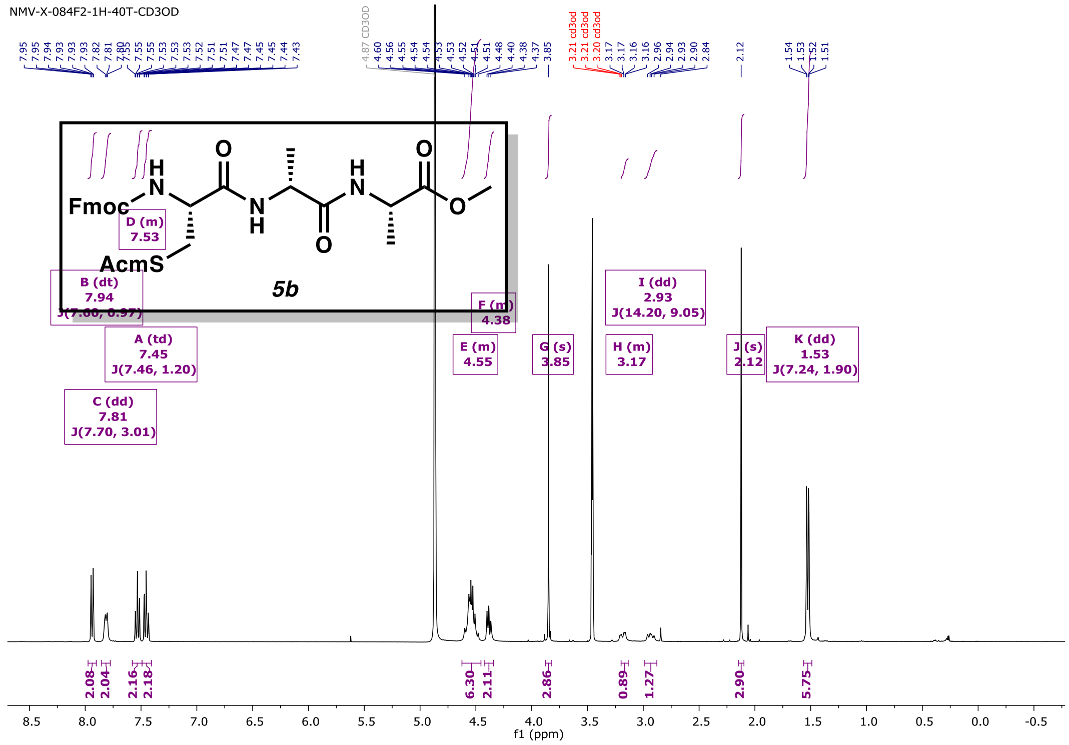

Figure *SI.71*. ^{1}H NMR (400 MHz, Methanol-d_4) of compound ***5b*.**

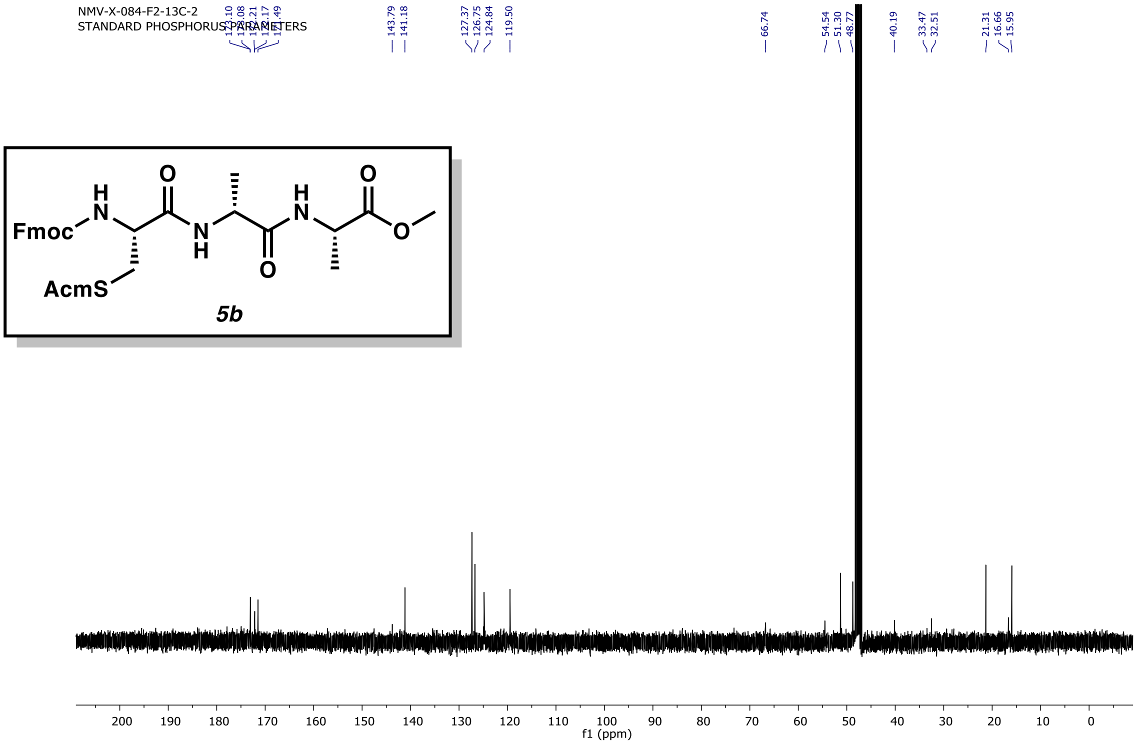

Figure *SI.72*. ^{13}C NMR (101 MHz, Methanol-d_4) of compound ***5b*.**

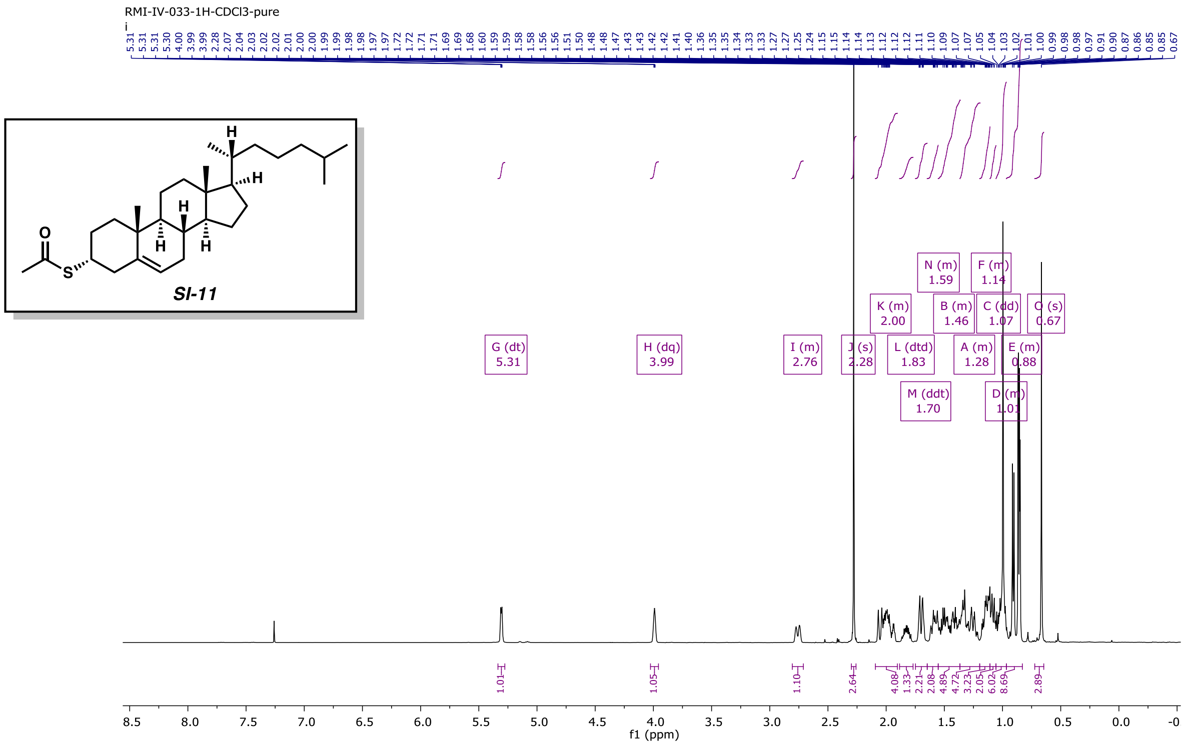

Figure *SI.73*. ^{1}H-NMR (499 MHz, $CDCl_3$) of compound ***SI-11*.**

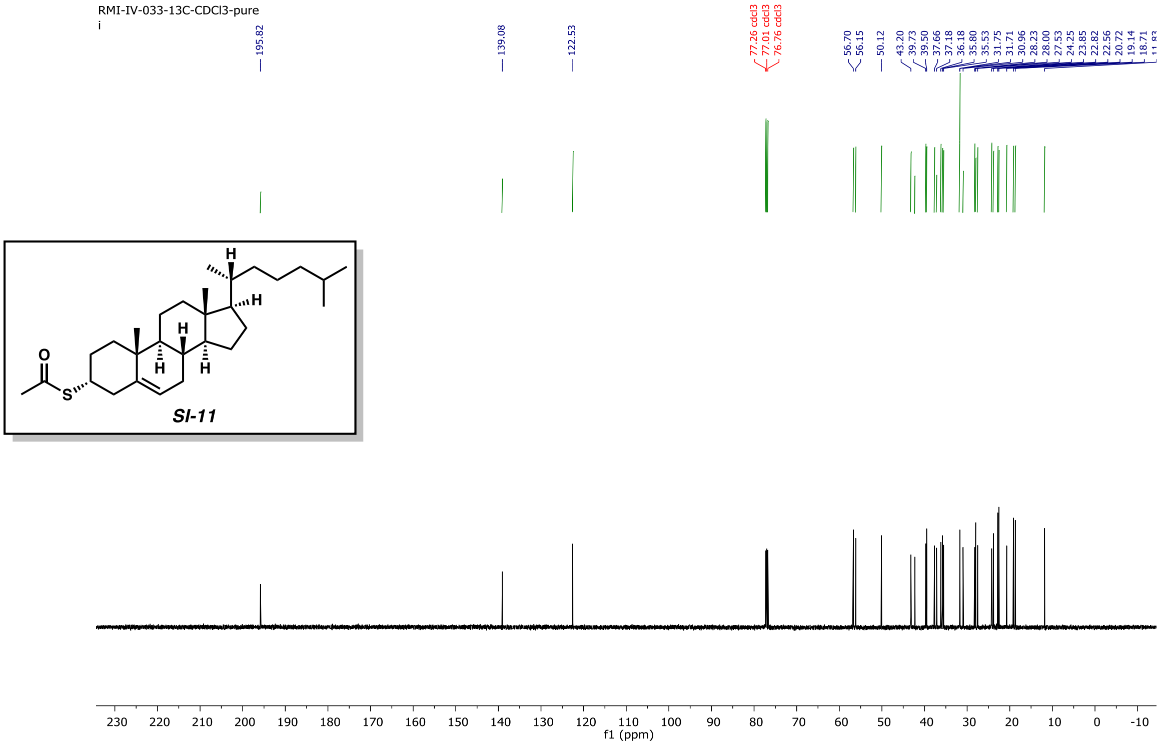

Figure *SI.74*. ^{13}C NMR (126 MHz, $CDCl_3$) of compound ***SI-11*.**

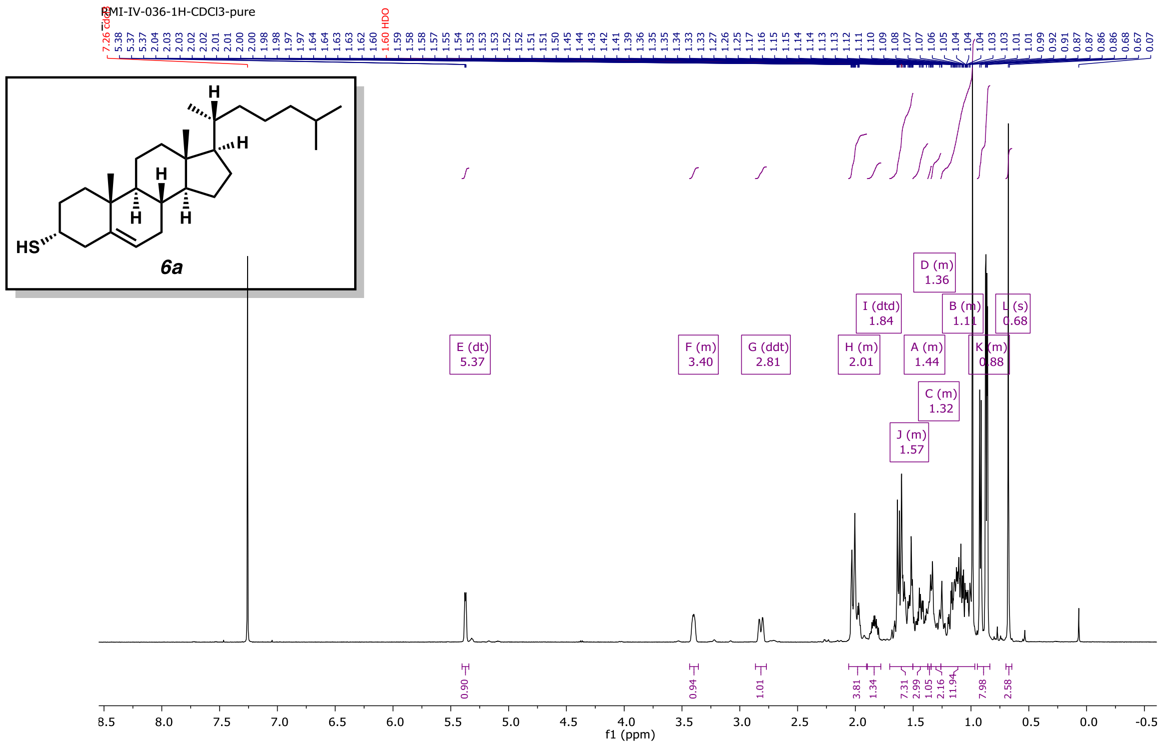

Figure *SI.75.* ^{1}H-NMR (499 MHz, $CDCl_3$) of compound ***6a*.**

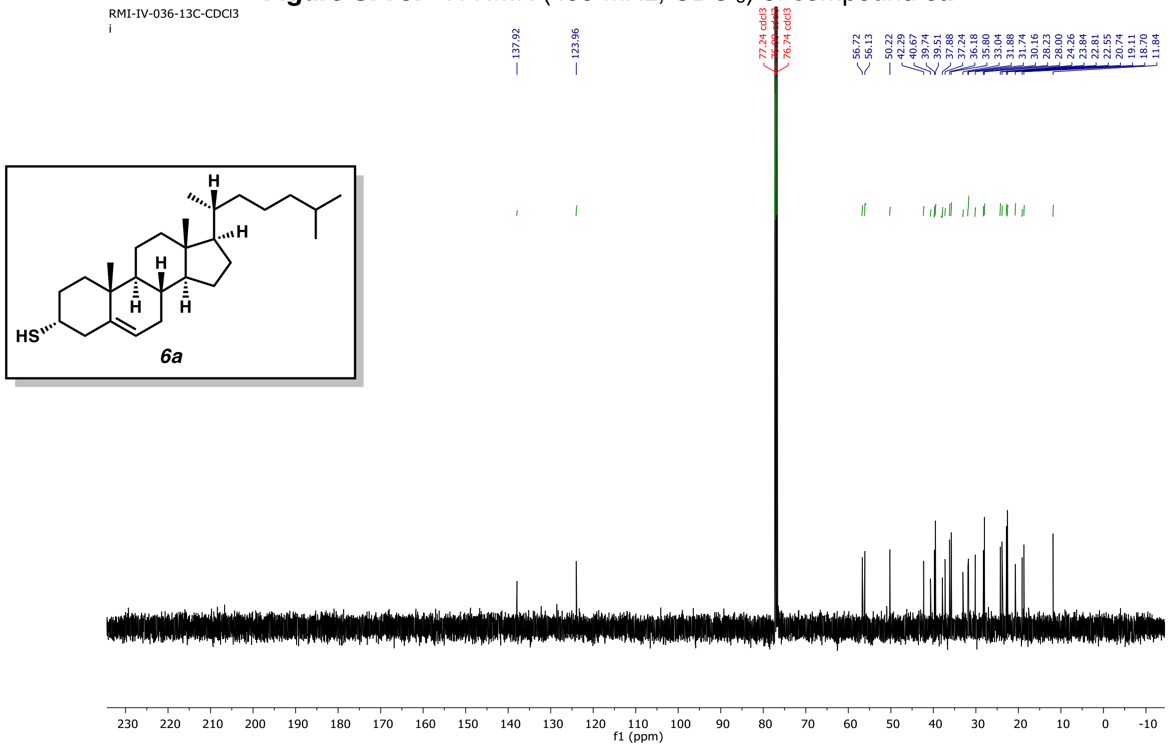

Figure *SI.76.* ^{13}C NMR (126 MHz, $CDCl_3$) of compound ***6a*.**

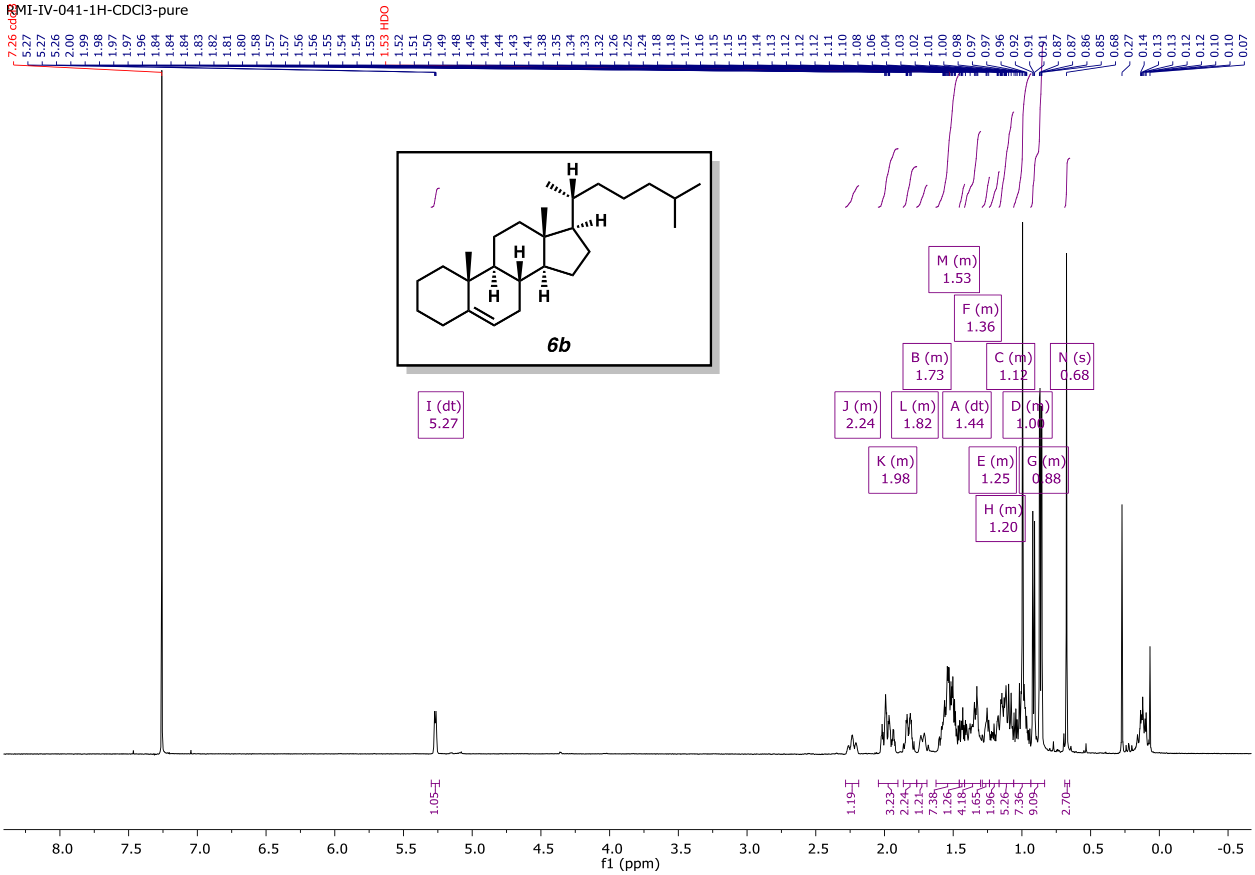

Figure *SI.77*. ^{1}H-NMR (499 MHz, $CDCl_3$) of compound ***6b*.**

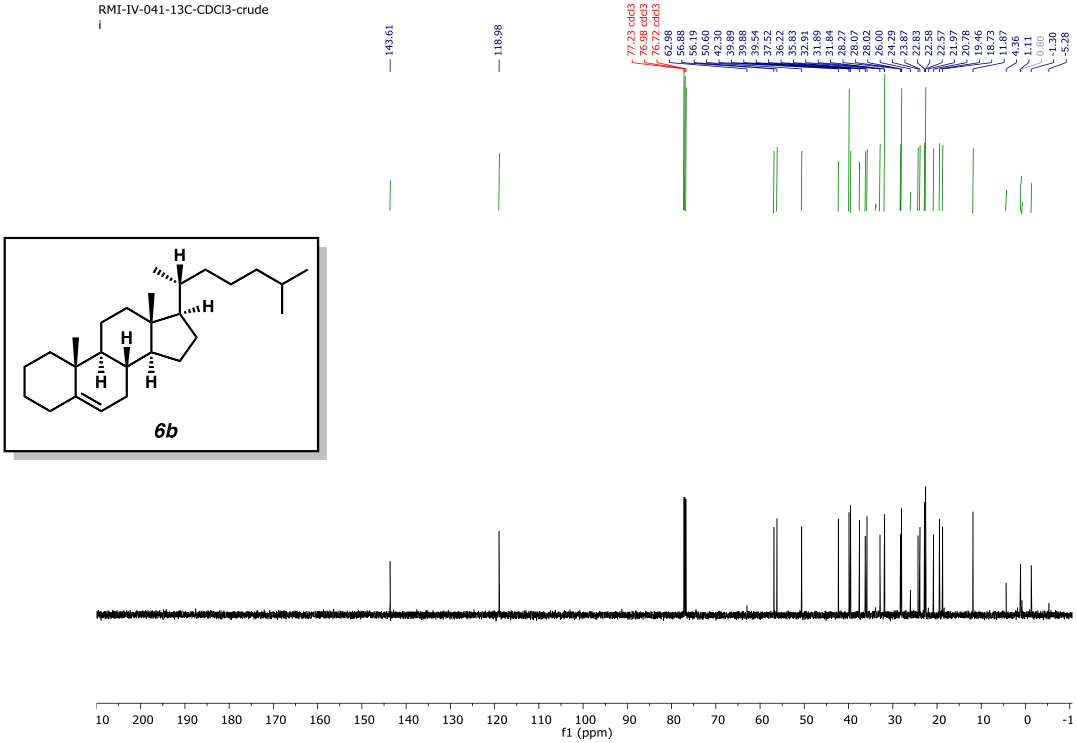

Figure *SI.78*. ^{13}C NMR (126 MHz, $CDCl_3$) of compound ***6b*.**

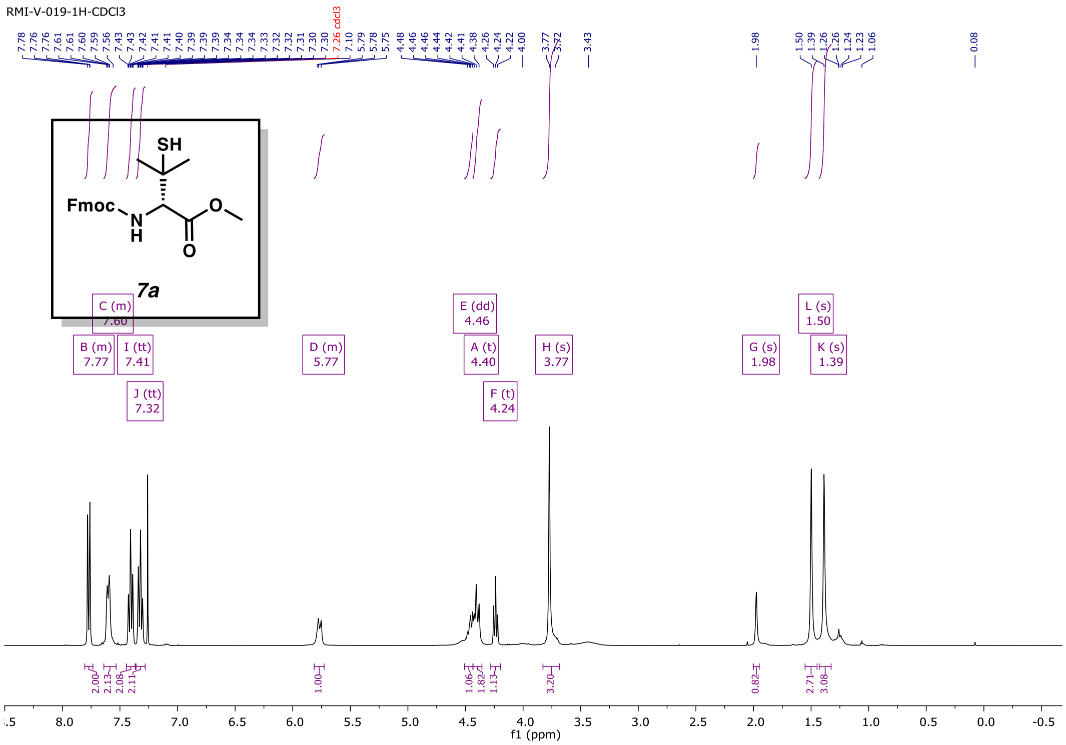

Figure *SI.79.* ^{1}H-NMR (400 MHz, $CDCl_3$) of compound ***7a*.**

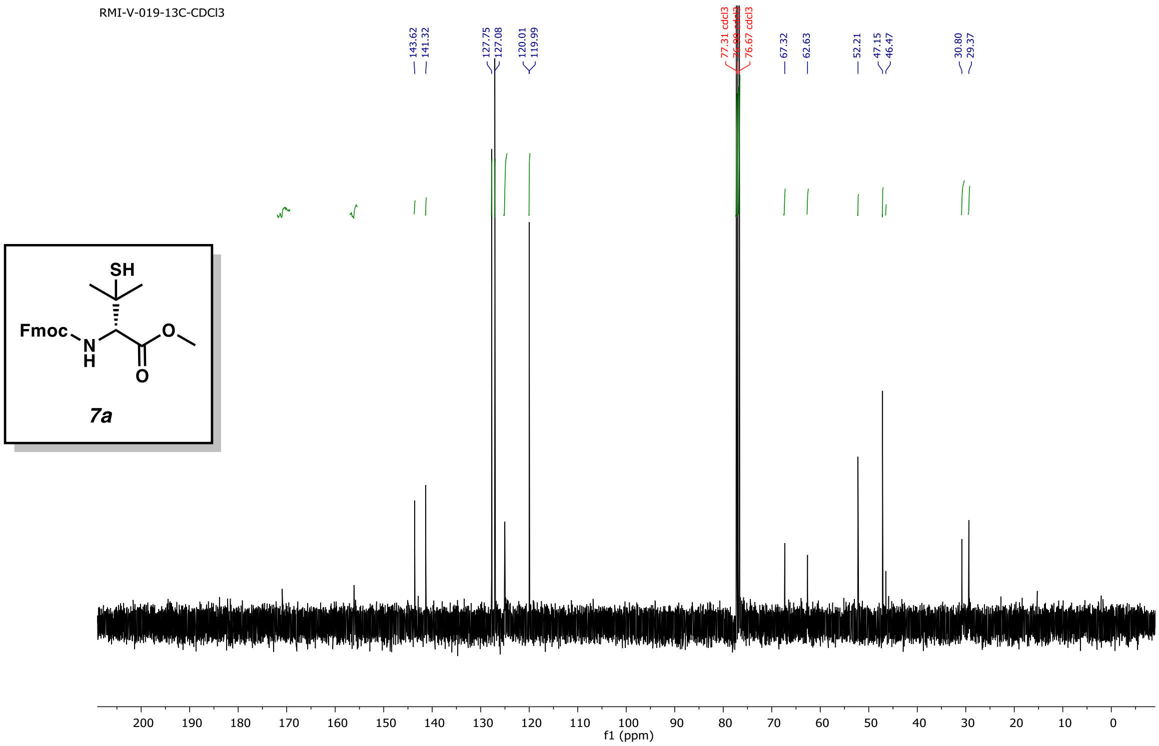

Figure *SI.80.* ^{13}C NMR (101 MHz, $CDCl_3$) of compound ***7a*.**

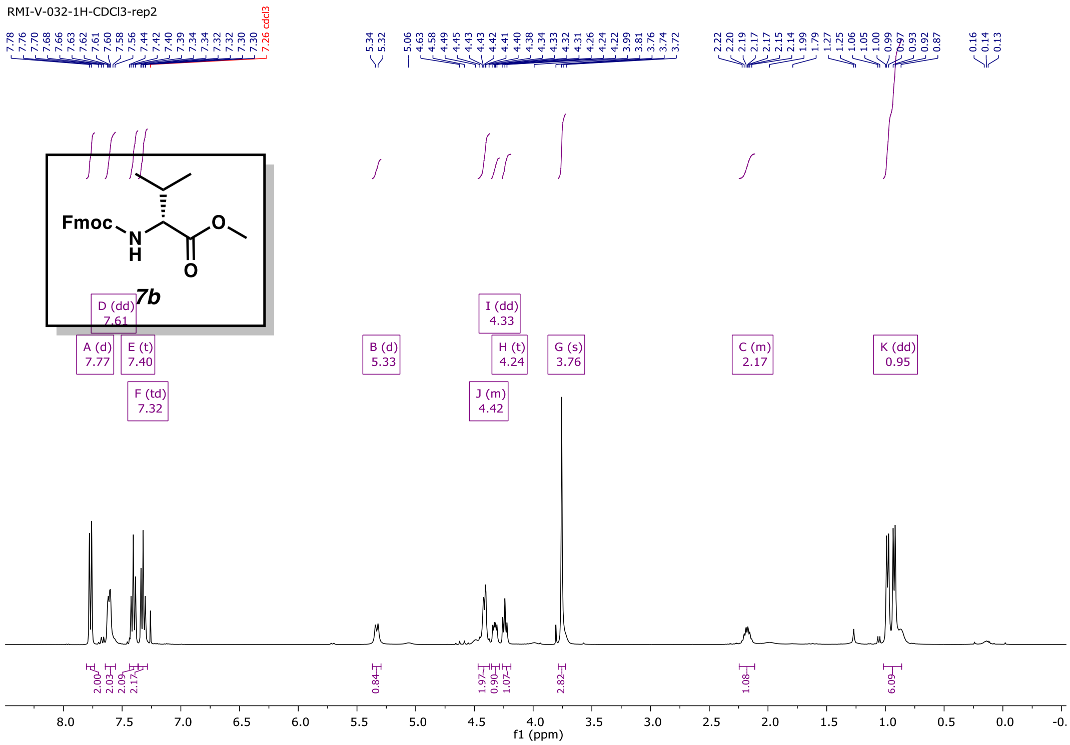

Figure SI.*81*. ¹H-NMR (400 MHz, $CDCl_3$) of compound ***7b*.**

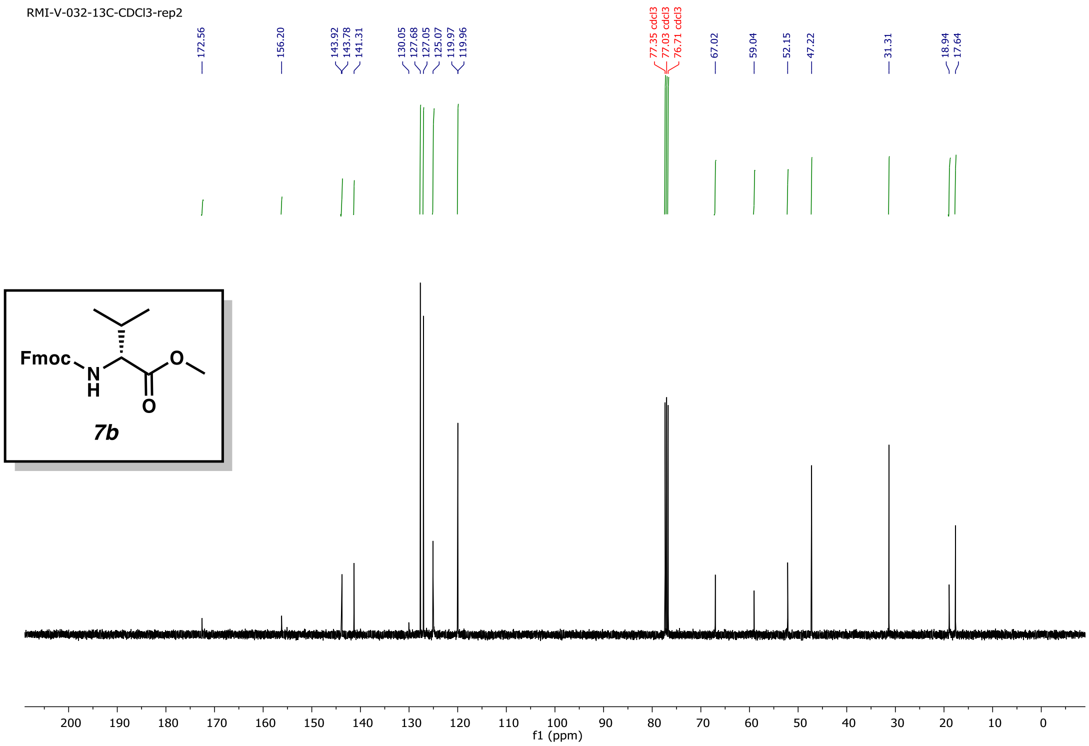

Figure *SI.82*. ¹³C NMR (101 MHz, $CDCl_3$) of compound ***7b*.**

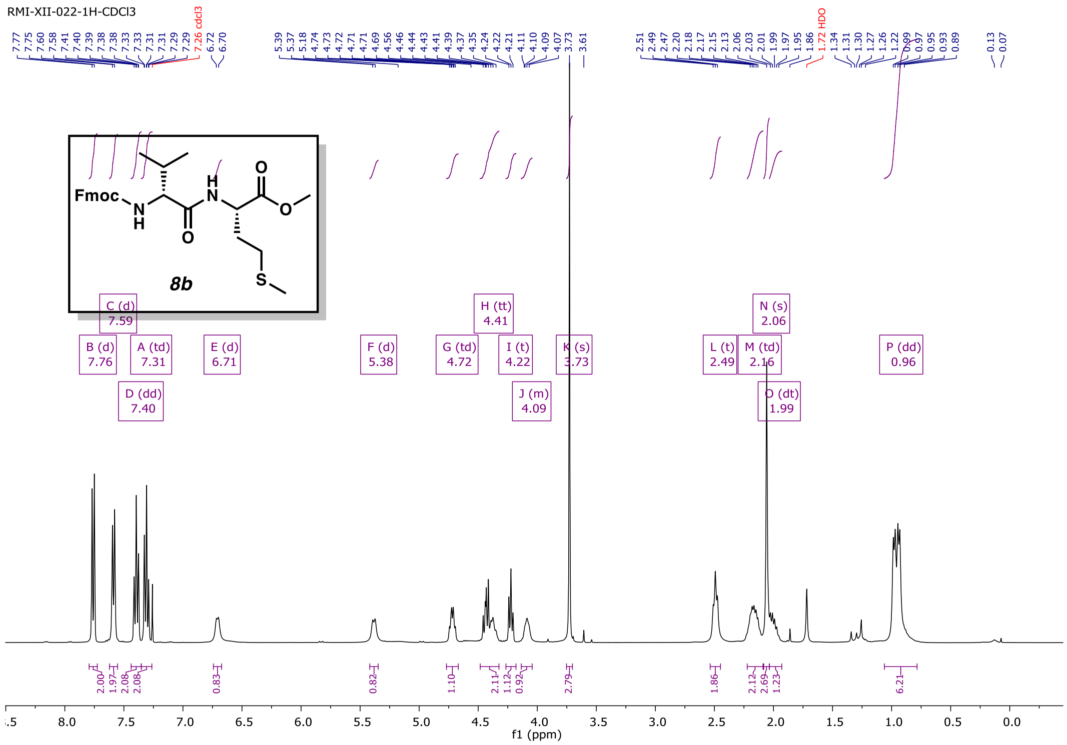

Figure *SI.83*. ^{1}H-NMR (400 MHz, $CDCl_3$) of compound ***8b*.**

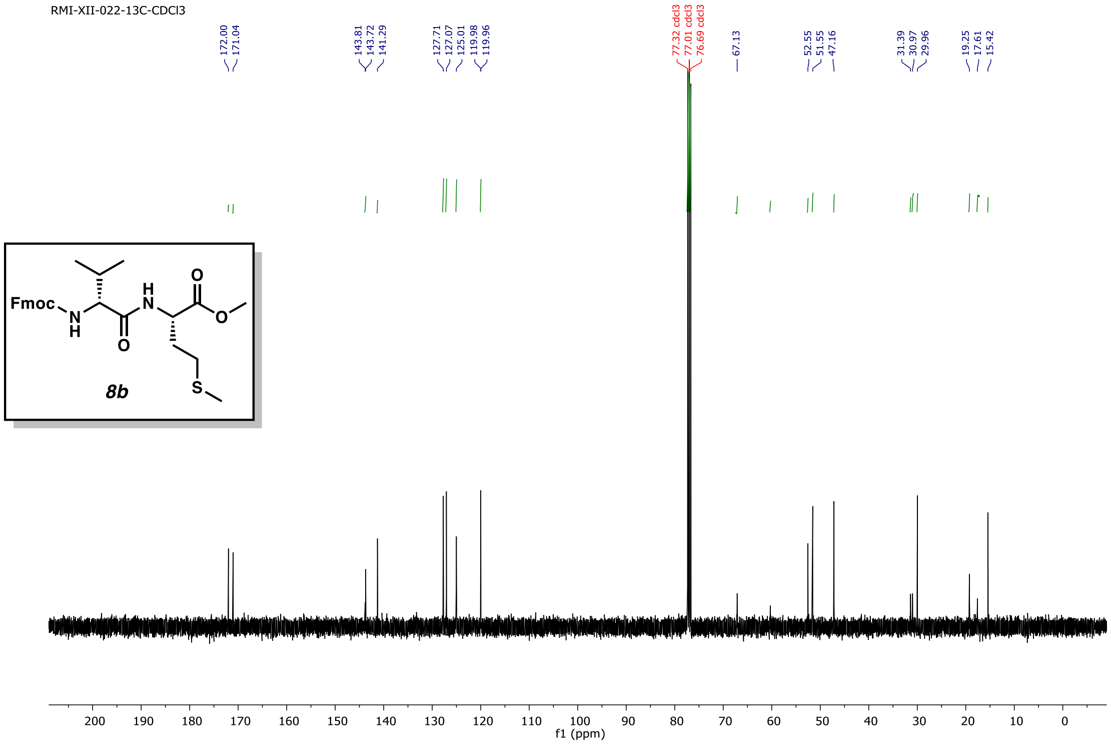

Figure *SI.84*. ^{13}C NMR (101 MHz, $CDCl_3$) of compound ***8b*.**

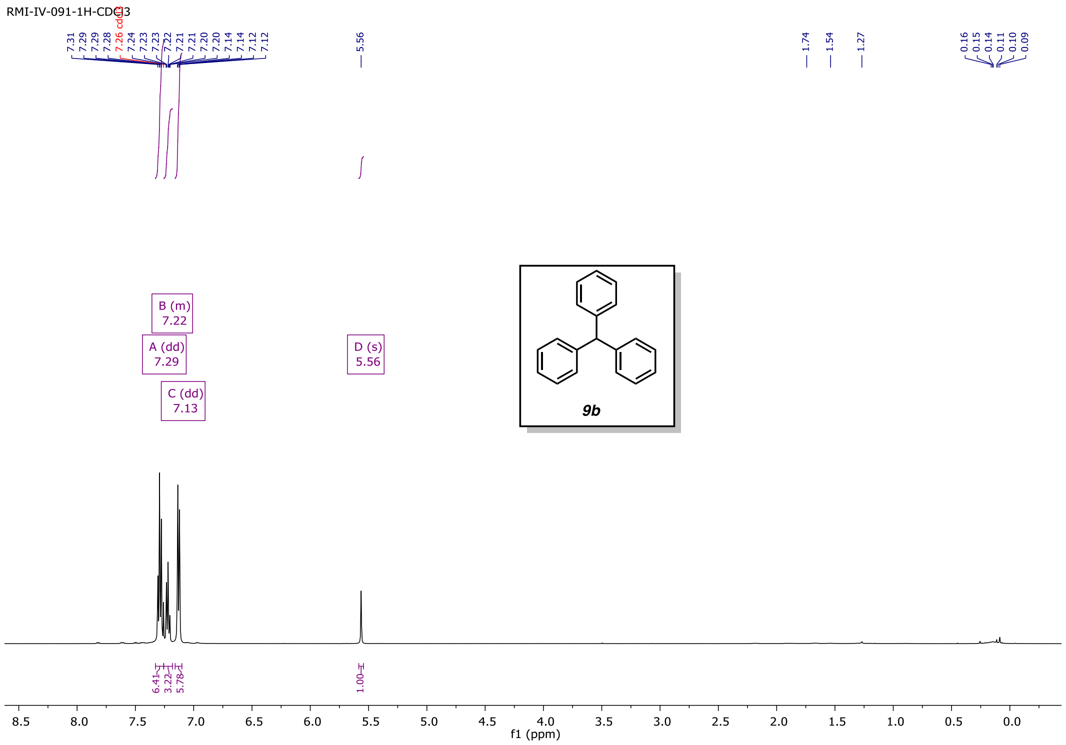

Figure *SI.85*. ^{1}H-NMR (499 MHz, $CDCl_3$) of compound ***9b*.**

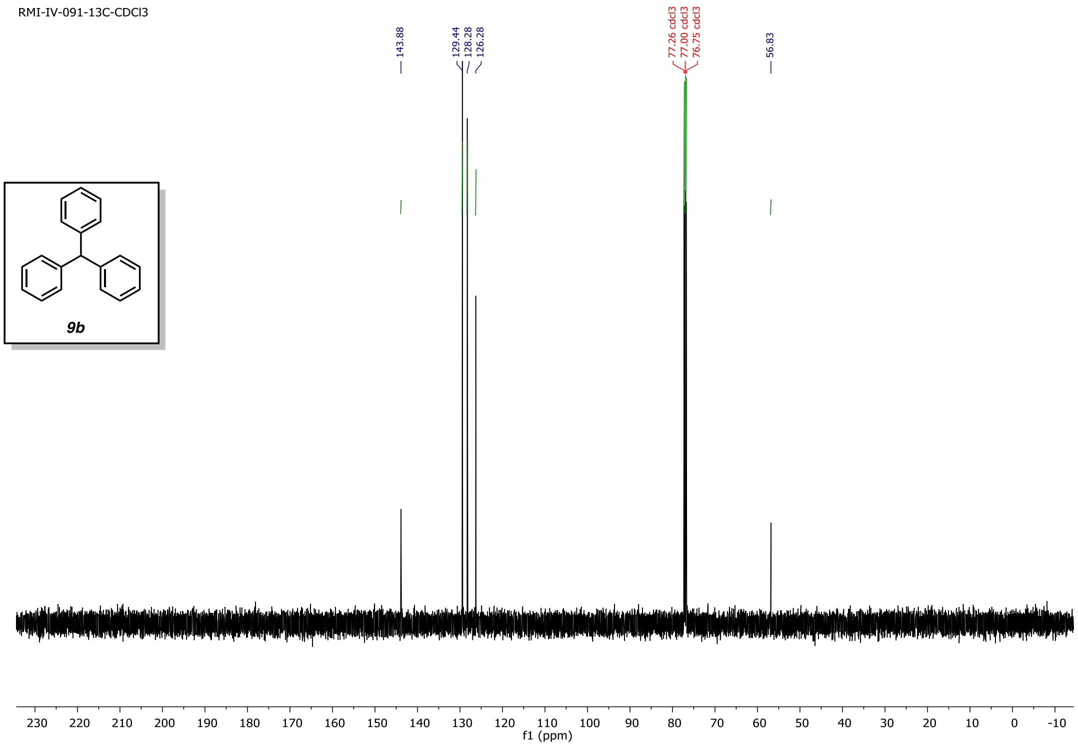

Figure *SI.86*. ^{13}C NMR (126 MHz, $CDCl_3$) of compound ***9b*.**

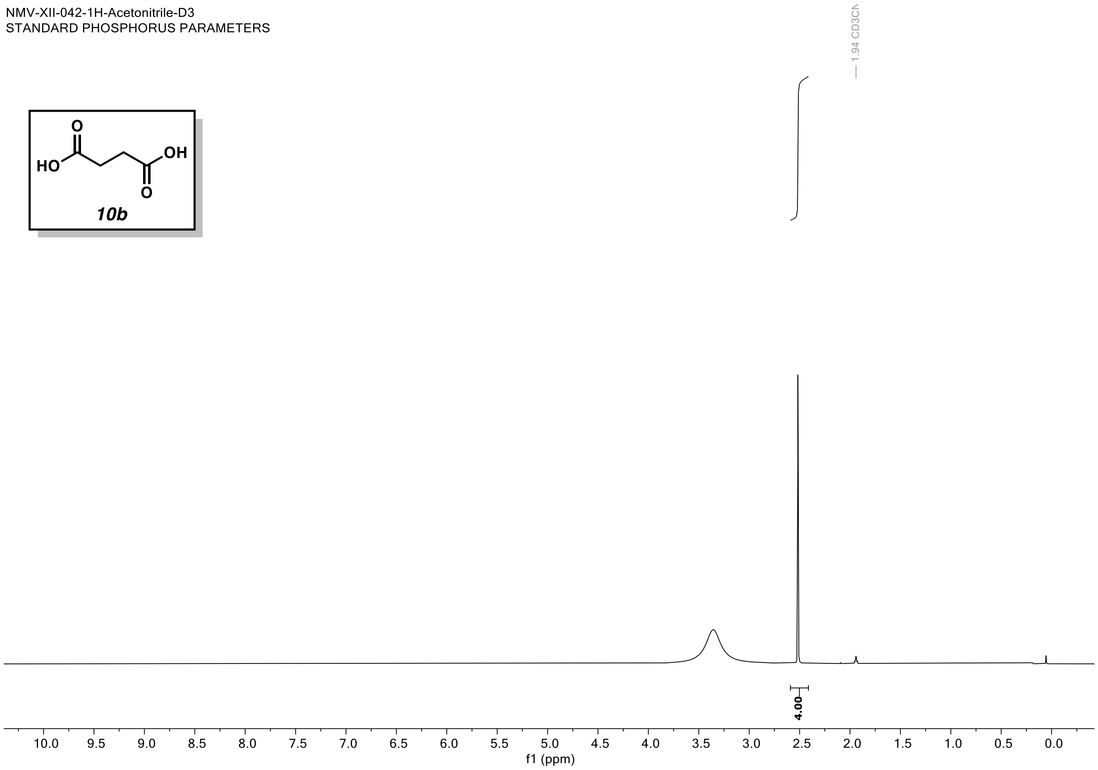

Figure *SI.87*. ^{1}H-NMR (400 MHz, Acetonitrile-d_3) of compound ***10b*.**

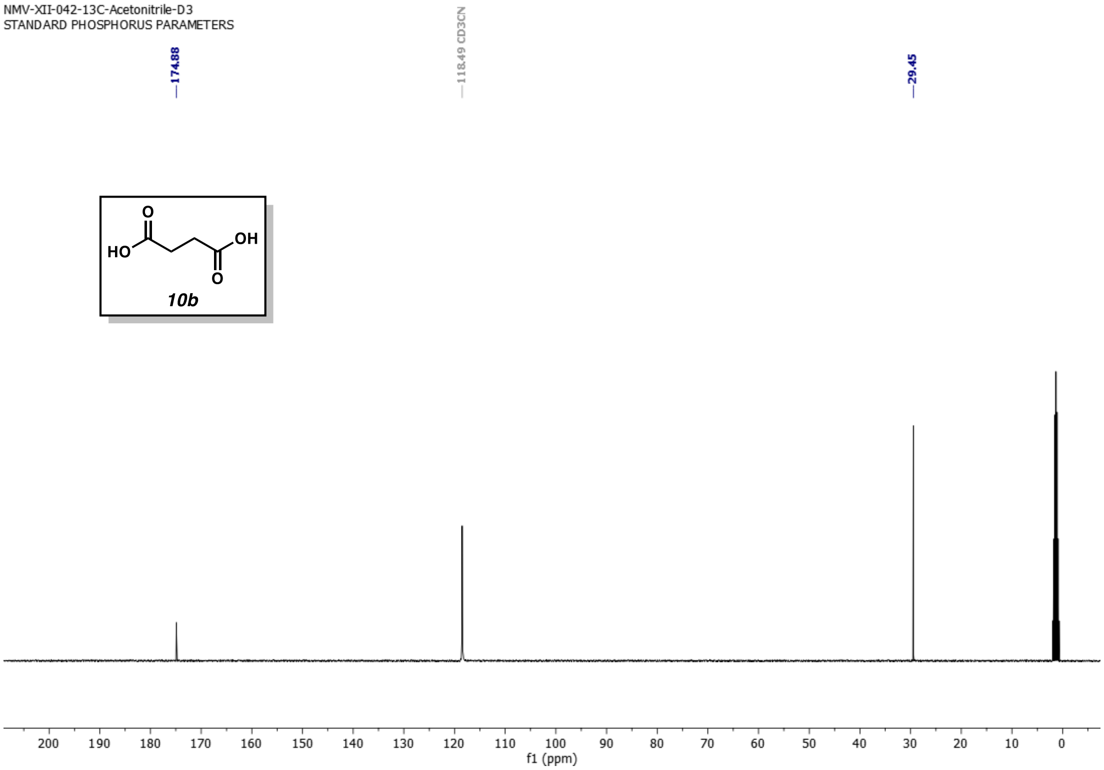

Figure *SI.88*. ^{13}C NMR (101 MHz, Acetonitrile-d_3) of compound ***10b*.**

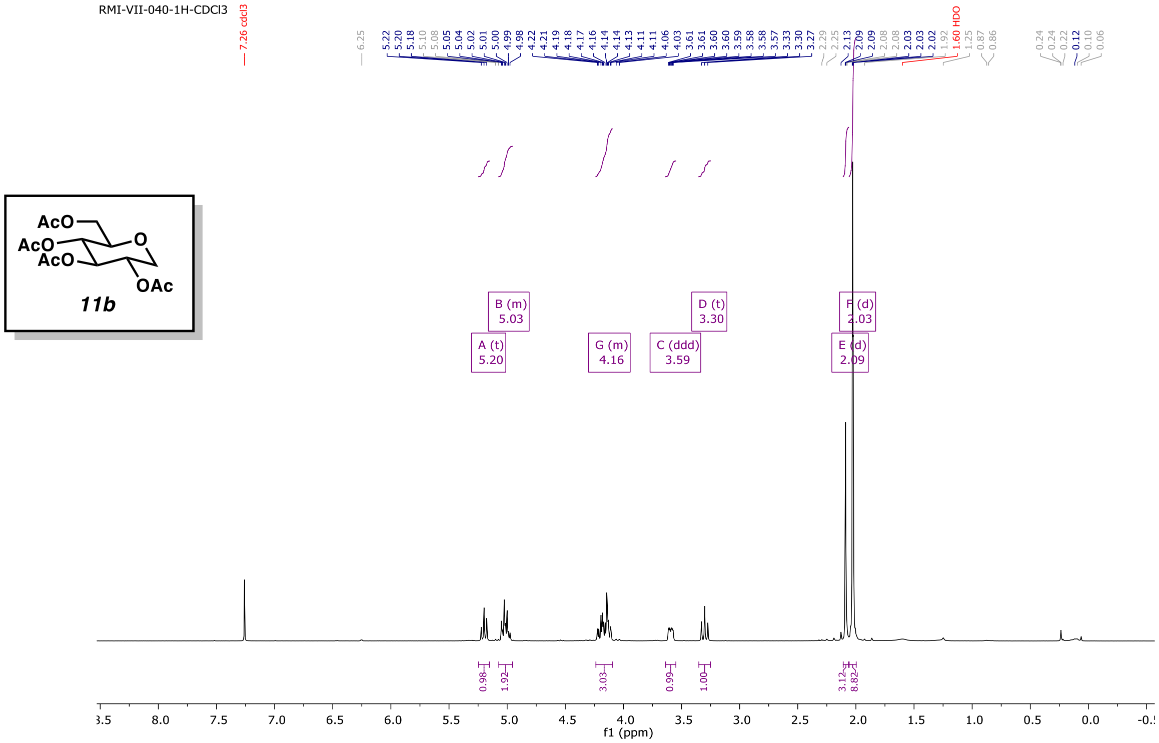

Figure *SI.89*. ^{1}H-NMR (400 MHz, $CDCl_3$) of compound ***11b*.**

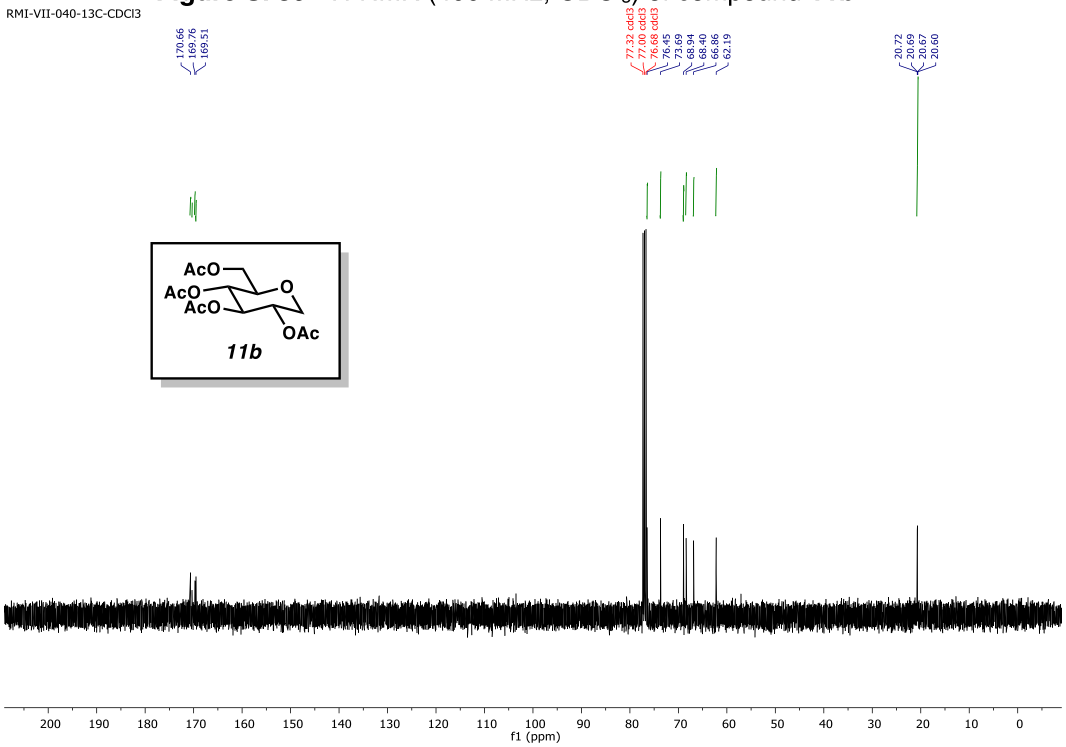

Figure *SI.90*. ^{13}C NMR (101 MHz, $CDCl_3$) of compound ***11b*.**

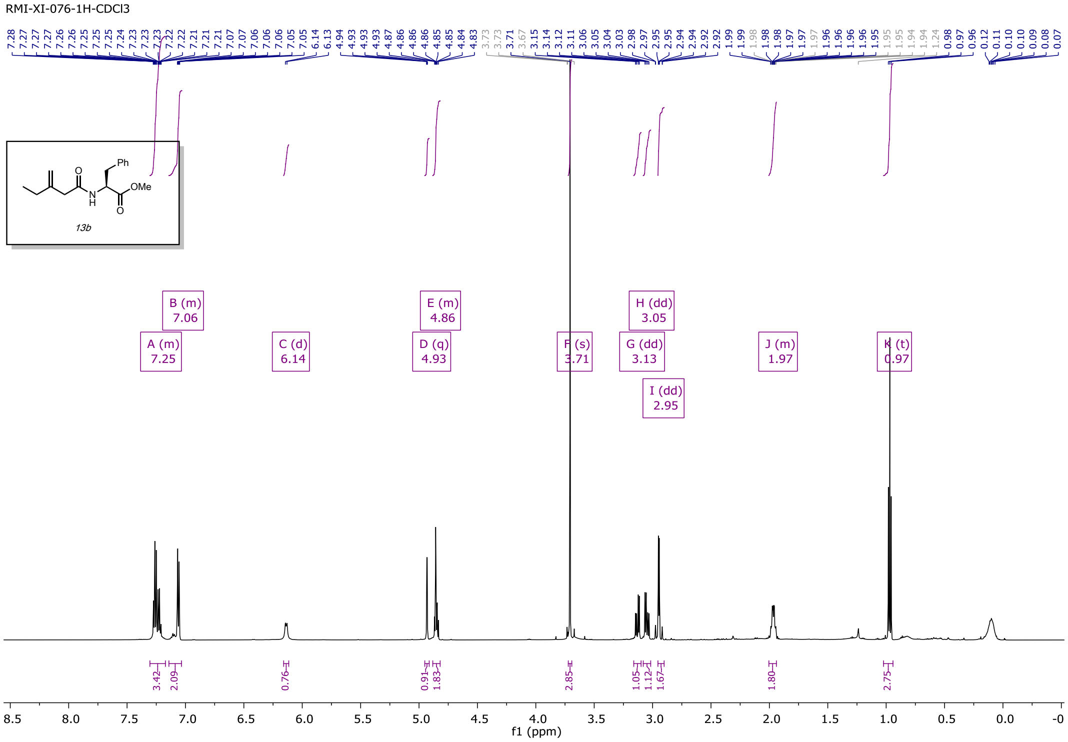

Figure *SI.91.* ^{1}H-NMR (600 MHz, $CDCl_3$) of compound ***13b*.**

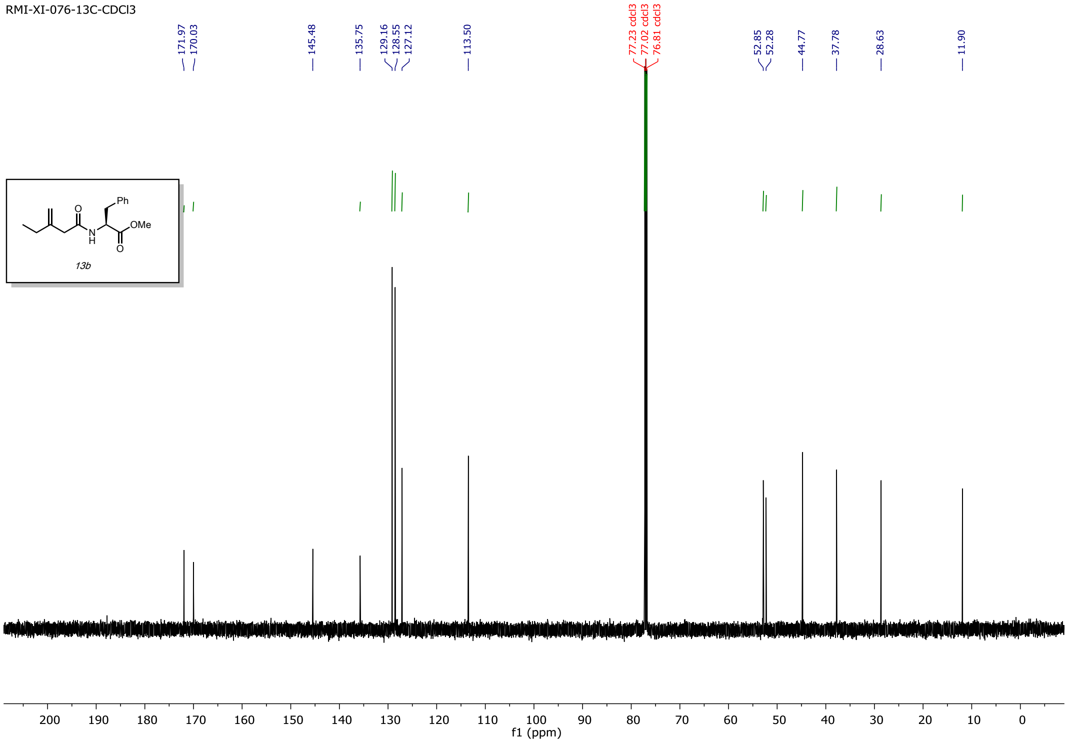

Figure *SI.92.* ^{13}C NMR (151 MHz, $CDCl_3$) of compound ***13b*.**
